# Ab Initio and Kinetic Modeling of β-d-Xylopyranose under Fast Pyrolysis Conditions

**DOI:** 10.1021/acs.jpca.3c07063

**Published:** 2024-02-01

**Authors:** Jacopo Lupi, Leandro Ayarde-Henríquez, Mark Kelly, Stephen Dooley

**Affiliations:** † School of Physics, Trinity College Dublin, Dublin 2, Ireland; ‡ AMBER, Advance Materials and BioEngineering Research Centre, Dublin 2, Ireland

## Abstract

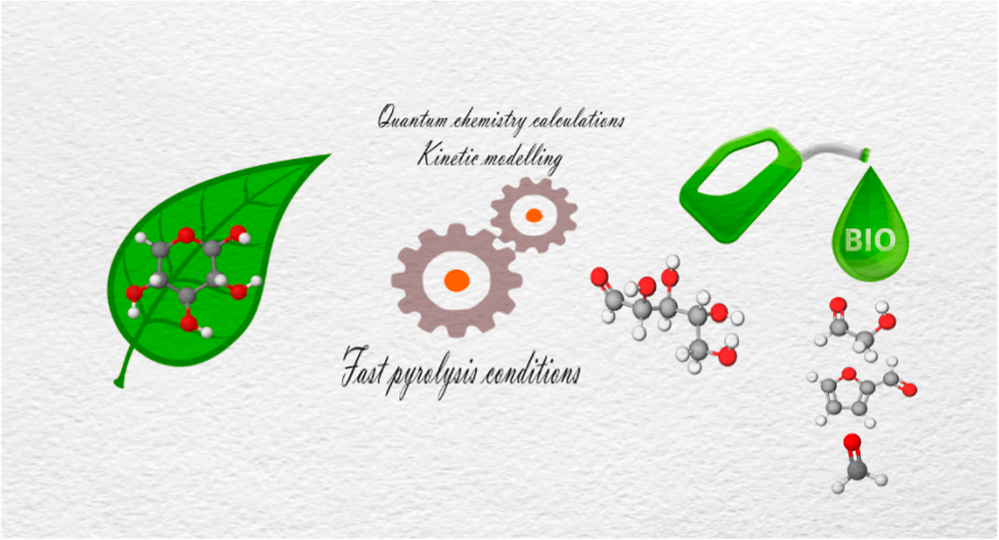

Lignocellulosic biomass is an abundant renewable resource that can be upgraded to chemical and fuel products through a range of thermal conversion processes. Fast pyrolysis is a promising technology that uses high temperatures and fast heating rates to convert lignocellulose into bio-oils in high yields in the absence of oxygen. Hemicellulose is one of the three major components of lignocellulosic biomass and is a highly branched heteropolymer structure made of pentose, hexose sugars, and sugar acids. In this study, β-d-xylopyranose is proposed as a model structural motif for the essential chemical structure of hemicellulose. The gas-phase pyrolytic reactivity of β-d-xylopyranose is thoroughly investigated using computational strategies rooted in quantum chemistry. In particular, its thermal degradation potential energy surfaces are computed employing Minnesota global hybrid functional M06-2X in conjunction with the 6-311++G(d,p) Pople basis set. Electronic energies are further refined by performing DLPNO-CCSD(T)-F12 single-point calculations on top of M06-2X geometries using the cc-pVTZ-F12 basis set. Conformational analysis for minima and transition states is performed with state-of-the-art semiempirical quantum chemical methods coupled with metadynamics simulations. Key thermodynamic quantities (free energies, barrier heights, enthalpies of formation, and heat capacities) are computed. Rate coefficients for the initial steps of thermal decomposition are computed by means of reaction rate theory. For the first time, a detailed elementary reaction kinetic model for β-d-xylopyranose is developed by utilizing the thermodynamic and kinetic information acquired from the aforementioned calculations. This model specifically targets the initial stages of β-d-xylopyranose pyrolysis in the high-pressure limit, aiming to gain a deeper understanding of its reaction kinetics. This approach establishes a systematic strategy for exploring reactive pathways, evaluating competing parallel reactions, and selectively accepting or discarding pathways based on the analysis. The findings suggest that acyclic d-xylose plays a significant role as an intermediary in the production of key pyrolytic compounds during the pyrolysis of xylose. These compounds include furfural, anhydro-d-xylopyranose, glycolaldehyde, and dihydrofuran-3(2*H*)-one.

## Introduction

Biomass pyrolysis refers to the thermal decomposition of organic matter, such as agricultural waste and wood, in the absence of oxygen to produce biofuels, such as bio-oil, charcoal, and biogas. As the demand for energy grows, biomass pyrolysis has emerged as a promising source of clean and sustainable energy. This is because it produces valuable biofuels and has the potential to reduce greenhouse gas emissions by replacing fossil fuels while also minimizing waste. For example, the European Union has set targets to increase the use of renewable energy and reduce greenhouse gas emissions.^1^ This has led to increased investment in biomass pyrolysis technologies, as well as the development of policies and incentives to support their deployment.

In particular, nonfood, nonfeed lignocellulosic plant matter is a highly desirable form of biomass with numerous advantages. Its abundance, widespread availability, and cost-effectiveness make it an attractive option for various applications, owing to its prevalence in crop residues, woody plants, and grasses. Chemicals, fuels, and energy must be produced from lignocellulosic plant matter in order to decarbonize our global economies, and, to be successful, these lignocellulose-derived products must compete in quality and price with fossil-derived products. Lignocellulose is formed by three main components, namely, cellulose, hemicellulose, and lignin.

Hemicellulose is the second most abundant component of lignocellulosic biomass and is a group of cell wall polysaccharides distinct from cellulose and pectin. Some examples of hemicelluloses include xylans (such as β-1,4-d-xylan, β-1,3-d-xylan, β-1,3;1,4-d-xylan, 4-*O*-methyl-d-glucorono-d-xylan, glucuronoxylan, arabinoxylan, glucuronoarabinoxylan, and arabinoglucuronoxylan), mannans (like homomannan, galactomannan, glucomannan, and galactoglucomannan), xyloglucans, β-1,3;1,4-glucans, and galactans (such as sulfated galactans and arabinogalactans). The main hemicellulose polysaccharides found in hardwood, softwood, and herbaceous biomass are 4-*O*-methylglucoronoxylans, galactoglucomannan, and arabinoxylans, respectively. A comprehensive review on hemicellulose pyrolysis has been published in 2017 by Broadbelt and collaborators.^2^

Zhou et al.^3^ proposed one of the first mechanistic models for fast hemicellulose pyrolysis. This model, based on the experimentally characterized composition and linkages of extracted hemicellulose, rationalizes more than 500 reactions involved in hemicellulose decomposition and the formation of various pyrolysis products using the reaction family approach. Additionally, the mechanistic model was extended to simulate fast pyrolysis of native hemicellulose from corn stalks. Their results show that native hemicellulose yields more char, gaseous species, acetol, and acetic acid compared to corn stalk-extracted hemicellulose, while also yielding lower amounts of certain products. The presence and distribution of acetyl groups in hemicellulose significantly influence the pyrolysis product distribution, suggesting the potential impact of biomass pretreatment on pyrolysis outcomes.

Integrating physicochemical information robustly and coherently is crucial to enhancing and expanding kinetic models for lignocellulose pyrolysis. This valuable data can be obtained through computational chemistry techniques. Particularly, quantum chemistry modeling can give many details at the atomic/molecular level. It is usually performed to simulate the pyrolysis of the individual principal biomass components, interactions within/between these materials, and catalytic pyrolysis with various catalysts. On the basis of the theoretical models in quantum chemistry modeling, geometric structures, transition states, intermediates, corresponding electron transfers, orbital interactions, energetics, and other important information involved in the pyrolysis process can be obtained. Quantum chemistry modeling is playing increasingly important roles in the lignocellulosic biomass pyrolysis mechanism studies due to advancement of theoretical methods and computer hardware as well as the experimental limitations. In the literature, many reviews on the experimental studies of lignocellulosic biomass pyrolysis have been reported, and the topics cover the development of pyrolysis techniques,^4,5^ reactors,^6^ reaction conditions,^7^ selective preparation of high-grade bio-oil, and value-added chemicals.^8,9^ As for the pyrolysis mechanism of biomass, most of the reviews focus on the pyrolysis kinetics, especially on global kinetic models.^10−12^

However, most literature on biomass pyrolysis using quantum chemical modeling has been found to have a low-standard quantum chemical bias due to the absence of well-established and robust protocols in the field. The use of outdated functionals (such as B3LYP) simply due to their established history rather than their demonstrated effectiveness is an issue that has been highlighted by Grimme and his colleagues in their recent works.^13,14^ This low-quality quantum chemical modeling can introduce a significant level of uncertainty in computed properties (in particular geometries and reaction barriers), especially when these data are incorporated into global kinetic schemes to reproduce pyrolysis experiments. Therefore, developing new, more physically based protocols for accurate and cost-effective characterization of lignocellulosic pyrolytic systems is essential.

Numerous studies have been conducted using quantum chemistry to investigate the pyrolytic pathways of xylopyranose. However, these studies lack the desired level of comprehensiveness and computational accuracy. For instance, Wang et al.^15^ explored furfural formation, an important biofuel precursor, in both gas and water environments using B3LYP/6-31++G(d,p) and CPCM solvation models. They identified xylulose as an intermediate leading to furfural formation and proposed a feasible pathway involving hydrogen migrations and dehydrations, with solvent effects playing a significant role in stabilizing reactants and transition states.

In another work, Huang et al.^16^ used M06-2X/6-31++G(d,p) to examine xylopyranose pyrolysis pathways, highlighting reactions leading to acyclic-containing carbonyl isomer (xylose) through immediate ring opening, as well as pathways involving dehydration reactions. The major products obtained were low molecular weight compounds, including glycolaldehyde, 2-furaldehyde, acetaldehyde, and acetone, while other competitive products like formaldehyde, formic acid, acetic acid, CO_2_, CH_4_, acetol, and pyranone were also identified.

In 2019, Hu et al.^17^ conducted a comprehensive investigation of xylopyranose thermal decomposition, employing analytical pyrolysis-gas chromatography/mass spectrometry (Py-GC/MS) and quantum chemistry calculations using B3LYP-D3/6-311G(d,p). They studied xylopyranose, its dimer, and xylan, finding that acyclic xylose played a key role as an intermediate in the formation of major pyrolytic products, such as 1,4-anhydro-d-xylopyranose, furfural, and glycolaldehyde during xylose pyrolysis.

Despite the considerable efforts made by multiple authors to study the pyrolytic pathways of β-d-xylopyranose using quantum chemistry, their investigations were limited by the use of an unsatisfactory quantum chemical level of theory and the absence of a detailed kinetic model to explore the interplay between thermodynamics and kinetics. As a result, in this study, the goal is to revisit the β-d-xylopyranose pyrolytic reactions, building upon the existing literature but employing a higher level of theory that is both robust and well-established, and provide thermochemical and kinetic data that can be valuable for the biomass modeling community.

The computational strategy employed comprises four fundamental components: (i) the characterization of stationary points within the reactive potential energy surface using the density functional theory; (ii) the refinement of electronic energies, vibrational zero-point energies, and determination of thermodynamic quantities; (iii) the determination of rate constants through the application of reaction rate theory; and (iv) the construction of a kinetic model that integrates thermodynamic and kinetic aspects to analyze their mutual influence. A perspective on a general computational strategy to iteratively investigate biomass-related chemical systems is also sketched. The manuscript is organized as follows: first, a comprehensive exposition of the computational methods utilized in this paper is provided. Subsequent sections include the presentation of the computed reaction mechanism and an accompanying analysis of the associated thermodynamic properties. Additionally, the discussion encompasses the rate constants calculated via transition state theory. Finally, the outcomes of the kinetic model simulations are elucidated. The last section is reserved for summarizing the findings and outlining potential future directions.

## Computational Methodology

### Electronic Structure Calculations

The potential energy surfaces of β-d-xylopyranose thermal degradation reaction mechanism have been investigated through high-level quantum chemical calculations.

On the grounds of its well-known robustness,^13,14,18^ geometry optimizations and harmonic frequencies of reactants, transition states, intermediates, and products along the reaction pathways were obtained by the M06-2X^19^ global hybrid density functional in conjunction with the 6-311++G(d,p) basis set.^20,21^ The stationary points on the reaction pathways were characterized as minima (reactants, intermediates, and products) and saddle points (transition states) based on vibrational frequency calculations. The transition states obtained were further confirmed using intrinsic reaction coordinate (IRC) scans at the same levels of theory.

Single-point energy calculations were conducted on top of DFT geometries to further refine electronic energies at the DLPNO-CCSD(T)^22^ (with F12 explicit correlation correction)^23^ level of theory in conjunction with the cc-pVTZ-F12 basis set.^24^ DLPNO calculations were performed employing the ORCA code^25^ and using the tightPNO cutoff.

The neglect of anharmonicity tends to overestimate the vibrational zero-point energy (ZPE).^26^ Therefore, in order to obtain accurate zero-point energies, avoiding the calculation of perturbative anharmonic corrections, ZPE and frequencies are scaled by 0.970.^27,28^

Quasi-harmonic entropies were calculated employing Grimme’s approximation.^29^ Indeed, the rigid-rotor harmonic oscillator (RRHO) model proves inadequate in the presence of significant anharmonic motions within a molecule. Typically, anharmonicities arise when internal torsional motion encounters hindrance from a potential barrier comparable to the average molecular thermal energy. The detailed investigation of the impact of internal torsional motions on molecular entropy has been conducted extensively by Pitzer and Gwinn.^30^ Grimme’s treatment has been favored over the one-dimensional hindered rotor model^31^ due to its immediate applicability to a broad range of molecular species. Thermochemical analysis was conducted using two codes: GoodVibes^32^ and Shermo.^33^ The former code was used for enthalpy, entropy, and free energy calculations, while the Shermo code was specifically utilized for calculating constant pressure heat capacities. Given the high flexibility of the molecules under investigation, conformational analysis for minima and transition states is performed with the CREST program^34^ in order to provide starting guesses for the potential energy surface calculations. The code couples state-of-the-art semiempirical quantum chemical methods (GFNn-xTB)^35^ with metadynamics simulations.^36^ A systematic validation process for the conformational ensemble produced by CREST has been employed. This involved selecting conformers with energies lying within approximately 12 kJ mol^–1^ interval from the lower one and subsequently reoptimizing them using the M06-2X/6-311++G(d,p) level of theory. Following this, with a reasonable level of confidence, the lowest energy conformer is identified. All DFT geometry optimization and frequency calculations have been performed using the Gaussian code.^37^

### Enthalpies of Formation Calculation

Taking xylopyranose as an example, the enthalpies of formation (Δ_f_*H*°) of the molecular species were calculated using the enthalpies of atomization as^38^
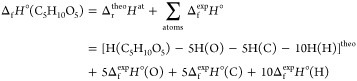


In this expression, theo refers to enthalpies calculated theoretically and exp to “experimental” values. Subscripts f and r refer to formation and reaction, respectively, calculated or measured at 298.15 K. Atoms are considered in their standard state, and their enthalpies of formation are taken from the Active Thermochemical Tables (ATcT),^39−41^ namely, H(^2^S) = 217.998 kJ mol^–1^, C(^3^P) = 716.881 kJ mol^–1^, and O(^3^P) = 249.229 kJ mol^–1^.

The coupled cluster  diagnostic^42^ has been found to be lower than 0.02 for closed-shell molecules and smaller than 0.04 for open-shell species (see the Supporting Information), thus confirming that the nondynamical correlation is negligible for all the systems investigated.

A final note is deserved. The limitations in accuracy of this method are known in comparison to error-canceling methods like isodesmic reaction ones (i.e., those in which the number and type of different bonds in the species in the right-hand side and left-hand side of the chemical equation match as closely as possible).^43,44^ Nevertheless, the atomization method has been chosen due to its simplicity and immediate applicability to the large number of molecules requiring analysis. Spin–orbit corrections for carbon and oxygen atoms have been included, with respective values of 0.35 and 0.93 kJ mol^–1^.^45^

### Rate Constant Calculation

High-pressure limit rate constants are determined by the conventional transition state theory (TST) within the RRHO approximation, be expressed by
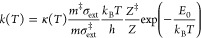
1

Here, κ(*T*) is the transmission coefficient, which accounts for tunneling as well as nonclassical reflection effects using the one-dimensional asymmetric Eckart model. *m*^‡^ and *m* denote the number of enantiomers for the transition state and reactants, respectively, while σ_ext_ and σ refer to the symmetry numbers for external rotation of these entities. The partition functions for the transition state and reactants are denoted by *Z*^‡^ and *Z*, respectively. Furthermore, the constants *h*, *T*, and *k*_B_ represent Planck’s constant, temperature, and Boltzmann’s constant, respectively. The term *E*_0_ represents the barrier height, including the zero-point energy. To model their temperature dependence, the rate constants at different temperatures have been fitted to the Arrhenius equation
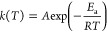
2

The TST calculations have been performed using the MESS code by Georgievskii et al.^46^ A concluding remark on the repercussions of employing the RRHO model is noteworthy. In reactions involving low transitional frequencies, such as those associated with loose transition states, the impact of anharmonicity on rate constants can be substantial.^47,48^ Although the accuracy of rate predictions is primarily influenced by the higher-level theoretical estimates of stationary point energies, the incorporation of anharmonicities, facilitated through approaches like hindered rotor analyses, significantly contributes to diminishing uncertainty in rate predictions. This influence exhibits a linear relationship with rate predictions. Additionally, when employing one-dimensional hindered rotor treatments, determining the optimal symmetry correction factor may not always be straightforward. The level of uncertainty in rate constant calculations is predicted to rise, particularly when dealing with reactive systems featuring multiple coupled internal rotors, especially those incorporating hydrogen bonding interactions. At elevated temperatures, anharmonic effects can significantly impact the estimation of rovibrational density of states, consequently influencing rate constant predictions.^49^ Moreover, reactions involving larger molecules with numerous coupled internal degrees of freedom are expected to exhibit larger uncertainties. Also, it is noted that recrossing and associated nonequilibrium effects may play a significant role in reactions involving large-size systems. This becomes particularly relevant when the dynamics of the environment exhibit slow behavior over the time scale during which the transition state is traversed. In such cases, a reactant-like environment could exert influence, potentially redirecting the system back toward reactants even after crossing the transition state.^50^

### Kinetic Modeling

A kinetic model is built upon the chemical–physical information obtained from thermodynamics and kinetics calculations. This includes providing Arrhenius pre-exponent (*A*) and activation energy (*E*_a_) for each reaction, as well as information on the molar heat capacity at constant pressure, molar enthalpy of formation, and molar entropy of each chemical species. The incorporation of these thermodynamic properties into the kinetic model has been done by means of the THERM code,^51^ and a comprehensive description can be found in the Supporting Information.

Specifically, in this work, two different kinetic models are built to assess the competition between initial competitive pathways. The first kinetic model is composed of the following reactions
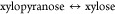
R1
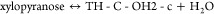
R2
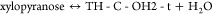
R3
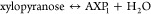
R4
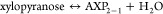
R5
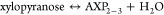
R6
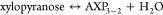
R7
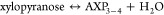
R8
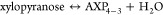
R9
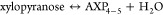
R10

**Figure 1:**
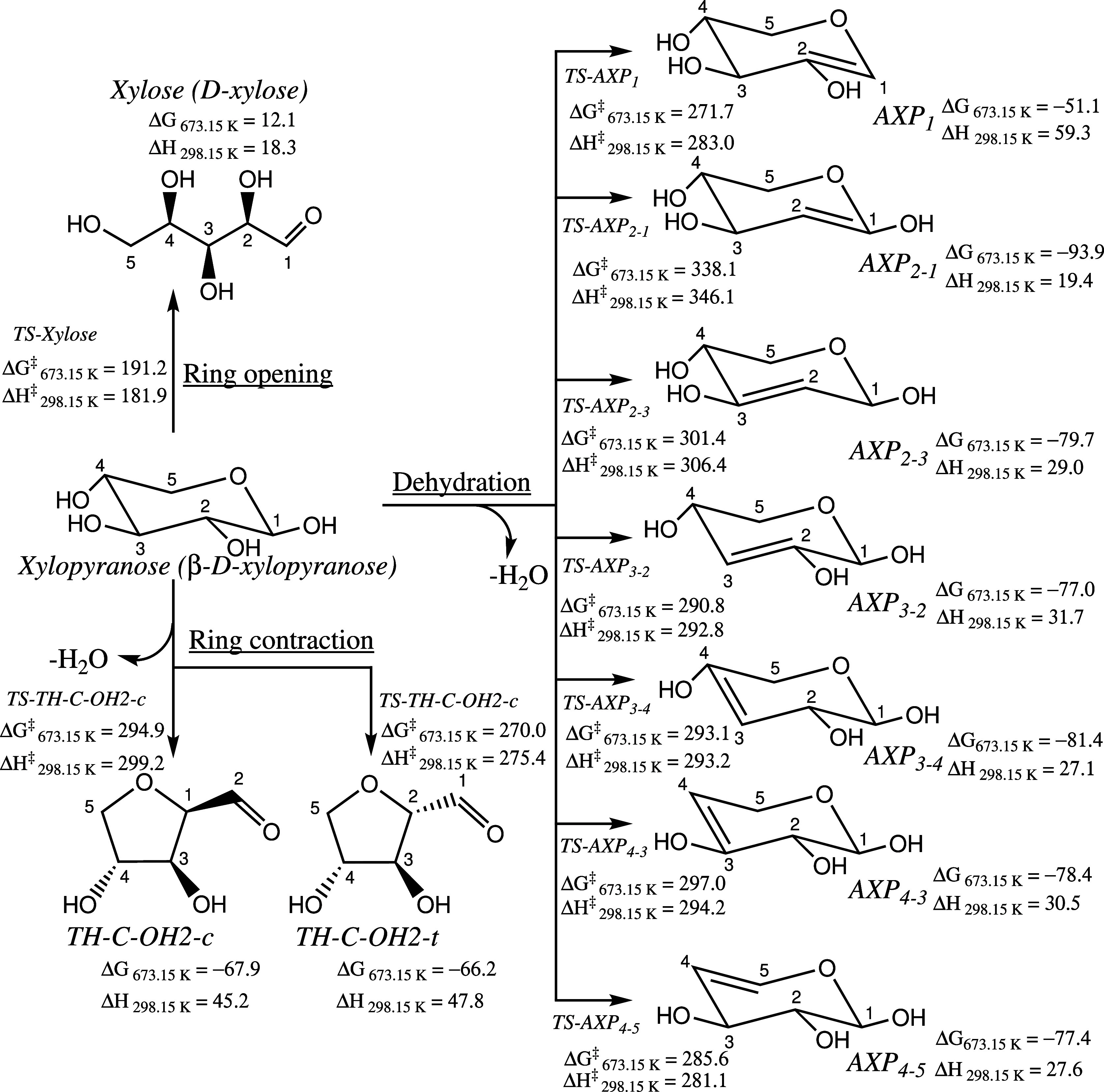
Initial steps of xylopyranose thermal degradation: ring opening, ring contraction, and dehydration. Enthalpies and free energies are in kJ mol^–1^.

These reactions specifically describe the initial stage of thermal decomposition observed in xylopyranose. The second kinetic model is composed of the following reactions
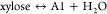
R11
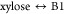
R12
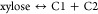
R13
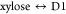
R14which are the series of reactions that xylose can undergo in the subsequent thermal decomposition steps. The significance of this naming convention and the justification for choosing these particular reaction sets will be clarified in the forthcoming discussion within the Results section. For the sake of clarity, the readers can refer to Figure 1. All reactions are reversible, and hence, the equilibrium rate constants are computed using the thermodynamic properties in conjunction with the unidirectional rate constants.

### Reactor Model

To model the thermal decomposition of xylose, the present study employs Cantera,^52^ an open-source chemical kinetic numerical solver. To perform a kinetic simulation using Cantera, a kinetic model must be provided, and the reactor physics must be declared. Zero-dimensional (0-D) simulations are performed to model the time evolution of xylopyranose in a constant-pressure reactor at a set of operating conditions. Two types of simulation are performed. In the first one, the starting temperature is set at 673.15, 773.15, and 1073.15 K, and the kinetic model evolves the species mole fractions as a function of time.

In the second, the evolution of the mixture as the temperature of the reactor increases at a fixed heating rate of 30 K min^–1^ is modeled. This simulation is a representation of a thermogravimetric analysis (TGA) experiment which is a powerful technique for examination of the thermal degradation behavior of a sample. Initial conditions of the reactor are set to 1 atm and 100% mass fraction of xylopyranose. All the species are in the gas phase.

## Results and Discussion

### Molecular Thermodynamics

#### Thermodynamics of Initial Decomposition Pathways of Xylopyranose

β-d-Xylopyranose (**xylopyranose** hereafter) can undergo three reaction pathways, as already recognized in the literature,^16,17^ namely, ring-opening, ring-contraction, and water elimination (dehydration) reactions. Figure 1 illustrates these reactions, with the energies summarized in Table 1.

**Table 1:** Relative Enthalpies and Free Energies at 0, 298.15, and 673.15 K at the DLPNO-CCSD(T)-F12/cc-pVTZ-F12//M06-2X/6-311++G(d,p) Level of Theory for the Species Involved in Xylopyranose Initial Thermal Decomposition Steps^a^

	Δ*H*_0K_	Δ*H*_298.15K_	Δ*G*_298.15K_	Δ*G*_673.15K_	Δ*G*_673.15K_^b^
xylopyranose	0.0	0.0	0.0	0.0	0.0
TS-xylose	183.8	181.9	185.5	191.2	162.0
xylose	18.2	18.3	15.5	12.1	–10.2
TS-TH-C-OH2-c	299.3	299.2	297.1	294.9	261.3
TS-TH-C-OH2-t	274.7	275.4	273.1	270.0	242.6
TH-C-OH2-c + H_2_O	39.2	45.2	–5.0	–67.9	–52.6
TH-C-OH2-t + H_2_O	41.7	47.8	–2.8	–66.2	–58.3
TS-AXP_1_	281.9	283.0	278.2	271.7	250.5
TS-AXP_2–1_	345.6	346.1	342.8	338.1	299.7
TS-AXP_2–3_	306.4	306.4	304.3	301.4	276.9
TS-AXP_3–2_	293.0	292.8	292.1	290.8	278.0
TS-AXP_3–4_	292.9	293.2	293.5	293.1	274.9
TS-AXP_4–3_	294.5	294.2	295.8	297.0	280.8
TS-AXP_4–5_	281.6	281.1	283.3	285.6	269.7
AXP_1_ + H_2_O	52.7	59.3	10.8	–51.1	–41.9
AXP_2–1_ + H_2_O	12.6	19.4	–30.4	–93.9	–69.3
AXP_2–3_ + H_2_O	23.1	29.0	–18.9	–79.7	–59.2
AXP_3–2_ + H_2_O	25.8	31.7	–16.1	–77.0	–53.1
AXP_3–4_ + H_2_O	21.1	27.1	–20.6	–81.4	–64.1
AXP_4–3_ + H_2_O	24.2	30.5	–17.4	–78.4	–56.9
AXP_4–5_ + H_2_O	21.7	27.6	–18.6	–77.4	–53.6

a Pressure is set to 1 atm. Energies in kJ mol^–1^.

b B3LYP-D3/6-311G(d,p) level of theory. Values from ref (17).

The ring-opening reaction of **xylopyranose** leads to the formation of acyclic d-xylose (**xylose** hereafter).

The ring-contraction reactions occur in two ways through **TS-TH-C-OH2-c** and **TS-TH-C-OH2-t**, resulting in the formation of five-membered intermediates, **TH-C-OH2-c** and **TH-C-OH2-t**, i.e., dihydroxytetrahydrofuran-2-carbaldehyde. In the first of the two, the C2–C3 bond breaks while simultaneously forming the C1–C3 bond. In the second one, the C2–O bond forms as the C1–O bond cleavages. Regarding the dehydration reactions, xylopyranose can dehydrate at four sites (1–4), leading to seven possible anhydroxylopyranose (AXP) products: **AXP**_**1**_, **AXP**_**2–1**_, **AXP**_**2–3**_, **AXP**_**3–2**_, **AXP**_**3–4**_, **AXP**_**4–3**_, and **AXP**_**4–5**_. Examination of Table 1 reveals that the ring-opening reaction exhibits the lowest barrier height, namely, 183.8 kJ mol^–1^. Finite temperature effects are observed to slightly decrease the enthalpic barrier by approximately 2 kJ mol^–1^ at 298.15 K. However, due to entropic effects, the barrier is increased to 191.2 kJ mol^–1^ at 673.15 K. Notably, among the initial decomposition steps, the formation of **xylose** product stands out as the sole endergonic process, with a calculated free energy of reaction of 12.1 kJ mol^–1^. The values presented in the final column of Table 1 are taken from Hu et al.^17^ Not surprisingly,^53^ the barriers are consistently underestimated, reaching discrepancies of up to 30.4 kJ mol^–1^. Additionally, it is noteworthy that the ring-opening process is identified as exergonic, while in this work is determined to be endergonic.

Table 2 presents an in-depth analysis of the forward and reverse barriers associated with the initial processes. A striking observation is that the enthalpic reverse barriers consistently exhibit lower values compared to their direct counterparts, both at 0 and 298.15 K. However, this trend is reversed at 673.15 K, considering the influence of entropic effects on free energies. From an entropic perspective, bimolecular reactions are less favored, resulting in increased free energy barriers. It is worth noting that the ring-opening reaction stands as the only exception, as it follows a unimolecular pathway in both directions.

**Table 2:** Barrier Heights at 0, 298.15, and 673.15 K for the Single-Step Reactions of Xylopyranose Initial Thermal Decomposition^a^

	Δ*H*_0K_^‡^	Δ*H*_298.15K_^‡^	Δ*G*_298.15K_^‡^	Δ*G*_673.15K_^‡^
Forward/reverse				
xylopyranose ↔ xylose	183.8/165.7	181.9/163.7	185.5/170.0	191.2/179.1
xylopyranose ↔ TH-C-OH2-c + H_2_O	299.3/260.1	299.2/254.0	297.1/302.1	294.9/362.8
xylopyranose ↔ TH-C-OH2-T + H_2_O	274.7/233.0	275.4/227.6	273.1/275.9	270.0/336.2
xylopyranose ↔ AXP_1_ + H_2_O	281.9/229.2	283.0/223.7	278.2/267.4	271.7/322.7
xylopyranose ↔ AXP_2–1_ + H_2_O	345.6/333.0	346.1/326.8	342.8/373.3	338.1/432.0
xylopyranose ↔ AXP_2–3_ + H_2_O	306.4/283.3	306.4/277.4	304.3/323.2	301.4/381.0
xylopyranose ↔ AXP_3–2_ + H_2_O	293.0/267.2	292.8/261.0	292.1/308.2	290.8/367.7
xylopyranose ↔ AXP_3–4_ + H_2_O	292.9/271.8	293.2/266.0	293.5/314.1	293.1/374.5
xylopyranose ↔ AXP_4–3_ + H_2_O	294.5/270.3	294.2/263.8	295.8/313.2	297.0/375.4
xylopyranose ↔ AXP_4–5_ + H_2_O	281.6/259.9	281.1/253.5	283.3/301.9	285.6/363.0

a Computed at the DLPNO-CCSD(T)-F12/cc-pVTZ-F12//M06-2X/6-311++G(d,p) level of theory. Energies in kJ mol^–1^.

#### Thermodynamics of Xylose Decomposition Pathways

**Figure 2:**
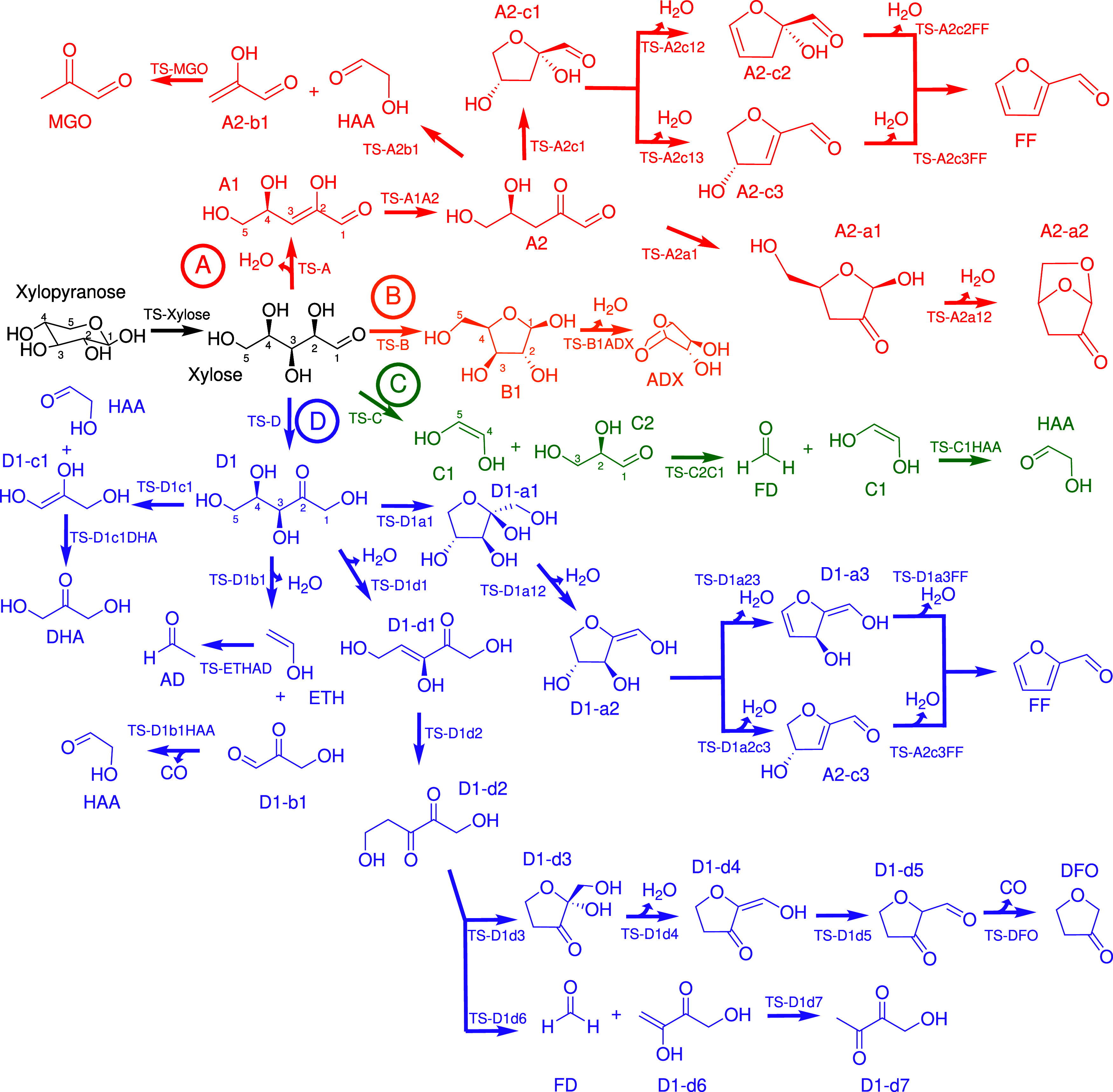
Decomposition pathways of β-d-xylopyranose. Initial pathway is the ring opening, depicted in black. Subsequent dehydration processes are highlighted in red (A), cyclization in orange (B), C–C bond fission in green (C), and isomerization in purple (D).

**Figure 3:**
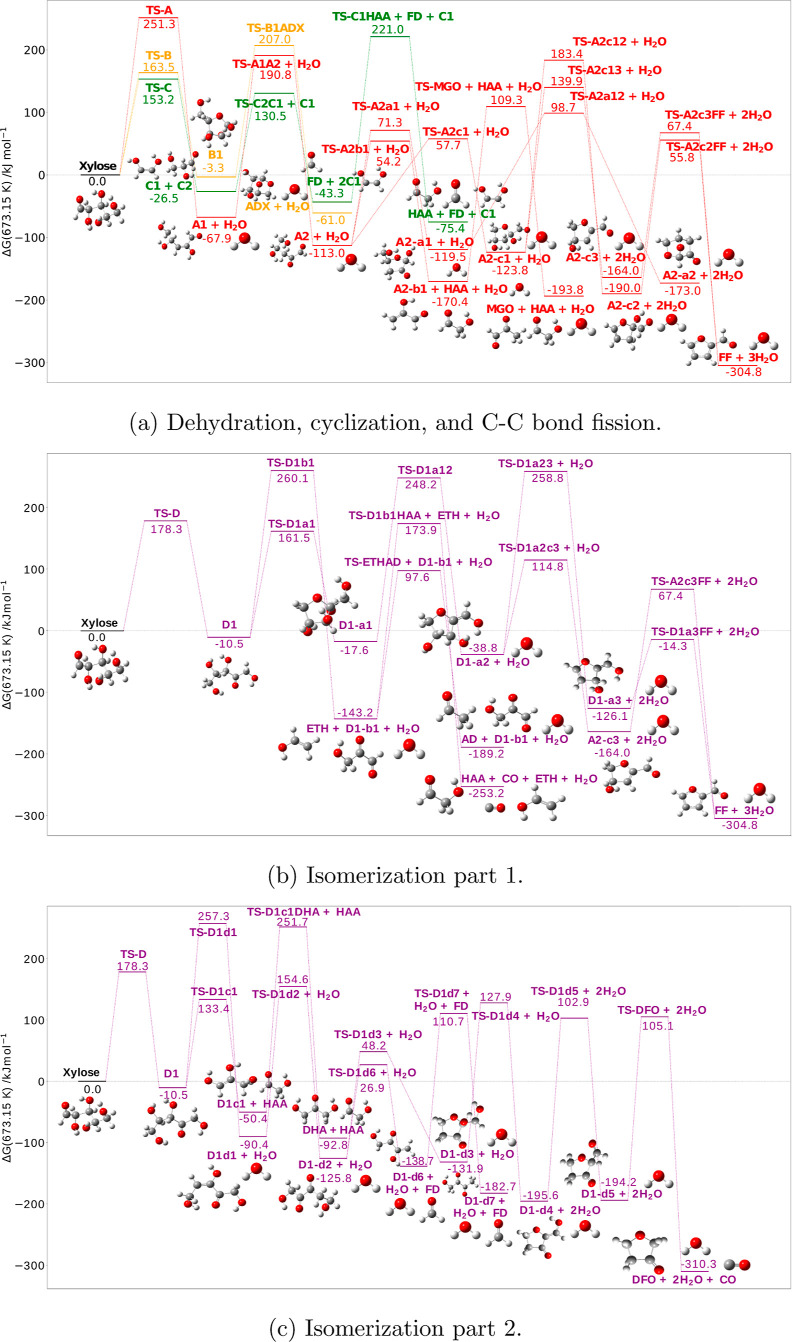
Relative free energies at 673.15 K of xylose thermal decomposition pathways. Panel (a) dehydration in red, cyclization in orange, and C–C bond fission in green. Panels (b,c) isomerization in purple. Δ*G* at the DLPNO-CCSD(T)-F12/cc-pVTZ-F12//M06-2X/6-311++G(d,p) level of theory.

The upcoming kinetic analysis section will demonstrate that the ring-opening pathway is the dominant reaction channel. Consequently, electronic structure calculations were exclusively performed on the reactive pathways associated with the ring-opening reaction. **Xylose** exhibits four distinct types of reactions, depicted in Figure 2: dehydration [red (A)], cyclization [orange (B)], C–C bond fission [green (C)], and isomerization [purple (D)]. The corresponding free energy profiles at 673.15 K are compiled and visualized in Figure 3a–c. This temperature has been chosen as it mirrors the typical operating temperature during fast pyrolysis.

##### Dehydration

Based on the findings presented in Figures 2 and 3a, open-chain d-xylose dehydrates to a trihydroxypentenal (**A1**) intermediate through a free energy barrier of 251.3 kJ mol^–1^. Trihydroxypentenal undergoes tautomerization to its ketoisomer dihydroxyoxopentanal (**A2**) (Δ*G*_673.15K_^‡^ = 258.7 kJ mol^–1^). Subsequently, it undergoes decomposition through three distinct pathways, through transition states **TS-A2a1** (Δ*G*_673.15K_^‡^ = 184.3 kJ mol^–1^), **TS-A2b1** (Δ*G*_673.15K_^‡^ = 167.3 kJ mol^–1^), and **TS-A2c1** (Δ*G*_673.15K_^‡^ = 170.8 kJ mol^–1^), respectively. Within the first path, dihydroxyoxopentanal undergoes a hemiacetal reaction between the carbonyl group at the C1 position and the hydroxyl group at the C4 position, resulting in the formation of a five-membered furanone-like intermediate (**A2-a1**). This intermediate then proceeds to form dioxabicycloheptanone (**A2-a2**) through an acetal reaction (Δ*G*_673.15K_^‡^ = 218.2 kJ mol^–1^). The second path involves the retro-aldol process, which breaks the C3–C4 bond of dihydroxyoxopentanal, resulting in the formation of glycolaldehyde (**HAA**) and hydroxyacrylaldehyde (**A2-b1**). Last, hydroxyacrylaldehyde undergoes tautomerization (Δ*G*_673.15K_^‡^ = 279.6 kJ mol^–1^), leading to the formation of methylglyoxal (**MGO**). In the third pathway, a hemiacetal reaction occurs between the carbonyl group at the C2 position and the hydroxyl group at the C5 position, resulting in the formation of a five-membered tetrahydrofurancarbaldehyde-like intermediate (**A2-c1**). Sequential dehydration reactions take place through **TS-A2c12** and **TS-A2c2FF** (Δ*G*_673.15K_^‡^ = 307.2 and 245.8 kJ mol^–1^) and **TS-A2c13** and **TS-A2c3FF** (Δ*G*_673.15K_^‡^ = 263.7 and 231.4 kJ mol^–1^), leading to the generation of furfural (**FF**). Out of all the dehydration products, furfural exhibits the highest exoergonicity, with a reaction free energy of −304.8 kJ mol^–1^. In comparison, methylglyoxal possesses a reaction free energy of −93.8 kJ mol^–1^, and dioxabicycloheptanone has a reaction free energy of −173.0 kJ mol^–1^.

##### Cyclization

**Xylose** can undergo either hemiacetal or acetal reactions during cyclization, with the hemiacetal reaction being more favorable.^17^ The most favorable pathway is the formation of five-membered intermediate xylofuranose (**B1**), through transition state **TS-B** (Δ*G*_673.15K_^‡^ = 163.5 kJ mol^–1^). Subsequently, xylofuranose undergoes an acetal reaction to form the 1,5-acetal ring, resulting in the formation of anhydro-d-xylopyranose (**ADX**), which lies at −61.0 kJ mol^–1^. This step has the highest free energy barrier of this pathway (Δ*G*_673.15K_^‡^ = 210.2 kJ mol^–1^).

##### Carbon–Carbon Bond Scission

According to Hu et al.,^17^ the degradation of **xylose** involves two primary mechanisms: retro-aldol reaction and cyclic Grob fragmentation, both with relatively low activation energies. Among these mechanisms, the retro-aldol reaction is found to be the most favorable. Hence, the study focuses solely on this reaction. Initially, **xylose** undergoes a retro-aldol reaction, leading to the formation of intermediate glyceraldehyde (**C2**) and the enol isomer of glycolaldehyde (**C1**) through the cleavage of the C2–C3 bond (Δ*G*_673.15K_^‡^ = 153.2 kJ mol^–1^). Subsequently, glyceraldehyde undergoes another retro-aldol reaction to generate the enol isomer (Δ*G*_673.15K_^‡^ = 157.0 kJ mol^–1^). Last, the enol undergoes an enol–keto tautomerization process, resulting in the formation of glycolaldehyde (**HAA**). This particular step has the higher free energy barrier on the sequence for glycolaldehyde formation, so will impact its rate of formation most significantly (Δ*G*_673.15K_^‡^ = 264.2 kJ mol^–1^).

##### Isomerization

**Xylose** can undergo an isomerization reaction. As an open-chain aldose, it readily undergoes isomerization to its ketone isomer, d-xylulose (**D1**), with an activation free energy of 178.3 kJ mol^–1^. Similar to **xylose**, d-xylulose undergoes decomposition through cyclization, C–C bond cleavage, and dehydration reactions. Through transition state **TS-D1a1** (Δ*G*_673.15K_^‡^ = 171.9 kJ mol^–1^), d-xylulose forms a five-membered tetrahydrofuran-like intermediate (**D1-a1**) through a hemiacetal reaction. Subsequently, it undergoes dehydration at the 2-OH&1-H sites, generating **D1-a2** (Δ*G*_673.15K_^‡^ = 265.8 kJ mol^–1^). This intermediate further produces furfural (**FF**) through successive dehydration reactions. The dehydration at the 2-OH&1-H sites (**D1-a1** to **D1-a2**) serves as the rate step for furfural formation. In contrast, the dehydration at the 4-OH&5-H sites (**D1-a2** to **D1-a3**) may limit the rate of furfural formation in the concurrent path, as the activation free energy of Δ*G*_673.15K_^‡^ = 297.6 kJ mol^–1^ is 32 kJ mol^–1^ higher. Moreover, d-xylulose undergoes cyclic Grob fragmentation at the 5-OH&3-H sites, generating ethenol (**ETH**) and 3-hydroxy-2-oxopropanal (**D1-b1**) with an activation free energy of 270.5 kJ mol^–1^. Ethenol then tautomerizes into acetaldehyde (**AD**), with an activation free energy of 240.9 kJ mol^–1^ above reactant. 3-Hydroxy-2-oxopropanal undergoes decarbonylation to form glycolaldehyde (**HAA**) (Δ*G*_673.15K_^‡^ = 317.2 kJ mol^–1^). The C3–C4 bond of d-xylulose undergoes cleavage via a retro-aldol reaction, resulting in the formation of glycolaldehyde (**HAA**) and **D1-c1** (Δ*G*_673.15K_^‡^ = 143.8 kJ mol^–1^). It then generates 1,3-dihydroxyacetone (**DHA**) through tautomerization. The reaction step with the highest Δ*G*^‡^ barrier for 1,3-dihydroxyacetone formation is the tautomerization of **D1-c1** into itself, with a 302.1 kJ mol^–1^ free energy barrier. In addition to the previously mentioned pathways, the dehydration at 4-OH&3-H sites (Δ*G*_673.15K_^‡^ = 267.8 kJ mol^–1^) and following tautomerization (Δ*G*_673.15K_^‡^ = 244.9 kJ mol^–1^) result in the formation of dihydroxypentanedione (**D1-d2**) which decomposes through TSs **TS-D1d3** and **TS-D1d6**, with activation energies of 174.1 and 152.7 kJ mol^–1^, respectively. In the former case, dihydroxypentanedione undergoes a hemiacetal reaction, forming the five-membered furanone-like intermediate (**D1-d3**). Subsequently, it undergoes successive dehydration reactions at the 2-OH&1-H sites, followed by tautomerization and decarbonylation, with activation energies, in order, of 259.8, 298.5, and 299.3 kJ mol^–1^ leading to the formation of dihydrofuran-3(2*H*)-one (**DFO**). The highest free energy barrier reaction step in this path is the tautomerization of dehydrated furanone-like intermediate **D1-d4**. In the latter case, dihydroxypentanedione undergoes a retro-aldol reaction, resulting in the cleavage of the C5–C6 bond and the formation of formaldehyde (**FD**) and dihydroxybutenone (**D1-d6**), with an activation energy of 152.7 kJ mol^–1^. Last, dihydroxybutenone tautomerizes into hydroxybutanedione (**D1-d7**) overcoming a free energy barrier of 249.4 kJ mol^–1^. Formation of dihydrofuran-3(2*H*)-one is the most exergonic process with a relative free energy of −310.3 kJ mol^–1^, followed by furfural −304.8 kJ mol^–1^.

Detailed forward and reverse reaction barriers at 0 K, standard, and operative conditions are reported in Table 3. Enthalpies of formation, entropies, and heat capacities at constant pressure at different temperatures are reported in Table 4. As described in the Computational Methodology section, enthalpies of formation were determined by using the enthalpies of atomization. To further validate our findings, in Table 4 are included the enthalpy of formation values from ATcT for commonly known species: H_2_O (−241.80 kJ mol^–1^), ethenol (ETH) (−123.72 kJ mol^–1^), acetaldehyde (AD) (−165.55 kJ mol^–1^), CO (−110.52 kJ mol^–1^), formaldehyde (FD) (−109.23 kJ mol^–1^), and hydroxyacetaldehyde (−317.50 kJ mol^–1^). The errors in our methodology are quantified by a mean unsigned error (MUE) of 6.35 kJ mol^–1^ and a root-mean-square deviation (RMSD) of 7.50 kJ mol^–1^ (considering the enthalpies of formation without SO corrections). Evaluating the accuracy achieved by incorporating SO corrections in the calculation is enlightening. SO-corrected enthalpies of formation are reported in square brackets in Table 4. Using the new enthalpies as a reference, the error on the uncorrected ones is quantified by a MUE of 4.50 kJ mol^–1^ and a RMSD of 4.74 kJ mol^–1^. Moreover, the MUE and RMSD with respect of ATcT values are now lowered down to 5.10 and 6.19 kJ mol^–1^, respectively. This implies that higher accuracy is reached effortlessly, indicating the recommendation to consistently include spin–orbit corrections. It should be noted that this paper does not focus on benchmarking the enthalpy of formation; rather, the provided numbers serve as reference points to gauge the effectiveness of the employed approach. For greater accuracy, methods that incorporate error cancellation, such as the isodesmic approach, or the new CBH-ANL approach by Elliott et al.,^54^ should be considered. The latter, in particular, is based on the original idea of Raghavachari and Sengupta,^55^ which demonstrated the feasibility of achieving precise thermochemical results through relatively fast electronic structure calculations using the connectivity-based hierarchy (CBH) method. This approach is based on error cancellation by strategically selecting a reference reaction. As far as the authors know, this is the first instance in the literature where thermochemical parameters for the pyrolytic reaction system of xylopyranose have been directly derived from first principles. A final remark is deserved. The role of free energies, as opposed to enthalpies, has been emphasized in this section, with a particular focus on the significance of the Gibbs energy of activation in the examination of mechanisms and kinetics in chemical reactions. This emphasis is attributed to the fact that it accounts for both enthalpic and entropic corrections associated with electronic and zero-point energy components. It is essential to recognize that, especially at low temperatures or energies, certain reaction pathways may initially appear less favorable primarily due to their enthalpy contributions. However, as temperatures and energies increase, entropic factors begin to exert their influence, potentially making these pathways more dominant. Therefore, the prediction and exploration of activation free energy become of paramount importance when the primary goal is to gain insights into reaction kinetics and to discern the prevailing mechanistic pathways. This approach offers a more comprehensive and enlightening perspective in the field of chemical reaction studies.

**Table 3:** Activation Enthalpies and Free Energies at 0, 298.15, and 673.15 K at DLPNO-CCSD(T)-F12/cc-pVTZ-F12//M06-2X/6-311++G(d,p) for the Single-Step Reactions of d-Xylose Thermal Decomposition Pathways^a^

reaction	Δ*H*_0K_^‡^	Δ*H*_298.15K_^‡^	Δ*G*_298.15K_^‡^	Δ*G*_673.15K_^‡^
Forward/reverse				
xylose ↔ A1 + H_2_O	263.8/213.5	265.4/206.5	259.4/256.1	251.3/319.2
A1 ↔ A2	258.9/310.2	258.1/310.2	257.8/307.2	258.7/303.8
A2 ↔ A2-a1	172.9/195.0	170.4/195.3	175.9/193.1	184.3/190.8
A2-a1 ↔ A2-a2 + H_2_O	215.3/178.8	214.9/173.8	216.3/217.3	218.2/271.8
A2 ↔ A2-b1 + HAA	164.7/101.9	163.4/96.3	164.7/152.6	167.3/224.6
A2-b1 ↔ MGO	282.6/297.2	282.4/296.3	280.8/299.2	279.6/303.1
A2 ↔ A2-c1	158.6/183.1	156.5/183.5	162.4/182.5	170.8/181.5
A2-c1 ↔ A2-c2 + H_2_O	312.6/275.1	314.0/269.6	311.6/315.7	307.2/373.4
A2-c2 ↔ FF + H_2_O	250.4/265.0	252.1/260.1	249.8/304.9	245.8/360.6
A2-c1 ↔ A2-c3 + H_2_O	268.7/201.2	269.6/194.5	267.1/242.6	263.7/303.9
A2-c3 ↔ FF + H_2_O	234.0/278.6	234.8/273.5	233.6/317.3	231.4/372.2
xylose ↔ B1	157.3/163.5	156.3/163.1	159.1/164.4	163.5/166.7
B1 ↔ ADX + H_2_O	208.5/178.9	208.2/174.6	208.9/216.0	210.2/267.9
xylose ↔ C1 + C2	159.0/43.6	159.8/38.1	156.8/100.6	153.2/179.7
C2 ↔ FD + C1	147.1/61.5	146.4/55.7	151.1/107.9	157.0/173.8
C1 ↔ HAA	261.0/294.0	260.6/293.8	261.9/295.0	264.2/296.4
xylose ↔ D1	179.6/176.2	178.6/173.4	177.9/179.4	178.3/188.7
D1 ↔ D1-a1	155.1/182.6	153.0/183.0	161.0/181.0	171.9/179.1
D1-a1 ↔ D1-a2 + H_2_O	277.5/191.0	279.3/185.6	273.5/230.5	265.8/287.1
D1-a2 ↔ D1-a3 + H_2_O	305.1/292.5	306.3/287.7	303.0/330.8	297.6/384.8
D1-a3 ↔ FF + H_2_O	104.8/182.3	103.8/175.0	107.0/225.9	111.8/290.5
D1-a2 ↔ A2-c3 + H_2_O	148.7/169.0	147.9/161.8	150.2/213.2	153.6/278.8
D1 ↔ D1-b1 + ETH + H_2_O	268.5/177.5	267.9/166.1	269.1/271.1	270.5/403.3
ETH ↔ AD	238.0/280.4	237.3/279.2	238.5/282.4	240.9/286.8
D1-b1 ↔ HAA + CO	330.7/346.5	331.8/342.9	325.4/380.2	317.2/427.2
D1 ↔ D1-c1 + HAA	135.4/54.0	134.4/48.8	138.4/108.3	143.8/183.7
D1-c1 ↔ DHA	306.0/345.0	306.2/345.6	304.0/345.2	302.1/344.6
D1 ↔ D1-d1 + H_2_O	269.3/248.7	269.3/242.2	268.8/288.6	267.8/347.7
D1-d1 ↔ D1-d2	246.6/277.0	246.1/276.9	245.1/278.5	244.9/280.4
D1-d2 ↔ D1-d3	152.7/178.1	150.2/177.9	160.4/178.7	174.1/180.2
D1-d3 ↔ D1-d4 + H_2_O	267.8/228.1	270.5/223.9	266.4/268.3	259.8/323.5
D1-d4 ↔ D1-d5	298.8/298.1	298.3/298.1	298.0/297.5	298.5/297.1
D1-d5 ↔ DFO + CO	308.9/325.2	310.2/321.9	305.6/363.6	299.3/415.4
D1-d2 ↔ FD + D1-d6	143.9/51.8	142.4/45.2	146.8/98.1	152.7/165.6
D1-d6 ↔ D1-d7	250.4/287.4	250.0/286.7	249.2/289.6	249.4/293.3

a Pressure is set at 1 atm. Energies in kJ mol^–1^.

**Table 4:** Enthalpies of Formation (Δ_f_*H*°) and Entropies (*S°*) of Molecules at 298.15 K and 1 atm and Heat Capacities (*C*_p_) at Different Temperatures^a^

	Δ_f_*H*°	*S*°	*C*_p_ (300 K)	*C*_p_ (400 K)	*C*_p_ (500 K)	*C*_p_ (600 K)	*C*_p_ (800 K)	*C*_p_ (1000 K)	*C*_p_ (1500 K)	chemical formula
xylopyranose	–881.85 [−888.28]	400.15	168.60	212.33	250.57	281.98	328.47	360.92	410.09	C_5_H_10_O_5_
xylose	–863.55 [−869.99]	409.57	171.62	215.28	252.83	283.55	329.18	361.29	410.35	C_5_H_10_O_5_
AXP_1_	–579.18 [−584.68]	374.16	145.63	183.44	215.83	242.12	280.73	307.53	348.08	C_5_H_8_O_4_
AXP_2–1_	–619.09 [−624.60]	378.62	145.71	183.62	216.06	242.34	280.86	307.59	348.05	C_5_H_8_O_4_
AXP_2–3_	–609.46 [−614.96]	372.30	142.96	181.49	214.35	240.96	279.99	307.05	347.90	C_5_H_8_O_4_
AXP_3–2_	–606.71 [−612.21]	372.20	143.30	181.82	214.64	241.21	280.16	307.17	347.94	C_5_H_8_O_4_
AXP_3–4_	–611.31 [−616.81]	371.74	144.00	182.39	215.10	241.58	280.41	307.34	348.00	C_5_H_8_O_4_
AXP_4–3_	–608.00 [−613.50]	372.20	144.38	182.17	214.65	241.12	280.16	307.30	348.21	C_5_H_8_O_4_
AXP_4–5_	–610.90 [−616.40]	366.35	141.94	181.27	214.60	241.40	280.37	307.26	347.86	C_5_H_8_O_4_
TH-C-OH2-c	–593.26 [−598.76]	379.99	141.11	178.95	212.07	239.24	279.45	307.41	349.06	C_5_H_8_O_4_
TH-C-OH2-t	–590.67 [−596.17]	381.12	142.38	180.11	213.09	240.09	279.98	307.70	349.10	C_5_H_8_O_4_
H_2_O	–243.39 [−244.33]	188.58	33.49	34.14	35.09	36.14	38.31	40.56	45.79	H_2_O
	(−241.80)									
A1	–561.33 [−566.83]	407.14	156.54	191.10	220.99	245.64	282.60	308.75	348.74	C_5_H_8_O_4_
A2	–613.36 [−618.86]	398.08	150.92	185.97	216.65	242.12	280.54	307.77	348.99	C_5_H_8_O_4_
A2-a1	–638.29 [−643.79]	372.11	138.94	177.85	211.35	238.63	278.87	306.83	348.61	C_5_H_8_O_4_
A2-a2	–353.77 [−358.34]	324.63	102.53	137.73	168.03	192.47	227.99	252.14	286.96	C_5_H_6_O_3_
A2-b1	–239.54 [−242.46]	297.75	80.64	98.67	113.78	126.12	144.71	158.00	178.41	C_3_H_4_O_2_
A2-c1	–640.37 [−645.87]	374.94	141.34	179.95	213.12	240.11	279.93	307.62	349.07	C_5_H_8_O_4_
A2-c2	–352.54 [−357.11]	349.10	119.23	151.44	178.62	200.53	232.59	254.72	287.45	C_5_H_6_O_3_
A2-c3	–321.83 [−326.40]	356.25	119.05	149.98	176.78	198.72	231.19	253.69	286.94	C_5_H_6_O_3_
B1	–870.30 [−876.73]	404.95	167.49	212.32	251.14	282.70	328.99	361.15	410.02	C_5_H_10_O_5_
C1	–273.58 [−276.15]	285.09	71.62	86.89	99.80	110.21	125.62	136.66	154.38	C_2_H_4_O_2_
C2	–468.24 [−472.10]	344.19	107.31	129.65	149.81	166.81	192.78	211.45	240.27	C_3_H_6_O_3_
D1	–858.35 [−864.78]	432.34	179.13	220.24	256.24	286.00	330.57	362.10	410.53	C_5_H_10_O_5_
D1-a1	–888.39 [−894.82]	398.66	167.16	212.91	251.91	283.39	329.38	361.32	410.01	C_5_H_10_O_5_
D1-a2	–551.37 [−556.87]	379.80	146.00	183.41	215.63	241.90	280.61	307.54	348.22	C_5_H_8_O_4_
D1-a3	–289.31 [−293.87]	347.18	120.49	153.03	180.15	201.76	232.99	254.39	286.41	C_5_H_6_O_3_
D1-b1	–393.44 [−397.30]	335.53	96.73	114.71	130.71	144.24	165.02	179.84	202.08	C_3_H_4_O_3_
D1-c1	–465.91 [−469.77]	334.00	107.56	131.65	152.17	168.90	193.79	211.51	239.37	C_3_H_6_O_3_
D1-d1	–587.81 [−593.31]	401.20	155.69	190.43	220.44	245.17	282.25	308.48	348.61	C_5_H_8_O_4_
D1-d2	–618.65 [−624.15]	409.75	153.28	187.19	217.22	242.34	280.49	307.65	348.88	C_5_H_8_O_4_
D1-d3	–646.38 [−651.88]	378.07	142.23	180.31	213.11	239.87	279.48	307.12	348.63	C_5_H_8_O_4_
D1-d4	–356.41 [−360.98]	352.05	117.40	148.64	175.56	197.60	230.26	252.94	286.50	C_5_H_6_O_3_
D1-d5	–356.18 [−360.74]	351.27	114.60	145.85	173.19	195.78	229.59	253.16	287.56	C_5_H_6_O_3_
D1-d6	–417.52 [−421.73]	353.82	120.65	147.04	169.31	187.50	214.69	234.02	263.77	C_4_H_6_O_3_
D1-d7	–454.25 [−458.46]	365.85	120.52	144.80	166.40	184.66	212.84	233.17	264.16	C_4_H_6_O_3_
DFO	–270.05 [−273.34]	309.03	87.36	114.04	137.85	157.71	187.74	208.97	240.49	C_4_H_6_O_2_
ETH	–119.77 [−121.41]	256.18	56.08	69.14	80.44	89.76	103.98	114.48	131.47	C_2_H_4_O
	(−123.72)									
FF	–117.08 [−120.72]	318.60	91.29	116.89	138.81	156.52	182.29	199.77	224.85	C_5_H_4_O_2_
MGO	–253.47 [−256.39]	312.78	82.58	98.26	112.38	124.53	143.70	157.73	179.02	C_3_H_4_O_2_
DHA	–505.26 [−509.12]	340.30	105.62	128.26	148.65	165.85	192.19	211.14	240.30	C_3_H_6_O_3_
AD	–161.67 [−163.31]	262.97	55.20	66.29	76.99	86.51	102.04	113.79	132.21	C_2_H_4_O
	(−165.55)									
CO	–97.79 [−99.08]	197.40	29.12	29.25	29.60	30.15	31.48	32.71	34.75	CO
	(−110.52)									
ADX	–593.37 [−598.87]	352.68	129.44	171.17	207.15	236.09	277.89	306.35	348.36	C_5_H_4_O_4_
FD	–103.99 [−105.27]	218.48	35.14	38.61	42.84	47.10	54.66	60.66	70.15	CH_2_O
	(−109.23)									
HAA	–306.78 [−309.36]	284.71	67.95	82.06	95.00	106.08	123.33	135.90	155.27	C_2_H_4_O_2_
	(−317.50)									

a In parentheses, values taken from ATcT, while in square brackets, values including spin–orbit corrections. Nomenclature is the same used in Figures 1 and 2. Units are kJ mol^–1^ for Δ_f_*H*° and J mol^–1^ K^–1^ for *S*° and *C*_p_.

### Reaction Kinetics

**Figure 4:**
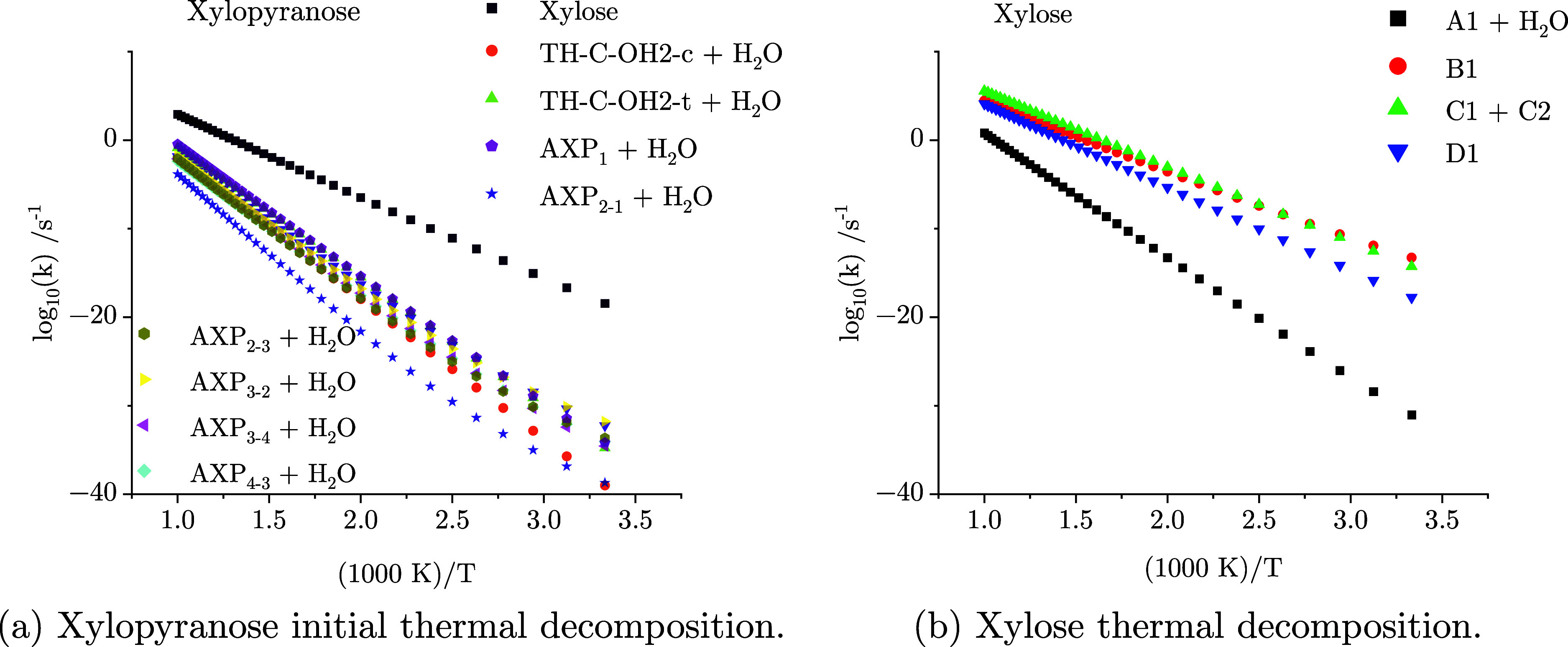
Temperature-dependent rate constants for xylopyranose decomposition [panel (a) xylose ring opening, TH-C-OH2-(c,t) ring contraction, and AXP_*n*_ dehydration] and d-xylose decomposition [panel (b) A1 + H_2_O dehydration, B1 cyclization, C1 + C2 carbon–carbon bond fission, and D1 isomerization].

The determination of temperature-dependent reaction rate constants for the initial decomposition pathways of **xylopyranose** reveals that the ring-opening channel dominates across the entire temperature range of 300–1000 K, as shown in Figure 4a. Figure 4b shows the temperature-dependent rate coefficients of **xylose** decomposition pathways. At very high temperatures, there is a strong competition between all four reaction pathways, with the C–C bond scission leading to **C1 + C2** pathway proceeding at the fastest rate. However, as the temperature decreases, the isomerization channel leading to **D1** and the dehydration pathway leading to **A1 + H**_**2**_**O** become notably slower. Furthermore, as the temperature decreases below 400 K, the cyclization channel leading to **B1** becomes the most reactive pathway. Notably, each reaction rate constant appears to obey an Arrhenius-like temperature dependence.

### Kinetic Model Analysis

In this section, two distinct kinetic models for the thermal decomposition of **xylopyranose** are constructed using the high-pressure rate constants and the previously calculated formation enthalpies. The results are discussed to evaluate the role and competition of parallel reaction pathways.

#### Kinetic Model of Xylopyranose Decomposition

**Figure 5:**
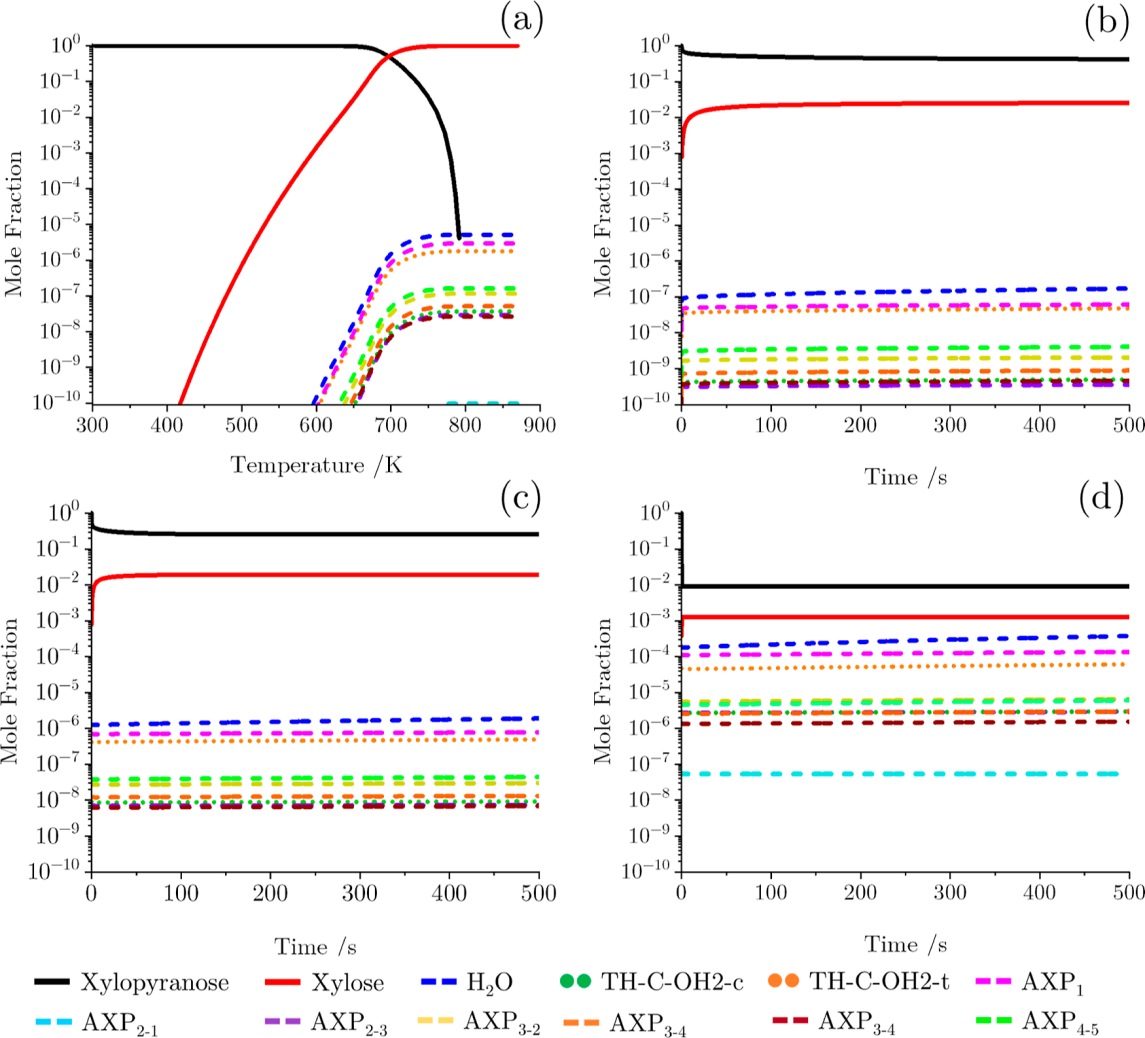
Mole fraction of chemical species produced from the initial competition decomposition reaction pathways of xylopyranose at 1 atm in (a) TGA-like simulation with a heating rate of 20 °C min^–1^. (b–d) Isobaric reactor at 673.15, 773.15, and 1073.15 K, respectively, as a function of time.

A chemical kinetic model, including reactions R1–R10, was constructed for this system to allow for the competition between the initial decomposition steps of **xylopyranose** to be studied using kinetic modeling and is reported in the Supporting Information materials. The Arrhenius pre-exponent (*A*) and activation energy (*E*_a_) for each reaction pathway was obtained by fitting the temperature-dependent rate coefficients to the Arrhenius equation (eq 2) and are presented in Table 5. Figure 5a shows the results of a TGA-like simulation with a heating rate of 20 °C min^–1^ using this kinetic model. From this, one can observe that the thermal decomposition of **xylopyranose** begins at approximately 670 K. This figure indicates that the ring-opening reaction pathway leading to the **xylose** open-chain product is the preferred reaction pathway, as this product is present in large quantities, with the other products present in negligible concentrations. Figure 5b–d shows the time evolution of **xylopyranose** in a fixed temperature isobaric reactor at 673.15, 773.15, and 1073.15 K, respectively. These figures highlight that the ring-opening pathway dominates at all temperatures, with the competition between pathways increasing as the temperature increases, as indicated in Figure 4a. This is as one would expect by analyzing the activation energies in Table 5 whereby the open-chain reaction (181.8 kJ mol^–1^) is approximately 100 kJ mol^–1^ lower than the minimum activation energy observed among the other initial decomposition reaction channels. Based on this conclusion, it is reasonable to neglect the ring-contraction and dehydration channels from further consideration.

**Table 5:** Arrhenius Parameters Obtained through Fitting of Rate Constants in the 300–1000 K Temperature Range for the Initial Thermal Decomposition Pathways of Xylopyranose and Subsequent Pathways Originating from the Open-Chain Form of d-Xylose

reaction	*A*/s^–1^	*E*_a_/kJ mol^–1^	RSS^a^
R1 xylopyranose → xylose	2.53 × 10^12^	181.8	7.30 × 10^–2^
R2 xylopyranose → TH-C-OH2-c + H_2_O	7.08 × 10^13^	305.1	1.80 × 10^–13^
R3 xylopyranose → TH-C-OH2-t + H_2_O	6.42 × 10^13^	280.8	7.90 × 10^–11^
R4 xylopyranose → AXP_1_ + H_2_O	4.60 × 10^14^	289.7	4.70 × 10^–9^
R5 xylopyranose → AXP_2–1_ + H_2_O	2.76 × 10^14^	349.9	9.90 × 10^–16^
R6 xylopyranose → AXP_2–3_ + H_2_O	1.23 × 10^14^	309.8	4.60 × 10^–12^
R7 xylopyranose → AXP_3–2_ + H_2_O	4.58 × 10^13^	295.4	2.60 × 10^–11^
R8 xylopyranose → AXP_3–4_ + H_2_O	2.32 × 10^13^	296.2	3.60 × 10^–12^
R9 xylopyranose → AXP_4–3_ + H_2_O	1.26 × 10^13^	296.5	1.10 × 10^–12^
R10 xylopyranose → AXP_4–5_ + H_2_O	8.83 × 10^12^	283.3	1.60 × 10^–11^
R11 xylose → A1 + H_2_O	1.17 × 10^15^	273.4	7.30 × 10^–8^
R12 xylose → B1	4.17 × 10^12^	156.4	12.4
R13 xylose → C1 + C2	1.76 × 10^14^	166.5	421.5
R14 xylose → D1	4.38 × 10^13^	182.7	6 × 10^–1^

a rss stands for residual sum of squares of the fit.

#### Kinetic Model of Xylose Decomposition

**Figure 6:**
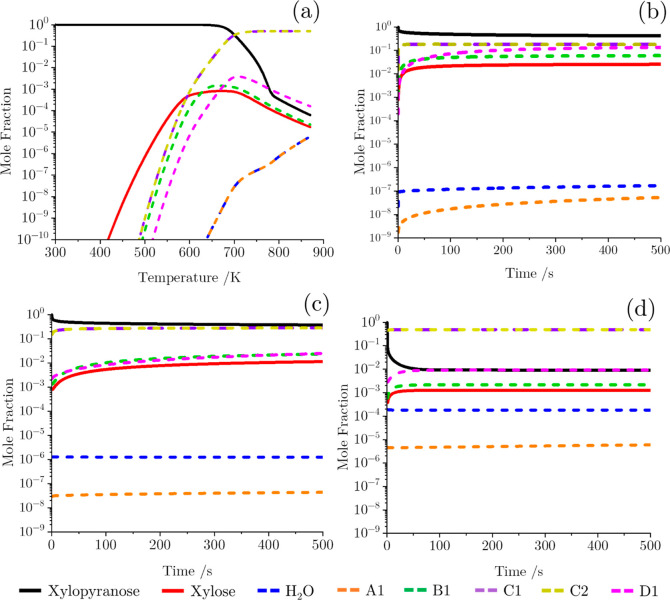
Mole fraction of chemical species produced from the second competition decomposition reaction pathways of xylopyranose at 1 atm in (a) TGA-like simulation with a heating rate of 20 °C min^–1^. (b–d) Isobaric reactor at 673.15, 773.15, and 1073.15 K, respectively, as a function of time.

A second kinetic model, comprising reactions R1, R11–R14, is constructed by fitting the temperature-dependent reaction rate constants of Figure 4b to the Arrhenius equation. This kinetic model now describes the thermal degradation of **xylopyranose** to **xylose** and the subsequent competitive parallel reactions, namely, dehydration (A), cyclization (B), C–C bond fission (C), and isomerization (D), as depicted in Figure 2. The results of the TGA-like simulation, shown in Figure 6a, shows that **xylopyranose** decomposes to **xylose**, as in Figure 5a; however, the **xylose** now breaks down through these new reaction pathways. At low temperatures, 400–600 K, **xylose** decomposes primarily to **C1** and **C2**; however, a sizable quantity of **B1** and **D1** is also produced. As the temperature increases, however, the concentration of **B1** and **D1** decreases. At higher temperatures, 800–900 K, the concentration of **A1** and **H**_**2**_**O** begins to increase as the dehydration of xylose pathway begins to compete with that of the other pathways. This is as expected from analysis of the reaction rate constants only, e.g., Figure 4b. Additionally, it is interesting to note that the concentration of **xylopyranose** remains orders of magnitude higher at high temperatures compared to in Figure 5a. This is likely due to the removal of the **xylopyranose** ring-contraction and dehydration pathways which become competitive at high temperatures, as shown in Figure 4a. Figure 6b–d shows the time evolution of **xylopyranose** in a fixed temperature isobaric reactor at 673.15, 773.15, and 1073.15 K respectively. The results of these simulations further reinforce the conclusions drawn from Figure 6a that the C–C bond fission reaction pathway dominates at all temperatures, producing **C1** and **C2**, with all other products having mole fractions orders of magnitude lower.

This is a demonstration of the method of iteratively evaluating the competition between parallel reaction pathways, using temperature-dependent reaction rate constants and kinetic modeling, and retaining only those that contribute appreciably to the formation of the reaction products.

#### Reaction Flux Analysis

**Figure 7:**
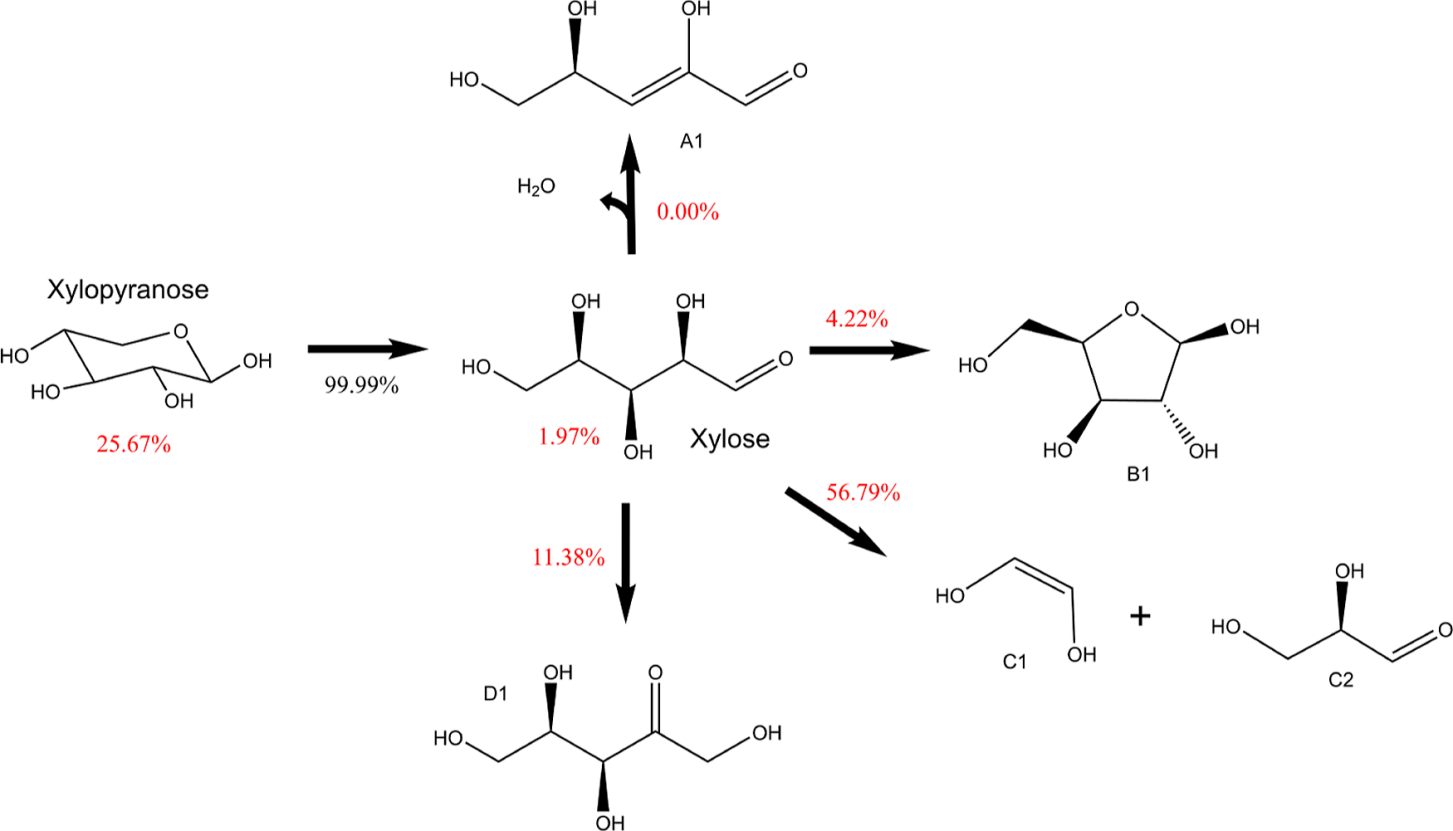
Flux diagram depicting β-d-xylopyranose decomposition at 773.15 K. Red numbers represent branching ratios calculated with the kinetic model, including the second set of competitive pathways, while black number indicates the first competition leading to d-xylose open-chain formation.

Figure 7 illustrates the chemical flux analysis of xylopyranose at 773.15 K and 1 atm, representing a critical set of test conditions explored in our study. The flux analysis offers valuable insights into the pyrolysis of xylopyranose under fast pyrolysis conditions. Our analysis reveals that nearly 99% of xylopyranose undergoes decomposition into the xylose open chain, with only trace amounts of xylopyranose participating in water–loss reactions.

When we incorporate the second competitive reaction pathways into the model (highlighted by the red numbers), a distinct shift in product distribution is observed. Approximately 57% of the xylopyranose is transformed into C1 + C2, followed by about 11% forming xylulose (D1) and 4% yielding xylofuranose (B1). It is worth noting that a substantial portion of xylose (ca. 26%) goes back to xylopyranose due to its thermodynamic stability.

## Conclusions

In summary, the gas-phase pyrolytic reactivity of β-d-xylopyranose, which represents a fundamental structural motif of hemicellulose, has been investigated. A combined quantum chemistry/chemical kinetics analysis has been performed on literature selected reaction pathways, employing a higher level of theory. Electronic energies and finite temperature corrections were recomputed at a higher level of theory, namely, DLPNO-CCSD(T)-F12/cc-pVTZ-F12//M06-2X/6-311++G(d,p). Rate coefficients for specific reaction channels have been computed by means of conventional TST. The interplay between thermodynamics and kinetics leads to the identification of xylopyranose ring-opening pathway as the main initial reactive channel. Accordingly, our electronic structure calculations concentrate exclusively on the reactive pathways associated with the ring-opening reaction. Our calculations are in good agreement with previously reported works. From the open-chain xylose, a series of thermodynamically stable, key products can be formed: furfural, anhydro-d-xylopyranose, glycolaldehyde, and dihydrofuran-3(2*H*)-one. In particular, furfural revests a key role among renewable chemicals since it can be transformed to a variety of C_4_ and C_5_ chemicals, e.g., ethyl levulinate (EL), which is a an important biofuel additive.^56−58^ For the first time, enthalpies of formation, heat capacities at constant pressure, and reaction barriers are reported for the key species involved in the xylose pyrolytic system. By applying TST and solving the kinetic model through Cantera simulations, different pyrolytic reactive regimes of xylopyranose have been replicated, namely, fixed temperature and increasing temperature ones (TGA-like). While the kinetic model remains incomplete and will undergo future expansion and validation, from a methodological perspective, the proposed approach can pave the way to an iterative exploration of potential energy surfaces for reactive systems of this nature. Indeed, the idea is to include new reactions into the model only if their contribution to the total reaction flux is above a certain threshold; otherwise, these reactions are excluded.

In perspective, this iterative interplay between PES exploration and kinetic model validation could be automatized through the use of codes that automatically search for reaction mechanisms.^59−64^ Future enhancements to the model will involve the incorporation of additional reaction pathways and a comparison of the kinetic model’s results with experimental data.

## Supporting Information Available

Intrinsic reaction coordinate paths; thermodynamic properties NASA polynomials fitting;  diagnostic; Cantera kinetic model input file; MESS input files; and geometries of stationary points optimized at M06-2X/6-311++G(d,p) (PDF)
