# Supplementary material for: Ab Initio and Kinetic Modeling of β-d-Xylopyranose under Fast Pyrolysis Conditions
Source: J Phys Chem A. 2024 Feb 1;128(6):1009–24. doi: 10.1021/acs.jpca.3c07063 (PMC10875674; doi:10.1021/acs.jpca.3c07063)
Supplement: Supplementary file 1 — jp3c07063_si_001.pdf [file jp3c07063_si_001.pdf]

Supporting Information: Ab Initio and Kinetic Modelling of β-D-xylopyranose Under Fast Pyrolysis Conditions

Jacopo Lupi,*,†,‡ Leandro Ayarde-Henríquez,†,‡ Mark Kelly,†,‡ and Stephen Dooley*,†,‡

†*School of Physics, Trinity College Dublin, Dublin 2, Ireland*

‡*AMBER, Advance Materials and BioEngineering Research Centre, Dublin 2, Ireland*

E-mail: jacopo.lupi@tcd.ie; stephen.dooley@tcd.ie

Contents

1 Intrinsic Reaction Coordinate paths **S4**

2 Thermodynamic properties through NASA polynomials **S4**

3 $\mathcal{T}_1$ diagnostic **S4**

4 Cantera kinetic model **S8**

5 MESS input files **S12**

5.1 Xylopyranose $\longrightarrow$ Xylose S12

5.2 Xylopyranose $\longrightarrow$ TH−C−OH2−c + H_2O S18

5.3 Xylopyranose $\longrightarrow$ TH−C−OH2−t + H_2O S24

5.4 Xylopyranose $\longrightarrow$ $AXP_1 + H_2O$ S30

5.5 Xylopyranose $\longrightarrow$ $AXP_{2-1} + H_2O$ S37

5.6 Xylopyranose $\longrightarrow$ $AXP_{2-3} + H_2O$ S43

5.7 Xylopyranose $\longrightarrow$ $AXP_{3-2} + H_2O$ S49

5.8 Xylopyranose $\longrightarrow$ $AXP_{3-4} + H_2O$ S55

5.9 Xylopyranose $\longrightarrow$ $AXP_{4-3} + H_2O$ S62

5.10 Xylopyranose $\longrightarrow$ $AXP_{4-5} + H_2O$ S68

6 Geometries of stationary points **S74**

6.1 Xylopyranose decompositions S74

6.1.1 Ring opening S74

6.1.2 Dehydrations S77

6.1.3 Ring contractions S89

6.2 Xylose decompositions S92

6.2.1 Dehydration S92

6.2.2 Cyclization S107

6.2.3 C−C scission S110
6.2.4 Isomerisation S113

1 Intrinsic Reaction Coordinate paths

IRC paths connecting transition states to minima have been uploaded on Zenodo and can be found at this link `https://zenodo.org/records/10230818`.

2 Thermodynamic properties through NASA polynomials

For convenience, the thermodynamic properties for each chemical species are expressed in the NASA polynomial format as follows:

$$\frac{\hat{C_p}^{\circ}(T)}{R} = a_0 + a_1 T + a_2 T^2 + a_3 T^3 + a_4 T^4, \tag{1}$$

$$\frac{\hat{H}_f^{\circ}(T)}{RT} = a_0 + \frac{a_1}{2} T + \frac{a_2}{3} T^2 + \frac{a_3}{4} T^3 + \frac{a_4}{5} T^4 + \frac{a_5}{T}, \tag{2}$$

$$\frac{\hat{S}^{\circ}(T)}{R} = a_0 \ln T + a_1 T + \frac{a_2}{2} T^2 + \frac{a_3}{3} T^3 + \frac{a_4}{4} T^4 + a_6. \tag{3}$$

This format allows for the expression of the temperature dependence of the thermodynamic properties of each chemical species. These a_x coefficients must be provided in the kinetic model for each species in the system. Hence, in this work, the molar heat capacity at constant pressure, molar enthalpy of formation, and molar entropy of each chemical species are computed at a range of temperatures and fitted to the NASA format using the THERM code.

3 $\mathcal{T}_1$ diagnostic

**Species**	**$\mathcal{T}_1$ Diagnostic**
C2	0.013

C1	0.012
FD	0.015
HAA	0.014
TS-C2C1	0.017
TS-C1HAA	0.018
TC-C	0.015
B1	0.011
ADX	0.011
H_2O	0.010
TS-B1ADX	0.016
TS-B	0.013
A2-b1	0.014
A1	0.013
A2	0.014
A2-c1	0.013
A2-a1	0.013
A2-c2	0.014
A2-c3	0.014
A2-a2	0.014
FF	0.015
MGO	0.015
TS-MGO	0.018
TS-A1A2	0.015
TS-A2c1	0.014
TS-A2a1	0.014
TS-A2b1	0.016
TS-A2c12	0.016

TS-A2c13	0.018
TS-A2a12	0.019
TS-A2c2FF	0.018
TS-A2c3FF	0.020
TS-A	0.014
AD	0.014
CO	0.018
D1-a1	0.011
D1-a2	0.012
D1-a3	0.013
D1-c1	0.012
D1-b1	0.014
DHA	0.013
ETH	0.012
TS-D1a12	0.015
TS-D1a2c3	0.016
TS-D1a23	0.016
TS-D1a3FF	0.018
TS-D1c1DHA	0.016
TS-D1b1HAA	0.018
TS-ETHAD	0.019
TS-D	0.015
D1	0.012
D1-d5	0.014
TS-DFO	0.017
D1-d1	0.013
D1-d2	0.013

D1-d3	0.013
D1-d6	0.014
D1-d4	0.014
D1-d7	0.014
TS-D1d1	0.014
TS-D1d2	0.015
TS-D1d3	0.014
TS-D1d6	0.016
TS-D1d4	0.019
TS-D1d7	0.016
TS-D1d5	0.017
TS-DFO	0.017
DFO	0.013
AXP_{4-5}	0.012
AXP_{4-3}	0.012
AXP_{3-4}	0.012
AXP_{3-2}	0.012
AXP_{2-3}	0.012
AXP_{2-1}	0.012
AXP_1	0.012
TH−C−OH2−c	0.012
TH−C−OH2−t	0.013
TS−AXP_{4-5}	0.016
TS−AXP_{4-3}	0.016
TS−AXP_{3-4}	0.016
TS−AXP_{3-2}	0.014
TS−AXP_{2-3}	0.014

TS−AXP$_{2-1}$	0.015
TS−AXP$_1$	0.016
TS−TH−C−OH2−c	0.016
TS−TH−C−OH2−t	0.016
TS-Xylose	0.012
Xylopyranose	0.011
Xylose	0.012
C(^{3}P)	0.02
O(^{3}P)	0.02

4 Cantera kinetic model

```
# Kinetic model of the pyrolysis of xylopyranose
units(time='s', quantity='mol', act_energy='cal/mol')


ideal_gas(name='biomass',
          elements="C H O N Ar He",
          species="""XYLPYRANOSE XYLOSE THCOH2-c H2O THCOH2-t
                     AXP1 AXP2-1 AXP2-3 AXP3-2 AXP3-4 AXP4-3 AXP4-5
 A1 B1 C1 C2 D1
                     """,
          reactions='all',
          initial_state=state(temperature=300.0, pressure=OneAtm))



#-----------------------------------------------------------------------------------------------------------------------
# Species data
#-----------------------------------------------------------------------------------------------------------------------
#---------XYLOPYRANOSE SPECIES-------------------------------------------------------------------------------------------------
species(name='XYLPYRANOSE',
        atoms='C:5 H:10 O:5',
        thermo=(NASA([300.00, 1406.00],
                     [ 2.49780875E+01, 2.69185990E-02, -9.05065171E-06, 1.38943725E-09, -7.99958269E-14, -1.17895625E+05, -1.08736372E+02 ]),
                NASA([1406.00, 5000.00],
                     [-2.65400291E+00, 9.69870855E-02, -7.73842184E-05, 3.17132644E-08, -5.21911434E-12, -1.08970489E+05, 3.75072158E+01 ])))

species(name='XYLOSE',
        atoms='C:5 H:10 O:5',
        thermo=(NASA([300.00, 1406.00],
                     [2.49986873E+01, 2.69495939E-02, -9.06958437E-06, 1.39305827E-09, -8.02276646E-14, -1.15594775E+05, -1.07504846E+02 ]),
                NASA([1406.00, 5000.00],
                     [-2.23787169E+00, 9.72741171E-02, -7.93710822E-05, 3.35153576E-08, -5.69606739E-12, -1.06888770E+05, 3.62563810E+01 ])))
```

```
species(name='AXP1',
        atoms='C:5 H:8 O:4',
        thermo=(NASA([300.00, 1406.00],
                     [2.18031893E+01, 2.21890022E-02, -7.46463135E-06, 1.14638839E-09, -6.60199283E-14, -7.98351713E+04, -9.13650716E+01 ]),
                NASA([1406.00, 5000.00],
                     [-2.49586345E+00, 8.55649305E-02, -7.13237938E-05, 3.04787418E-08, -5.20842323E-12, -7.21544244E+04, 3.66279212E+01 ])))

species(name='AXP2-1',
        atoms='C:5 H:8 O:4',
        thermo=(NASA([300.00, 1406.00],
                     [2.18031893E+01, 2.21890022E-02, -7.46463135E-06, 1.14638839E-09, -6.60199283E-14, -7.98351713E+04, -9.13650716E+01 ]),
                NASA([1406.00, 5000.00],
                     [-2.49586345E+00, 8.55649305E-02, -7.13237938E-05, 3.04787418E-08, -5.20842323E-12, -7.21544244E+04, 3.66279212E+01 ])))

species(name='AXP2-3',
        atoms='C:5 H:8 O:4',
        thermo=(NASA([300.00, 1406.00],
                     [2.16444065E+01, 2.23573199E-02, -7.53005766E-06, 1.15736608E-09, -6.66900432E-14,  -8.34832888E+04,  -9.08417686E+01 ]),
                NASA([1406.00, 5000.00],
                     [-3.24423062E+00, 8.75755299E-02, -7.36280218E-05, 3.17123452E-08, -5.45800091E-12,  -7.56412771E+04,   4.01578934E+01 ])))

species(name='AXP3-2',
        atoms='C:5 H:8 O:4',
        thermo=(NASA([300.00, 1406.00],
                     [2.16854517E+01, 2.23146293E-02, -7.51367629E-06, 1.15464242E-09, -6.65248819E-14,  -8.31608253E+04,  -9.10625813E+01 ]),
                NASA([1406.00, 5000.00],
                     [-3.21529048E+00, 8.76696612E-02, -7.38678163E-05, 3.18823852E-08, -5.49725094E-12,  -7.53251621E+04,   3.99641068E+01 ])))

species(name='AXP3-4',
        atoms='C:5 H:8 O:4',
        thermo=(NASA([300.00, 1406.00],
                     [2.17439640E+01, 2.22495462E-02, -7.48790311E-06, 1.15027930E-09, -6.62572466E-14, -8.37159932E+04, -9.14060963E+01 ]),
                NASA([1406.00, 5000.00],
                     [-3.04797056E+00, 8.73013918E-02, -7.34929863E-05, 3.16887437E-08, -5.45774968E-12, -7.59152261E+04,  3.90501979E+01 ])))

species(name='AXP4-3',
        atoms='C:5 H:8 O:4',
        thermo=(NASA([300.00, 1405.00],
                     [2.16699636E+01, 2.23858974E-02, -7.55080493E-06, 1.16172105E-09, -6.69882638E-14,  -8.33168771E+04,  -9.09822391E+01 ]),
                NASA([1405.00, 5000.00],
                     [-2.55688787E+00, 8.48849273E-02, -6.98233180E-05, 2.94671545E-08, -4.98436440E-12,  -7.55849082E+04,   3.68836223E+01 ])))

species(name='AXP4-5',
        atoms='C:5 H:8 O:4',
        thermo=(NASA([300.00, 1408.00],
                     [2.17488415E+01, 2.22158424E-02, -7.46983525E-06, 1.14679591E-09, -6.60278306E-14,  -8.36944064E+04,  -9.21519737E+01 ]),
                NASA([1408.00, 5000.00],
                     [-3.94902499E+00, 9.05978175E-02, -7.78414305E-05, 3.41306697E-08, -5.95450857E-12,  -7.57089384E+04,   4.27246754E+01 ])))

species(name='THCOH2-c',
        atoms='C:5 H:8 O:4',
        thermo=(NASA([300.00, 1403.00],
                     [2.14595236E+01,  2.28324837E-02, -7.76248131E-06,  1.20064221E-09, -6.94887961E-14,  -8.16346773E+04,  -8.92399779E+01 ]),
                NASA([1403.00, 5000.00],
                     [-2.78418309E+00,  8.34511371E-02, -6.60365291E-05,  2.67147031E-08, -4.34315075E-12,  -7.37051845E+04,   3.93969674E+01 ])))
```

```
species(name='THCOH2-t',
        atoms='C:5 H:8 O:4',
        thermo=(NASA([300.00, 1404.00],
                     [2.15833423E+01,  2.26832909E-02, -7.70147009E-06, 1.19013876E-09, -6.88379839E-14,  -8.13232283E+04, -8.97092399E+01  ]),
                NASA([1404.00, 5000.00],
                     [-2.63012046E+00,  8.35816307E-02, -6.66370208E-05, 2.71752828E-08, -4.45136295E-12,  -7.34404439E+04,  3.86389919E+01   ])))

species(name='H2O',
        atoms='C:0 H:2 O:1',
        thermo=(NASA([300.00, 1612.00],
                     [2.69405642E+00, 2.91342241E-03, -8.44269107E-07,  1.15325994E-10, -6.05732068E-15,  -3.00545010E+04, 6.81403842E+00  ]),
                NASA([1612.00, 5000.00],
                     [3.88299529E+00, 6.19091267E-05,  1.72704490E-06, -9.26315808E-10,  1.55265050E-13,  -3.04492571E+04, 4.71351995E-01   ])))

species(name='A1',
        atoms='C:5 H:8 O:4',
        thermo=(NASA([300.00, 1403.00],
                     [2.20765007E+01, 2.19812856E-02, -7.39644389E-06,  1.13595359E-09, -6.54160504E-14,  -7.75356836E+04, -8.83850414E+01  ]),
                NASA([1403.00, 5000.00],
                     [8.29963867E-01, 7.60319897E-02, -6.05917153E-05,  2.50815626E-08, -4.19657181E-12,  -7.06579710E+04,  2.40553993E+01   ])))


species(name='B1',
        atoms='C:5 H:10 O:5',
        thermo=(NASA([300.000, 1408.000],
                     [2.50927027E+01, 2.67628658E-02, -8.98388513E-06, 1.37764625E-09, -7.92535393E-14, -1.16531690E+05, -1.08786494E+02]),
                NASA([1408.000, 5000.000],
                     [-3.59647221E+00, 1.01283289E-01, -8.36404930E-05, 3.54322094E-08, -6.00480758E-12, -1.07443120E+05, 4.24216425E+01])))

species(name='C1',
        atoms='C:2 H:4 O:2',
        thermo=(NASA([300.000, 1411.000],
                     [9.66817211E+00, 9.75467333E-03, -3.21136261E-06, 4.85719100E-10, -2.76660724E-14, -3.71530983E+04, -2.56112323E+01]),
                NASA([1411.000, 5000.000],
                     [5.27893963E-01, 3.47936408E-02, -2.98233333E-05, 1.33600089E-08, -2.39248650E-12, -3.43745531E+04, 2.21236995E+01])))

species(name='C2',
        atoms='C:3 H:6 O:3',
        thermo=(NASA([300.000, 1400.000],
                     [1.45765120E+01, 1.58996837E-02, -5.35877165E-06, 8.23867005E-10, -4.74771981E-14, -6.30477022E+04, -4.97688546E+01]),
                NASA([1400.000, 5000.000],
                     [1.64191594E+00, 4.62318980E-02, -3.23479381E-05, 1.16531685E-08, -1.69977078E-12, -5.86019217E+04, 1.95982465E+01])))

species(name='D1',
        atoms='C:5 H:10 O:5',
        thermo=(NASA([300.000, 1405.000],
                     [2.52982330E+01, 2.66040581E-02, -8.93068163E-06, 1.36935573E-09, -7.87666925E-14, -1.14902239E+05, -1.06070638E+02]),
                NASA([1405.000, 5000.000],
                     [1.69037888E-01, 8.99751114E-02, -7.05400088E-05, 2.86955402E-08, -4.71800379E-12, -1.06726874E+05, 2.70898135E+01])))


#------------------------------------------------------------------------------------------------------------------------
# Reaction data
#------------------------------------------------------------------------------------------------------------------------
#----------XYLOPYRANOSE REACTIONS-------------------------------------------------------------------------------------------
```

```
# Reaction || 1
# Ring opening
reaction('XYLPYRANOSE <=> XYLOSE', [2.53e12, 0.0, 43451.2908])

# Reaction || 2
# Ring contraction to (THCOH2-c) dihydroxytetrahydrofuran-2-carbaldehyde isomer cis.
#reaction('XYLPYRANOSE <=> THCOH2-c + H2O', [7.08e13, 0.0, 72920.7306])

# Reaction || 3
# Ring contraction to (THCOH2-t) dihydroxytetrahydrofuran-2-carbaldehyde isomer cis.
#reaction('XYLPYRANOSE <=> THCOH2-t + H2O', [6.42e13, 0.0, 67112.8848])

# Reaction || 4
# Dehydration elimination at 1 position to 1-anhydro xylopyranose, AXP1.
#SD - change to D1
#reaction('XYLPYRANOSE <=> AXP1 + H2O', [4.6e14, 0.0, 69240.0382 ])

# Reaction || 5
# Dehydration elimination at 2 position to 2-anhydro xylopyranose-1, AXP2-1.
# D2-1, meaning loss of OH at 2 and loss of H at 1.
#reaction('XYLPYRANOSE <=> AXP2-1 + H2O', [2.76e14, 0.0, 83628.1994])

# Reaction || 6
# Dehydration elimination at 2 position to 2-anhydro xylopyranose-3, AXP2-3.
#reaction('XYLPYRANOSE <=> AXP2-3 + H2O', [1.23e14, 0.0, 74044.0588])

# Reaction || 7
# Dehydration elimination at 3 position to 3-anhydro xylopyranose-2, AXP3-2.
#reaction('XYLPYRANOSE <=> AXP3-2 + H2O', [4.58e13, 0.0, 70602.3724])

# Reaction || 8
# Dehydration elimination at 3 position to 3-anhydro xylopyranose-4, AXP3-4.
#reaction('XYLPYRANOSE <=> AXP3-4 + H2O', [2.32e13, 0.0, 70793.5772])

# Reaction || 9
# Dehydration elimination at 4 position to 4-anhydro xylopyranose-3, AXP4-3.
#reaction('XYLPYRANOSE <=> AXP4-3 + H2O', [1.26e13, 0.0, 70865.2790])

# Reaction || 10
# Dehydration elimination at 4 position to 4-anhydro xylopyranose-5, AXP4-5.
#reaction('XYLPYRANOSE <=> AXP4-5 + H2O', [8.83e12, 0.0, 67710.3998])

# Reaction || 11
# Dehydration elimination from XYLOSE open chain, at 3 position
reaction('XYLOSE <=> A1 + H2O', [1.17e15, 0.0, 65344.2404])

# Reaction || 12
# Cyclization reaction from XYLOSE open chain to five member intermediate B1 (xylofuranose)
reaction('XYLOSE <=> B1', [4.17e12, 0.0, 37380.5384])

# Reaction || 13
# Carbon-carbon bond fission reaction from XYLOSE open chain to C1 (enol isomer of glycolaldehyde) and C2 (glyceraldehyde)
reaction('XYLOSE <=> C1 + C2', [1.76e14, 0.0, 39794.4990])

# Reaction || 14
```

```
# Isomerization reaction from XYLOSE open chain to D1 (D-xylulose)
reaction('XYLOSE <=> D1', [4.38e13, 0.0, 43666.3962])
```

5 MESS input files

5.1 Xylopyranose ⟶ Xylose

```
!***************************************************
!              GLOBAL SECTION
!***************************************************
!!!!!!!!!!!!!!!!!!!!!!!!!!!!!!!!!!!!!!!!!!!!!!!!!!!!
!
!
TemperatureList[K]                      300 320 340 360 380 400 420 440 460 480 500 520 540 560 580 600 620 640 660
680 700 720 740 760 780 800 820 840 860 880 900 920 940 960 980 1000
PressureList[atm]                       1
!
!
EnergyStepOverTemperature               .2         ! [Discretization energy step (global relax matrix)] / T
ExcessEnergyOverTemperature             30         ! [Highest barrier in the model (global relax matrix)] / T
ModelEnergyLimit[kcal/mol]              400        ! Highest reference energy used in the calculation ( or ReferenceEnergy[kcal/mol])
!
CalculationMethod                       direct     ! direct or low-eigenvalue
!
WellCutoff                              20         ! well truncation parameter : Max { dissociation limit (min barrier rel. to bottom of the well) / T }
ChemicalEigenvalueMax                   0.2        ! Max chemical eigenvalue / Lowest Collision relaxation eigenvalue
!
ReductionMethod                         diagonalization ! [low eigenvalue method only] diagonalization or projection (default)
!
!!!!!!!!!test!!!!!!!!!!!!!!!!!!!
!WellCutoff                            10
!ChemicalEigenvalueMin                 1.e-6          #only for direct diagonalization method
!!!!!!!!test!!!!!!!!!!!!!!!!!!!!!!!!!
AtomDistanceMin[bohr]                  1.3
!!
RateOutput                              ro.out                        ! output file name for rate coefficients
!
!
!!!!!!!!!!!!!!!!!!!!!!!!!!!!!!!!!!!!!!!!!!!!!!!!!!!!
!***************************************************
!              MODEL SECTION
!***************************************************
!!!!!!!!!!!!!!!!!!!!!!!!!!!!!!!!!!!!!!!!!!!!!!!!!!!!
!
!
Model
!
  EnergyRelaxation                                                          ! Default collisional energy relaxation kernel
    Exponential                                                             ! Currently the only possible energy relaxation model
       Factor[1/cm]                     260                                 ! (Delta_E_down)^(0) @ standard T (300 K)
       Power                            0.875                               ! Power n in the expression (Delta_E_down) = (Delta_E_down)^(0) (T/T0)^(n)
       ExponentCutoff                   10                                  ! if (Delta_E) / (Delta_E_down) > value  transition probability is zero
```

```
    End
!
  CollisionFrequency                                               ! Collision frequency model
    LennardJones                                                   ! Currently the only possible collisional frequency model  based on LJ potential
       Epsilons[K]                      90.58  617.0               ! Epsilon_1 and Epsilon_2 (630.4 x kB x Na = 1.25)(cm-1 to K = x 1.4)
       Sigmas[angstrom]                 3.54    5.62               ! Sigma_1 and Sigma_2
       Masses[amu]                      39.948 69.0                ! Masses of the buffer gas molecule and of the complex (check order)
   End
!
!**************************************************
!
!****************************************************
!!!!!!!!!!!!!!!!!!!!!!!!!!!!!!!!!!!!!!!!!!!!!!!!!!!!
!****************************************************
!  XILOSIO 1
!****************************************************
 Well 1
 Species
 RRHO     ! well
 Geometry[angstrom]              20
 C                -1.38809900   -0.54955600   -0.28755900
 C                -0.56453100   -1.72483200    0.22988200
 C                 1.43761800   -0.47439000    0.24809800
 C                 0.72705700    0.79121500   -0.21246600
 C                -0.73922400    0.73445100    0.18821700
 H                -0.59297400   -1.73232800    1.32855500
 H                -0.95818500   -2.67540600   -0.13228500
 H                -1.40210300   -0.56163500   -1.38286400
 H                 1.44588400   -0.51556600    1.34928600
 H                 0.80376600    0.83417500   -1.30627000
 H                -0.80066100    0.75804700    1.28587100
 O                 0.76641900   -1.62705800   -0.25011700
 O                 2.72158200   -0.47081700   -0.27369400
 H                 3.18204700   -1.24694200    0.06192100
 O                 1.36273200    1.90326300    0.38748700
 H                 0.81522200    2.67245500    0.19230800
 O                -1.36055300    1.89033900   -0.35564600
 H                -2.29057100    1.85752700   -0.10551800
 O                -2.71496800   -0.55293900    0.23716300
 H                -3.24104700   -1.19396100   -0.24958400
  Core  RigidRotor
  SymmetryFactor    1.0000000000000000
  End
     Frequencies[1/cm]           54
97.25
109.60
231.23
244.30
250.96
257.36
271.96
289.72
326.49
356.54
372.54
399.94
```

```
415.21
449.07
515.75
523.37
577.83
635.32
886.53
962.82
992.35
1009.61
1051.19
1063.43
1075.47
1094.19
1104.55
1114.74
1149.92
1163.52
1204.41
1222.85
1232.51
1270.06
1307.70
1309.25
1323.62
1334.25
1354.37
1366.52
1401.57
1409.76
1454.09
1467.04
2885.85
2914.21
2920.19
2946.91
2963.21
3021.01
3701.33
3709.12
3719.95
3733.18
  ZeroEnergy[kJ/mol]     0.00
  ElectronicLevels[1/cm]                      1
   0.0000000000000000        1.
 End
 End

!***************************************************
!  OPENED 2
!***************************************************
 Well 2
 Species
 RRHO     ! well
 Geometry[angstrom]               20
 C                 -0.84681400   -0.92445500   -0.03987600
```

```
 C                -2.31654600   -0.54696100    0.12435900
 C                 1.82814700    0.63840300   -0.61716700
 C                 1.53257000   -0.31626500    0.52952100
 C                 0.06046400   -0.23221600    0.98507300
 H                -2.93488700   -1.22520500   -0.46444300
 H                -2.60969000   -0.62998600    1.17646800
 H                -0.74210900   -2.00422200    0.09907800
 H                 2.33389200    0.18539300   -1.48773100
 H                 2.16689600   -0.00767600    1.36563900
 H                -0.03268700   -0.77344500    1.93160900
 O                -2.55825200    0.75710600   -0.38486500
 O                 1.57623600    1.82692700   -0.58069200
 H                -2.06322800    1.35299800    0.19494900
 O                 1.91381500   -1.63547800    0.20026500
 H                 1.46747600   -1.87189600   -0.62218800
 O                -0.32055600    1.11733200    1.24436200
 H                 0.25072100    1.69565800    0.71009100
 O                -0.41580300   -0.63505400   -1.37052900
 H                -0.94684500    0.12067900   -1.66324500
  Core  RigidRotor
  SymmetryFactor    1.0000000000000000
  End
     Frequencies[1/cm]            54
66.19
86.21
166.29
174.47
199.53
239.99
275.72
290.42
312.09
392.36
413.93
456.30
479.42
495.04
504.75
581.04
637.09
675.53
771.24
790.85
869.26
932.68
958.95
989.78
1071.47
1088.78
1105.88
1120.11
1130.29
1194.23
1205.03
1230.98
1262.72
```

```
1277.94
1333.33
1349.58
1357.38
1365.39
1382.07
1387.02
1400.99
1416.13
1460.12
1785.49
2909.11
2971.05
2982.26
2994.27
2999.81
3040.20
3620.50
3679.02
3719.00
3722.82
  ZeroEnergy[kJ/mol]     18.2
  ElectronicLevels[1/cm]                      1
   0.0000000000000000        1.
 End
 End
!*********
!************
! TS-RO
!************
 Barrier TSRO 1 2
 RRHO           !
 Geometry[angstrom]           20
 O                -2.55104400    0.02081100   -0.11237300
 O                 0.42262000    2.04978100   -0.09199000
 O                -0.50633500   -1.00624400    1.62008400
 O                 2.36281200    0.17529300   -0.28462900
 O                 0.76857800   -0.96914200   -1.12418200
 C                -0.39269400    1.03930800    0.44548100
 C                 0.42638700   -0.02835300    1.17464200
 C                 1.46894700   -0.64698300    0.24869300
 C                -0.65807000   -0.83318100   -1.31975600
 C                -1.29264900    0.39569400   -0.63305200
 H                -2.38785600   -0.54789600    0.64969400
 H                 1.32375000    1.70346900   -0.20731300
 H                -0.09474200   -1.59325900    2.25922800
 H                 1.55763600   -0.19746700   -1.35350800
 H                -1.07699600    1.49803300    1.16526900
 H                 0.96779900    0.42853300    2.01015200
 H                 1.82440500   -1.62662900    0.59801000
 H                -1.49074500    1.17743900   -1.36656400
 H                -0.80617500   -0.82648200   -2.39833500
 H                -1.10165400   -1.73863700   -0.90795700
  Core  RigidRotor
          SymmetryFactor   1
    End
```

```
Tunneling    Eckart
    ImaginaryFrequency[1/cm]    1298.73
    WellDepth[kJ/mol]   183.8
    WellDepth[kJ/mol]   165.7
    End
     Frequencies[1/cm]            53
74.24
166.81
180.51
232.00
248.90
270.60
329.52
339.41
409.36
451.51
480.34
495.30
528.85
561.39
600.58
624.72
731.34
782.25
832.79
895.00
993.27
1009.63
1046.62
1074.43
1083.66
1098.41
1106.60
1131.00
1180.56
1206.51
1229.29
1253.35
1267.44
1284.90
1314.11
1320.97
1338.00
1359.74
1374.24
1409.17
1431.34
1444.56
1453.38
2173.48
2942.19
2979.42
2991.23
3024.19
3031.16
3080.63
```

```
3543.20
3730.33
3789.15
     ZeroEnergy[kJ/mol]      183.8
     ElectronicLevels[1/cm]                     1
     0.0000000000000000        1.0000000000000000
     End
  End
```

5.2 Xylopyranose $\longrightarrow$ TH$-$C$-$OH2$-$c + H_2O

```
!***************************************************
!               GLOBAL SECTION
!***************************************************
!!!!!!!!!!!!!!!!!!!!!!!!!!!!!!!!!!!!!!!!!!!!!!!!!!!!
!
!
TemperatureList[K]                      300 320 340 360 380 400 420 440 460 480 500 520 540 560 580 600 620 640
 660 680 700 720 740 760 780 800 820 840 860 880 900 920 940 960 980 1000
PressureList[atm]                       1
!
!
EnergyStepOverTemperature               .2         ! [Discretization energy step (global relax matrix)] / T
ExcessEnergyOverTemperature             30         ! [Highest barrier in the model (global relax matrix)] / T
ModelEnergyLimit[kcal/mol]              400        ! Highest reference energy used in the calculation ( or ReferenceEnergy[kcal/mol])
!
CalculationMethod                       direct     ! direct or low-eigenvalue
!
WellCutoff                              20         ! well truncation parameter : Max { dissociation limit (min barrier rel. to bottom of the well) / T }
ChemicalEigenvalueMax                   0.2        ! Max chemical eigenvalue / Lowest Collision relaxation eigenvalue
!
ReductionMethod                         diagonalization ! [low eigenvalue method only] diagonalization or projection (default)
!
!!!!!!!!!test!!!!!!!!!!!!!!!!!!
!WellCutoff                            10
!ChemicalEigenvalueMin                 1.e-6          #only for direct diagonalization method
!!!!!!!!test!!!!!!!!!!!!!!!!!!!!!!!!!
AtomDistanceMin[bohr]                  1.3
!!
RateOutput                             rc1.out                        ! output file name for rate coefficients
!
!
!!!!!!!!!!!!!!!!!!!!!!!!!!!!!!!!!!!!!!!!!!!!!!!!!!!!
!***************************************************
!               MODEL SECTION
!***************************************************
!!!!!!!!!!!!!!!!!!!!!!!!!!!!!!!!!!!!!!!!!!!!!!!!!!!!
!
!
Model
```

```
!
  EnergyRelaxation                                                   ! Default collisional energy relaxation kernel
    Exponential                                                      ! Currently the only possible energy relaxation model
       Factor[1/cm]                    260                           ! (Delta_E_down)^(0) @ standard T (300 K)
       Power                           0.875                         ! Power n in the expression (Delta_E_down) = (Delta_E_down)^(0) (T/T0)^(n)
       ExponentCutoff                  10                            ! if (Delta_E) / (Delta_E_down) > value  transition probability is zero
    End
!
  CollisionFrequency                                                 ! Collision frequency model
    LennardJones                                                     ! Currently the only possible collisional frequency model  based on LJ potential
       Epsilons[K]                      90.58  617.0                 ! Epsilon_1 and Epsilon_2 (630.4 x kB x Na = 1.25)(cm-1 to K = x 1.4) Ar and c7h7o2
       Sigmas[angstrom]                 3.54    5.62                 ! Sigma_1 and Sigma_2
       Masses[amu]                      39.948 69.0                  ! Masses of the buffer gas molecule and of the complex (check order)
   End
!
!**************************************************
!
!****************************************************
!!!!!!!!!!!!!!!!!!!!!!!!!!!!!!!!!!!!!!!!!!!!!!!!!!!!
!****************************************************
!  XILOSIO 1
!****************************************************
 Well 1
 Species
 RRHO     ! well
 Geometry[angstrom]               20
 C                 -1.38809900   -0.54955600   -0.28755900
 C                 -0.56453100   -1.72483200    0.22988200
 C                  1.43761800   -0.47439000    0.24809800
 C                  0.72705700    0.79121500   -0.21246600
 C                 -0.73922400    0.73445100    0.18821700
 H                 -0.59297400   -1.73232800    1.32855500
 H                 -0.95818500   -2.67540600   -0.13228500
 H                 -1.40210300   -0.56163500   -1.38286400
 H                  1.44588400   -0.51556600    1.34928600
 H                  0.80376600    0.83417500   -1.30627000
 H                 -0.80066100    0.75804700    1.28587100
 O                  0.76641900   -1.62705800   -0.25011700
 O                  2.72158200   -0.47081700   -0.27369400
 H                  3.18204700   -1.24694200    0.06192100
 O                  1.36273200    1.90326300    0.38748700
 H                  0.81522200    2.67245500    0.19230800
 O                 -1.36055300    1.89033900   -0.35564600
 H                 -2.29057100    1.85752700   -0.10551800
 O                 -2.71496800   -0.55293900    0.23716300
 H                 -3.24104700   -1.19396100   -0.24958400
  Core  RigidRotor
  SymmetryFactor    1.0000000000000000
  End
     Frequencies[1/cm]            54
97.25
109.60
231.23
244.30
250.96
257.36
```

```
271.96
289.72
326.49
356.54
372.54
399.94
415.21
449.07
515.75
523.37
577.83
635.32
886.53
962.82
992.35
1009.61
1051.19
1063.43
1075.47
1094.19
1104.55
1114.74
1149.92
1163.52
1204.41
1222.85
1232.51
1270.06
1307.70
1309.25
1323.62
1334.25
1354.37
1366.52
1401.57
1409.76
1454.09
1467.04
2885.85
2914.21
2920.19
2946.91
2963.21
3021.01
3701.33
3709.12
3719.95
3733.18
  ZeroEnergy[kJ/mol]     0.00
  ElectronicLevels[1/cm]                     1
   0.0000000000000000        1.
 End
 End

!***************************************************
!  PRODUCT RC1+H2O
```

```
!**************************************************
 Bimolecular PROD
 Fragment C5H8O4
 RRHO
 Geometry[angstrom]              17
 C                 0.07653500    0.84888000   -0.20689300
 C                 1.51995000    0.47578100    0.13927800
 C                -0.50161500   -0.54454200   -0.49716800
 C                 1.32245600   -0.81377000    0.92016500
 O                 2.25302500    0.16422800   -1.03059600
 H                 1.99851000    1.25776200    0.73441200
 O                -0.47958500    1.44990000    0.93777100
 H                 0.01550300    1.50494100   -1.08124600
 C                -1.98446400   -0.66025600   -0.23084400
 O                 0.19033900   -1.46089000    0.33277900
 H                -0.32617100   -0.79731700   -1.55320100
 H                 2.18906100   -1.46864600    0.82991700
 H                 1.11282200   -0.59768000    1.97040000
 H                 2.40916800    0.97129400   -1.52789600
 H                -1.41202500    1.62125600    0.76584200
 O                -2.74550300    0.26294800   -0.36250800
 H                -2.33024400   -1.65765300    0.09496800
  Core  RigidRotor
  SymmetryFactor    1
  End
     Frequencies[1/cm]           45
76.06
114.73
138.55
198.29
233.43
295.67
311.23
379.75
417.89
470.03
490.60
652.52
706.42
758.44
838.77
872.73
935.02
952.87
962.64
1019.03
1071.73
1096.34
1132.93
1139.91
1165.59
1192.99
1228.52
1277.14
1285.95
1299.73
```

```
1327.03
1343.18
1359.95
1373.54
1400.85
1463.92
1797.54
2914.05
2930.47
2979.97
2991.90
3006.49
3055.47
3746.65
3781.63
  ZeroEnergy[kJ/mol]            0.
  ElectronicLevels[1/cm]                     1
   0.       1
    !************************************
 End
!*********
 Fragment H2O
 RRHO
 Geometry[angstrom]              3
 O                 0.00000000    0.00000000    0.11661200
 H                 0.00000000    0.76156400   -0.46644700
 H                 0.00000000   -0.76156400   -0.46644700
  Core  RigidRotor
  SymmetryFactor    2
  End
     Frequencies[1/cm]           3
1550.43
3775.37
3877.81
  ZeroEnergy[kJ/mol]            0.
  ElectronicLevels[1/cm]                     1
   0.       1
   End
    !************************************
GroundEnergy[kJ/mol]   39.2
End
!************
! TS-RC1
!************
 Barrier TSR0 1 PROD
 RRHO           !
 Geometry[angstrom]           20
 C                 1.60322100   -0.32679000    0.30022400
 C                 1.01533400   -1.69104600   -0.06039900
 C                -0.98924500   -0.66332200   -0.25394600
 C                -0.85900300    0.64892000    0.32974400
 C                 0.69765600    0.76131800   -0.25366600
 H                 1.09654900   -1.86307400   -1.13896000
 H                 1.47609100   -2.51019000    0.48342300
 H                 1.65023600   -0.21995200    1.39078200
 H                -1.19314400   -0.75883300   -1.32239400
```

```
 H                 -0.68552700    0.56910600    1.42227300
 H                  0.67606400    0.71809200   -1.35098500
 O                 -0.38635800   -1.68432500    0.33802700
 O                 -3.13752500   -0.57004200   -0.15419800
 O                 -1.61464600    1.61530300   -0.11832400
 H                 -2.95868800    0.40791700   -0.20038700
 O                  1.12827500    2.01426300    0.17410600
 H                  0.37722100    2.61387800    0.02775100
 O                  2.89147800   -0.27725800   -0.26945100
 H                  3.22652500    0.61866900   -0.15620300
 H                 -3.52290600   -0.73361500    0.71167700
  Core  RigidRotor
           SymmetryFactor   1
    End
Tunneling    Eckart
    ImaginaryFrequency[1/cm]    363.31
    WellDepth[kJ/mol]   299.3
    WellDepth[kJ/mol]   260.1
    End
     Frequencies[1/cm]            53
70.78
106.80
130.55
179.09
235.63
262.17
308.62
330.47
359.48
400.19
407.29
455.23
476.38
497.92
529.84
553.86
574.53
589.20
726.30
873.59
944.76
950.99
1005.83
1019.68
1048.18
1107.83
1118.62
1144.78
1148.45
1169.76
1196.27
1224.53
1250.13
1258.23
1288.61
1319.76
```

```
1325.83
1350.33
1394.33
1414.08
1456.14
1482.04
1584.00
2834.07
2940.67
2964.63
2985.74
3054.45
3100.45
3148.73
3616.65
3755.26
3797.83
     ZeroEnergy[kJ/mol]      299.3
     ElectronicLevels[1/cm]                      1
     0.0000000000000000        1.0000000000000000
     End
  End
```

5.3 Xylopyranose $\longrightarrow$ TH−C−OH2−t + H_2O

```
!****************************************************
!              GLOBAL SECTION
!****************************************************
!!!!!!!!!!!!!!!!!!!!!!!!!!!!!!!!!!!!!!!!!!!!!!!!!!!!
!
!
TemperatureList[K]                      300 320 340 360 380 400 420 440 460 480 500 520 540 560 580 600
620 640 660 680 700 720 740 760 780 800 820 840 860 880 900 920 940 960 980 1000
PressureList[atm]                       1
!
!
EnergyStepOverTemperature               .2         ! [Discretization energy step (global relax matrix)] / T
ExcessEnergyOverTemperature             30         ! [Highest barrier in the model (global relax matrix)] / T
ModelEnergyLimit[kcal/mol]              400        ! Highest reference energy used in the calculation ( or ReferenceEnergy[kcal/mol])
!
CalculationMethod                       direct     ! direct or low-eigenvalue
!
WellCutoff                              20         ! well truncation parameter : Max { dissociation limit (min barrier rel. to bottom of the well) / T }
ChemicalEigenvalueMax                   0.2        ! Max chemical eigenvalue / Lowest Collision relaxation eigenvalue
!
ReductionMethod                         diagonalization ! [low eigenvalue method only] diagonalization or projection (default)
!
!!!!!!!!!test!!!!!!!!!!!!!!!!!!!!
!WellCutoff                            10
!ChemicalEigenvalueMin                 1.e-6          #only for direct diagonalization method
```

```
!!!!!!!!test!!!!!!!!!!!!!!!!!!!!!!!!!
AtomDistanceMin[bohr]                  1.3
!!
RateOutput                             rc2.out                        ! output file name for rate coefficients
!
!
!!!!!!!!!!!!!!!!!!!!!!!!!!!!!!!!!!!!!!!!!!!!!!!!!!!!!!
!*****************************************************
!              MODEL SECTION
!*****************************************************
!!!!!!!!!!!!!!!!!!!!!!!!!!!!!!!!!!!!!!!!!!!!!!!!!!!!!!
!
!
Model
!
  EnergyRelaxation                                                     ! Default collisional energy relaxation kernel
    Exponential                                                        ! Currently the only possible energy relaxation model
       Factor[1/cm]                     260                            ! (Delta_E_down)^(0) @ standard T (300 K)
       Power                            0.875                          ! Power n in the expression (Delta_E_down) = (Delta_E_down)^(0) (T/T0)^(n)
       ExponentCutoff                   10                             ! if (Delta_E) / (Delta_E_down) > value  transition probability is zero
    End
!
  CollisionFrequency                                                   ! Collision frequency model
    LennardJones                                                       ! Currently the only possible collisional frequency model  based on LJ potential
       Epsilons[K]                       90.58  617.0                  ! Epsilon_1 and Epsilon_2 (630.4 x kB x Na = 1.25)(cm-1 to K = x 1.4) Ar and c7h7o2
       Sigmas[angstrom]                  3.54    5.62                  ! Sigma_1 and Sigma_2
       Masses[amu]                       39.948 69.0                   ! Masses of the buffer gas molecule and of the complex (check order)
   End
!
!***************************************************
!
!*****************************************************
!!!!!!!!!!!!!!!!!!!!!!!!!!!!!!!!!!!!!!!!!!!!!!!!!!!!!!
!*****************************************************
!  XILOSIO 1
!*****************************************************
 Well 1
 Species
 RRHO     ! well
 Geometry[angstrom]              20
 C                 -1.38809900   -0.54955600   -0.28755900
 C                 -0.56453100   -1.72483200    0.22988200
 C                  1.43761800   -0.47439000    0.24809800
 C                  0.72705700    0.79121500   -0.21246600
 C                 -0.73922400    0.73445100    0.18821700
 H                 -0.59297400   -1.73232800    1.32855500
 H                 -0.95818500   -2.67540600   -0.13228500
 H                 -1.40210300   -0.56163500   -1.38286400
 H                  1.44588400   -0.51556600    1.34928600
 H                  0.80376600    0.83417500   -1.30627000
 H                 -0.80066100    0.75804700    1.28587100
 O                  0.76641900   -1.62705800   -0.25011700
 O                  2.72158200   -0.47081700   -0.27369400
 H                  3.18204700   -1.24694200    0.06192100
 O                  1.36273200    1.90326300    0.38748700
 H                  0.81522200    2.67245500    0.19230800
```

```
 O                -1.36055300    1.89033900   -0.35564600
 H                -2.29057100    1.85752700   -0.10551800
 O                -2.71496800   -0.55293900    0.23716300
 H                -3.24104700   -1.19396100   -0.24958400
  Core  RigidRotor
  SymmetryFactor    1.0000000000000000
  End
     Frequencies[1/cm]            54
97.25
109.60
231.23
244.30
250.96
257.36
271.96
289.72
326.49
356.54
372.54
399.94
415.21
449.07
515.75
523.37
577.83
635.32
886.53
962.82
992.35
1009.61
1051.19
1063.43
1075.47
1094.19
1104.55
1114.74
1149.92
1163.52
1204.41
1222.85
1232.51
1270.06
1307.70
1309.25
1323.62
1334.25
1354.37
1366.52
1401.57
1409.76
1454.09
1467.04
2885.85
2914.21
2920.19
2946.91
```

```
2963.21
3021.01
3701.33
3709.12
3719.95
3733.18
  ZeroEnergy[kJ/mol]     0.00
  ElectronicLevels[1/cm]                      1
   0.0000000000000000        1.
 End
 End

!***************************************************
!  PRODUCT RC2+H2O
!***************************************************
 Bimolecular PROD
 Fragment C5H8O4
 RRHO
 Geometry[angstrom]               17
 C                 0.43024700   -0.71961100    0.56407400
 C                -0.38000300   -0.65130200   -0.72998500
 C                 1.77039200   -0.00408000    0.43859100
 C                -0.97182800    0.75894300   -0.58937900
 O                -1.38863700   -1.63320500   -0.78288300
 H                 0.24327900   -0.76976600   -1.61678700
 O                -0.39830200   -0.10760600    1.53989900
 H                 0.62201100   -1.75482100    0.85972600
 O                 2.59514000   -0.32526600   -0.37086800
 H                 1.93089200    0.81623200    1.16287300
 C                -1.29967600    0.80844100    0.89870200
 O                 0.02537100    1.74057700   -0.81521800
 H                -1.84697800    0.90155800   -1.22784600
 H                -1.77857600   -1.73065300    0.09187100
 H                -1.14545600    1.81435300    1.29097700
 H                -2.32505100    0.48998900    1.10347000
 H                 0.33651100    1.68276800   -1.72373300
  Core  RigidRotor
  SymmetryFactor    1
  End
     Frequencies[1/cm]            45
83.43
90.93
148.87
215.46
235.81
287.00
304.55
362.24
394.54
428.43
503.75
569.63
745.65
777.92
841.51
868.18
```

```
935.35
957.70
979.39
1029.07
1050.44
1092.82
1111.51
1122.73
1175.68
1194.65
1203.94
1235.37
1265.27
1288.33
1302.45
1333.22
1360.71
1374.70
1397.85
1475.40
1809.61
2900.22
2985.77
3004.35
3009.44
3042.53
3045.33
3756.68
3767.21
  ZeroEnergy[kJ/mol]            0.
  ElectronicLevels[1/cm]                      1
   0.       1
    !************************************
 End
!*********
 Fragment H2O
 RRHO
 Geometry[angstrom]              3
 O                 0.00000000    0.00000000    0.11661200
 H                 0.00000000    0.76156400   -0.46644700
 H                 0.00000000   -0.76156400   -0.46644700
  Core  RigidRotor
  SymmetryFactor    2
  End
     Frequencies[1/cm]           3
1550.43
3775.37
3877.81
  ZeroEnergy[kJ/mol]            0.
  ElectronicLevels[1/cm]                      1
   0.       1
   End
    !************************************
GroundEnergy[kJ/mol]   41.7
End
!************
```

```
! TS-RC2
!************
 Barrier TSRC2 1 PROD
 RRHO            !
 Geometry[angstrom]            20
 C                 1.45883800   -0.81299500    0.35515500
 C                 0.74184700    0.30812000   -0.30030700
 C                -0.58762900    0.78035000    0.18631700
 C                -1.61272600   -0.28135100   -0.22669300
 C                -0.97953100   -1.64425500    0.05296200
 O                 2.66129400   -0.96117900    0.06365600
 O                 1.88230000    1.64522100    0.27882000
 O                -0.85598600    2.04087600   -0.38624600
 O                -2.79606500    0.01767200    0.48778700
 O                 0.27518700   -1.63884800   -0.56815900
 H                 1.08541300   -1.08556500    1.35888500
 H                 0.97859100    0.43871100   -1.34975500
 H                -0.57958200    0.84353600    1.28209000
 H                -1.77060100   -0.17854900   -1.30765300
 H                -0.91150400   -1.79935800    1.14100200
 H                 2.68562700    1.08811500    0.17813200
 H                 1.92127500    2.39603700   -0.32527400
 H                -1.75050400    2.28947200   -0.12990300
 H                -3.53255500   -0.46817900    0.10880800
 H                -1.58479100   -2.45337200   -0.36780300
  Core  RigidRotor
           SymmetryFactor   1
    End
Tunneling    Eckart
    ImaginaryFrequency[1/cm]    553.61
    WellDepth[kJ/mol]   274.7
    WellDepth[kJ/mol]   233.0
    End
     Frequencies[1/cm]            53
96.03
125.56
137.36
216.98
236.09
246.35
280.94
307.09
358.52
365.19
384.25
404.02
428.54
457.89
491.31
544.59
602.61
647.38
663.11
857.26
875.08
1037.14
```

```
1048.35
1060.85
1073.03
1103.91
1111.62
1124.21
1137.07
1152.67
1190.25
1201.16
1227.58
1281.01
1299.72
1303.21
1336.91
1361.21
1394.10
1425.94
1461.62
1509.28
1565.67
2870.02
2911.35
2954.32
2966.86
2991.10
3132.15
3442.63
3756.30
3771.93
3792.12
     ZeroEnergy[kJ/mol]      274.7
     ElectronicLevels[1/cm]                      1
     0.0000000000000000        1.0000000000000000
     End
  End
```

5.4 Xylopyranose $\longrightarrow$ $AXP_1 + H_2O$

```
!***************************************************
!              GLOBAL SECTION
!***************************************************
!!!!!!!!!!!!!!!!!!!!!!!!!!!!!!!!!!!!!!!!!!!!!!!!!!!!
!
!
TemperatureList[K]                      300 320 340 360 380 400 420 440 460 480 500 520 540 560 580 600
620 640 660 680 700 720 740 760 780 800 820 840 860 880 900 920 940 960 980 1000
PressureList[atm]                       1
!
!
```

```
EnergyStepOverTemperature              .2        ! [Discretization energy step (global relax matrix)] / T
ExcessEnergyOverTemperature            30        ! [Highest barrier in the model (global relax matrix)] / T
ModelEnergyLimit[kcal/mol]             400       ! Highest reference energy used in the calculation ( or ReferenceEnergy[kcal/mol])
!
CalculationMethod                      direct    ! direct or low-eigenvalue
!
WellCutoff                             20        ! well truncation parameter : Max { dissociation limit (min barrier rel. to bottom of the well) / T }
ChemicalEigenvalueMax                  0.2       ! Max chemical eigenvalue / Lowest Collision relaxation eigenvalue
!
ReductionMethod                        diagonalization ! [low eigenvalue method only] diagonalization or projection (default)
!
!!!!!!!!!test!!!!!!!!!!!!!!!!!!!
!WellCutoff                            10
!ChemicalEigenvalueMin                 1.e-6          #only for direct diagonalization method
!!!!!!!!test!!!!!!!!!!!!!!!!!!!!!!!!!
AtomDistanceMin[bohr]                  1.3
!!
RateOutput                             d7.out                        ! output file name for rate coefficients
!
!
!!!!!!!!!!!!!!!!!!!!!!!!!!!!!!!!!!!!!!!!!!!!!!!!!!!!!
!***************************************************
!               MODEL SECTION
!***************************************************
!!!!!!!!!!!!!!!!!!!!!!!!!!!!!!!!!!!!!!!!!!!!!!!!!!!!!
!
!
Model
!
  EnergyRelaxation                                                     ! Default collisional energy relaxation kernel
    Exponential                                                        ! Currently the only possible energy relaxation model
       Factor[1/cm]                     260                            ! (Delta_E_down)^(0) @ standard T (300 K)
       Power                            0.875                          ! Power n in the expression (Delta_E_down) = (Delta_E_down)^(0) (T/T0)^(n)
       ExponentCutoff                   10                             ! if (Delta_E) / (Delta_E_down) > value  transition probability is zero
    End
!
  CollisionFrequency                                                   ! Collision frequency model
    LennardJones                                                       ! Currently the only possible collisional frequency model  based on LJ potential
       Epsilons[K]                       90.58  617.0                  ! Epsilon_1 and Epsilon_2 (630.4 x kB x Na = 1.25)(cm-1 to K = x 1.4) Ar and c7h7o2
       Sigmas[angstrom]                  3.54    5.62                  ! Sigma_1 and Sigma_2
       Masses[amu]                       39.948 69.0                   ! Masses of the buffer gas molecule and of the complex (check order)
   End
!
!*************************************************
!
!***************************************************
!!!!!!!!!!!!!!!!!!!!!!!!!!!!!!!!!!!!!!!!!!!!!!!!!!!!!
!***************************************************
!  XILOSIO 1
!***************************************************
 Well 1
 Species
 RRHO     ! well
 Geometry[angstrom]              20
 C                 -1.38809900   -0.54955600   -0.28755900
 C                 -0.56453100   -1.72483200    0.22988200
```

```
C                 1.43761800   -0.47439000    0.24809800
C                 0.72705700    0.79121500   -0.21246600
C                -0.73922400    0.73445100    0.18821700
H                -0.59297400   -1.73232800    1.32855500
H                -0.95818500   -2.67540600   -0.13228500
H                -1.40210300   -0.56163500   -1.38286400
H                 1.44588400   -0.51556600    1.34928600
H                 0.80376600    0.83417500   -1.30627000
H                -0.80066100    0.75804700    1.28587100
O                 0.76641900   -1.62705800   -0.25011700
O                 2.72158200   -0.47081700   -0.27369400
H                 3.18204700   -1.24694200    0.06192100
O                 1.36273200    1.90326300    0.38748700
H                 0.81522200    2.67245500    0.19230800
O                -1.36055300    1.89033900   -0.35564600
H                -2.29057100    1.85752700   -0.10551800
O                -2.71496800   -0.55293900    0.23716300
H                -3.24104700   -1.19396100   -0.24958400
  Core  RigidRotor
  SymmetryFactor    1.0000000000000000
  End
     Frequencies[1/cm]            54
97.25
109.60
231.23
244.30
250.96
257.36
271.96
289.72
326.49
356.54
372.54
399.94
415.21
449.07
515.75
523.37
577.83
635.32
886.53
962.82
992.35
1009.61
1051.19
1063.43
1075.47
1094.19
1104.55
1114.74
1149.92
1163.52
1204.41
1222.85
1232.51
1270.06
```

```
1307.70
1309.25
1323.62
1334.25
1354.37
1366.52
1401.57
1409.76
1454.09
1467.04
2885.85
2914.21
2920.19
2946.91
2963.21
3021.01
3701.33
3709.12
3719.95
3733.18
  ZeroEnergy[kJ/mol]     0.00
  ElectronicLevels[1/cm]                      1
   0.0000000000000000        1.
 End
 End

!***************************************************
!  PRODUCT D7+H2O
!***************************************************
 Bimolecular PROD
 Fragment C5H8O4
 RRHO
 Geometry[angstrom]               17
 C                 -0.42769500   -0.93194800    0.21140800
 C                  1.09433000   -0.82120600    0.09253000
 C                 -1.03695900    0.43646000    0.19650700
 C                  1.42761600    0.25649200   -0.92620300
 O                  1.68503400   -0.53657100    1.34098800
 H                  1.47809900   -1.78650400   -0.24096400
 O                 -0.88730400   -1.72205200   -0.87413400
 H                 -0.66542200   -1.41254900    1.16765500
 C                 -0.33896300    1.53700000   -0.08441200
 O                 -2.37689500    0.41070000    0.49218600
 O                  0.98218300    1.52307900   -0.44418900
 H                  2.50263300    0.35222200   -1.06774300
 H                  0.92969800    0.03175400   -1.87533200
 H                  1.58342700    0.40587100    1.51439300
 H                 -1.84801900   -1.67536800   -0.89229000
 H                 -0.75047000    2.53914000   -0.04032200
 H                 -2.76407000    1.28338800    0.37681900
  Core  RigidRotor
  SymmetryFactor    1
  End
     Frequencies[1/cm]            45
89.11
192.86
```

```
219.63
246.01
274.53
310.27
336.79
348.79
388.85
407.20
458.10
521.23
599.83
642.25
757.69
838.80
840.83
903.79
957.35
985.46
1066.59
1090.86
1107.47
1146.03
1170.20
1186.89
1222.74
1254.10
1280.32
1304.98
1320.02
1353.96
1368.55
1397.02
1418.21
1451.72
1719.48
2969.95
2978.54
3030.84
3069.65
3113.01
3743.71
3770.07
3774.44
  ZeroEnergy[kJ/mol]            0.
  ElectronicLevels[1/cm]                     1
   0.       1
    !***********************************
 End
!*********
 Fragment H2O
 RRHO
 Geometry[angstrom]              3
 O                  0.00000000    0.00000000    0.11661200
 H                  0.00000000    0.76156400   -0.46644700
 H                  0.00000000   -0.76156400   -0.46644700
  Core  RigidRotor
```

```
  SymmetryFactor    2
  End
     Frequencies[1/cm]            3
1550.43
3775.37
3877.81
  ZeroEnergy[kJ/mol]             0.
  ElectronicLevels[1/cm]                     1
   0.        1
   End
    !************************************
GroundEnergy[kJ/mol]   52.7
End
!************
! TS-D7
!************
 Barrier TSD7 1 PROD
 RRHO            !
 Geometry[angstrom]           20
 C                  0.60082500    0.85490700   -0.31073700
 C                  1.30722300   -0.31457800    0.35029200
 C                 -0.87736200    0.57298200   -0.22960800
 C                  0.96573800   -1.60610200   -0.37560500
 O                  2.70803300   -0.19768700    0.27595900
 H                  0.96905200   -0.36421900    1.39247800
 O                  0.96152300    2.01372600    0.41050600
 H                  0.94000900    0.94913500   -1.35322100
 C                 -1.25822800   -0.76453000   -0.60006300
 O                 -1.67146100    1.59613200   -0.78349900
 O                 -0.44661000   -1.76879900   -0.65339600
 H                  1.25079900   -2.47634800    0.21075400
 H                  1.48038600   -1.63260100   -1.33775400
 H                  2.94019000    0.66582700    0.63483800
 H                  0.42148100    2.74278500    0.08926300
 H                 -2.25610600   -0.98653200   -0.95628100
 H                 -2.38401900    1.78236800   -0.16475700
 H                 -1.24998100    0.31234200    0.93448500
 O                 -2.06542100   -0.68555100    1.41827500
 H                 -2.42950500   -1.11140000    2.20176100
   Core  RigidRotor
          SymmetryFactor   1
    End
Tunneling    Eckart
    ImaginaryFrequency[1/cm]    1388.32
    WellDepth[kJ/mol]   281.9
    WellDepth[kJ/mol]   229.2
    End
     Frequencies[1/cm]            53
61.73
83.96
136.83
188.73
212.61
240.64
263.08
303.77
```

```
315.68
367.83
381.82
410.58
420.74
440.48
444.55
507.91
547.13
584.70
726.53
839.30
875.34
906.21
937.16
948.02
1047.57
1069.35
1117.87
1134.64
1160.99
1186.18
1221.72
1232.07
1260.24
1271.07
1281.00
1325.10
1341.96
1352.85
1380.05
1410.51
1417.86
1467.62
1527.65
1730.04
2921.38
2967.72
3016.24
3085.54
3152.21
3750.64
3753.17
3762.89
3764.00
     ZeroEnergy[kJ/mol]      281.9
     ElectronicLevels[1/cm]                      1
     0.0000000000000000        1.0000000000000000
     End
  End
```

5.5 Xylopyranose $\longrightarrow AXP_{2-1} + H_2O$

```
!**************************************************
!              GLOBAL SECTION
!**************************************************
!!!!!!!!!!!!!!!!!!!!!!!!!!!!!!!!!!!!!!!!!!!!!!!!!!!!!
!
!
TemperatureList[K]                        300 320 340 360 380 400 420 440 460 480 500 520 540 560 580 600
620 640 660 680 700 720 740 760 780 800 820 840 860 880 900 920 940 960 980 1000
PressureList[atm]                         1
!
!
EnergyStepOverTemperature                 .2         ! [Discretization energy step (global relax matrix)] / T
ExcessEnergyOverTemperature               30         ! [Highest barrier in the model (global relax matrix)] / T
ModelEnergyLimit[kcal/mol]                400        ! Highest reference energy used in the calculation ( or ReferenceEnergy[kcal/mol])
!
CalculationMethod                         direct     ! direct or low-eigenvalue
!
WellCutoff                                20         ! well truncation parameter : Max { dissociation limit (min barrier rel. to bottom of the well) / T }
ChemicalEigenvalueMax                     0.2        ! Max chemical eigenvalue / Lowest Collision relaxation eigenvalue
!
ReductionMethod                           diagonalization ! [low eigenvalue method only] diagonalization or projection (default)
!
!!!!!!!!!test!!!!!!!!!!!!!!!!!!!
!WellCutoff                              10
!ChemicalEigenvalueMin                   1.e-6          #only for direct diagonalization method
!!!!!!!!test!!!!!!!!!!!!!!!!!!!!!!!!
AtomDistanceMin[bohr]                    1.3
!!
RateOutput                               d6.out                        ! output file name for rate coefficients
!
!
!!!!!!!!!!!!!!!!!!!!!!!!!!!!!!!!!!!!!!!!!!!!!!!!!!!!!
!**************************************************
!              MODEL SECTION
!**************************************************
!!!!!!!!!!!!!!!!!!!!!!!!!!!!!!!!!!!!!!!!!!!!!!!!!!!!!
!
!
Model
!
  EnergyRelaxation                                                       ! Default collisional energy relaxation kernel
    Exponential                                                          ! Currently the only possible energy relaxation model
       Factor[1/cm]                       260                            ! (Delta_E_down)^(0) @ standard T (300 K)
       Power                              0.875                          ! Power n in the expression (Delta_E_down) = (Delta_E_down)^(0) (T/T0)^(n)
       ExponentCutoff                     10                             ! if (Delta_E) / (Delta_E_down) > value  transition probability is zero
    End
!
  CollisionFrequency                                                     ! Collision frequency model
    LennardJones                                                         ! Currently the only possible collisional frequency model  based on LJ potential
       Epsilons[K]                         90.58  617.0                  ! Epsilon_1 and Epsilon_2 (630.4 x kB x Na = 1.25)(cm-1 to K = x 1.4) Ar and c7h7o2
       Sigmas[angstrom]                    3.54    5.62                  ! Sigma_1 and Sigma_2
       Masses[amu]                         39.948 69.0                   ! Masses of the buffer gas molecule and of the complex (check order)
   End
!
```

```
!*************************************************
!
!***************************************************
!!!!!!!!!!!!!!!!!!!!!!!!!!!!!!!!!!!!!!!!!!!!!!!!!!!!
!***************************************************
!  XILOSIO 1
!***************************************************
 Well 1
 Species
 RRHO     ! well
 Geometry[angstrom]               20
 C                 -1.38809900   -0.54955600   -0.28755900
 C                 -0.56453100   -1.72483200    0.22988200
 C                  1.43761800   -0.47439000    0.24809800
 C                  0.72705700    0.79121500   -0.21246600
 C                 -0.73922400    0.73445100    0.18821700
 H                 -0.59297400   -1.73232800    1.32855500
 H                 -0.95818500   -2.67540600   -0.13228500
 H                 -1.40210300   -0.56163500   -1.38286400
 H                  1.44588400   -0.51556600    1.34928600
 H                  0.80376600    0.83417500   -1.30627000
 H                 -0.80066100    0.75804700    1.28587100
 O                  0.76641900   -1.62705800   -0.25011700
 O                  2.72158200   -0.47081700   -0.27369400
 H                  3.18204700   -1.24694200    0.06192100
 O                  1.36273200    1.90326300    0.38748700
 H                  0.81522200    2.67245500    0.19230800
 O                 -1.36055300    1.89033900   -0.35564600
 H                 -2.29057100    1.85752700   -0.10551800
 O                 -2.71496800   -0.55293900    0.23716300
 H                 -3.24104700   -1.19396100   -0.24958400
  Core  RigidRotor
  SymmetryFactor    1.0000000000000000
  End
     Frequencies[1/cm]            54
97.25
109.60
231.23
244.30
250.96
257.36
271.96
289.72
326.49
356.54
372.54
399.94
415.21
449.07
515.75
523.37
577.83
635.32
886.53
962.82
992.35
```

```
1009.61
1051.19
1063.43
1075.47
1094.19
1104.55
1114.74
1149.92
1163.52
1204.41
1222.85
1232.51
1270.06
1307.70
1309.25
1323.62
1334.25
1354.37
1366.52
1401.57
1409.76
1454.09
1467.04
2885.85
2914.21
2920.19
2946.91
2963.21
3021.01
3701.33
3709.12
3719.95
3733.18
  ZeroEnergy[kJ/mol]     0.00
  ElectronicLevels[1/cm]                     1
   0.0000000000000000        1.
 End
 End

!***************************************************
!  PRODUCT D6+H2O
!***************************************************
 Bimolecular PROD
 Fragment C5H8O4
 RRHO
 Geometry[angstrom]              17
 C                -1.32171300    0.24653900   -0.07201500
 C                -0.40459100    1.05997800   -0.61483100
 O                -2.64099800    0.40267200   -0.25917900
 C                 1.05734300    0.79829400   -0.41362300
 H                -0.75191600    1.88941100   -1.21536100
 O                -1.06309200   -0.84130900    0.67545700
 H                -3.09844500   -0.26453400    0.26509000
 C                 1.26352800   -0.66396100   -0.01659300
 O                 1.63668600    1.58027700    0.63254300
 H                 1.60429100    0.98150100   -1.34406800
```

```
C      0.29864300   -1.00117900    1.10779300
O      1.11064500   -1.53217200   -1.11646800
H      2.28374700   -0.78775600    0.34986000
H      1.12854200    2.39035400    0.71830400
H      0.38468300   -2.04380400    1.40553900
H      0.47651300   -0.33850300    1.95840000
H      0.26739600   -1.34043100   -1.54097900
 Core  RigidRotor
 SymmetryFactor    1
 End
   Frequencies[1/cm]          45
100.17
129.85
189.21
217.81
245.03
297.43
318.93
362.07
395.60
450.86
496.56
541.17
624.60
676.01
760.01
805.71
851.66
885.43
928.43
989.04
1035.11
1091.19
1097.44
1115.58
1184.61
1192.08
1215.63
1265.18
1280.56
1309.84
1324.12
1333.04
1368.80
1404.56
1405.03
1460.18
1711.62
2974.08
3000.77
3033.04
3079.40
3140.10
3745.06
3754.54
3791.25
```

```
  ZeroEnergy[kJ/mol]            0.
  ElectronicLevels[1/cm]                      1
   0.       1
    !************************************
 End
!*********
 Fragment H2O
 RRHO
 Geometry[angstrom]              3
 O                 0.00000000    0.00000000    0.11661200
 H                 0.00000000    0.76156400   -0.46644700
 H                 0.00000000   -0.76156400   -0.46644700
  Core  RigidRotor
  SymmetryFactor    2
  End
     Frequencies[1/cm]           3
1550.43
3775.37
3877.81
  ZeroEnergy[kJ/mol]            0.
  ElectronicLevels[1/cm]                      1
   0.       1
   End
    !************************************
GroundEnergy[kJ/mol]   12.6
End
!************
! TS-D6
!************
 Barrier TSD6 1 PROD
 RRHO           !
 Geometry[angstrom]           20
  C                 -0.81665700    0.81866400   -0.00303700
 C                 -1.38136600   -0.58508800   -0.18705000
 C                  0.68312800    0.82234600   -0.24380500
 C                 -0.49799800   -1.58077600    0.54986500
 O                 -2.68911100   -0.66859300    0.32505600
 H                 -1.35901500   -0.84630700   -1.25717500
 O                 -1.49724500    1.75675500   -0.81695000
 H                 -1.01565500    1.12635400    1.02469600
 C                  1.43045600   -0.39222900   -0.08227900
 H                  1.05372000    1.59297800   -0.91551400
 O                  0.76487600   -1.63066400   -0.07140400
 H                 -0.92819800   -2.57929500    0.48962100
 H                 -0.41066200   -1.29144900    1.60688000
 H                 -3.18513200    0.09096700    0.00223000
 H                 -1.35719300    1.52636600   -1.74126800
 O                  2.55478700   -0.47351000   -0.89569700
 H                  3.19403400   -1.04464000   -0.46263600
 H                  1.70140100    0.42677300    1.15350000
 O                  1.33947700    1.54957900    1.29766400
 H                  2.01904900    2.23221400    1.18815000
   Core  RigidRotor
          SymmetryFactor   1
    End
Tunneling    Eckart
```

```
    ImaginaryFrequency[1/cm]    1977.70
    WellDepth[kJ/mol]   345.6
    WellDepth[kJ/mol]   333.0
    End
     Frequencies[1/cm]            53
38.65
90.70
183.79
206.56
250.46
258.96
290.91
309.34
349.64
362.94
380.12
403.44
420.16
450.43
505.01
519.33
528.49
617.16
682.45
775.09
855.49
892.83
1013.45
1026.42
1044.21
1081.86
1105.88
1113.17
1143.92
1165.67
1187.63
1202.96
1232.16
1250.73
1259.83
1284.38
1325.35
1356.35
1373.17
1395.52
1396.54
1441.67
1464.26
1623.56
2905.27
2935.95
3028.56
3059.12
3069.70
3678.56
3750.47
```

```
3753.64
3782.12
     ZeroEnergy[kJ/mol]      345.6
     ElectronicLevels[1/cm]                      1
     0.0000000000000000        1.0000000000000000
     End
  End
```

5.6 Xylopyranose $\longrightarrow$ $AXP_{2-3} + H_2O$

```
!***************************************************
!               GLOBAL SECTION
!***************************************************
!!!!!!!!!!!!!!!!!!!!!!!!!!!!!!!!!!!!!!!!!!!!!!!!!!!
!
!
TemperatureList[K]                      300 320 340 360 380 400 420 440 460 480 500 520 540 560 580 600
620 640 660 680 700 720 740 760 780 800 820 840 860 880 900 920 940 960 980 1000
PressureList[atm]                       1
!
!
EnergyStepOverTemperature               .2         ! [Discretization energy step (global relax matrix)] / T
ExcessEnergyOverTemperature             30         ! [Highest barrier in the model (global relax matrix)] / T
ModelEnergyLimit[kcal/mol]              400        ! Highest reference energy used in the calculation ( or ReferenceEnergy[kcal/mol])
!
CalculationMethod                       direct     ! direct or low-eigenvalue
!
WellCutoff                              20         ! well truncation parameter : Max { dissociation limit (min barrier rel. to bottom of the well) / T }
ChemicalEigenvalueMax                   0.2        ! Max chemical eigenvalue / Lowest Collision relaxation eigenvalue
!
ReductionMethod                         diagonalization ! [low eigenvalue method only] diagonalization or projection (default)
!
!!!!!!!!!test!!!!!!!!!!!!!!!!!!!
!WellCutoff                            10
!ChemicalEigenvalueMin                 1.e-6          #only for direct diagonalization method
!!!!!!!!test!!!!!!!!!!!!!!!!!!!!!!!!!
AtomDistanceMin[bohr]                  1.3
!!
RateOutput                             d5.out                        ! output file name for rate coefficients
!
!
!!!!!!!!!!!!!!!!!!!!!!!!!!!!!!!!!!!!!!!!!!!!!!!!!!!
!***************************************************
!               MODEL SECTION
!***************************************************
!!!!!!!!!!!!!!!!!!!!!!!!!!!!!!!!!!!!!!!!!!!!!!!!!!!
!
!
Model
!
```

```
  EnergyRelaxation                                                          ! Default collisional energy relaxation kernel
    Exponential                                                             ! Currently the only possible energy relaxation model
       Factor[1/cm]                     260                                 ! (Delta_E_down)^(0) @ standard T (300 K)
       Power                            0.875                               ! Power n in the expression (Delta_E_down) = (Delta_E_down)^(0) (T/T0)^(n)
       ExponentCutoff                   10                                  ! if (Delta_E) / (Delta_E_down) > value  transition probability is zero
    End
!
  CollisionFrequency                                                        ! Collision frequency model
    LennardJones                                                            ! Currently the only possible collisional frequency model  based on LJ potential
       Epsilons[K]                       90.58  617.0                       ! Epsilon_1 and Epsilon_2 (630.4 x kB x Na = 1.25)(cm-1 to K = x 1.4) Ar and c7h7o2
       Sigmas[angstrom]                  3.54    5.62                       ! Sigma_1 and Sigma_2
       Masses[amu]                       39.948 69.0                        ! Masses of the buffer gas molecule and of the complex (check order)
   End
!
!**************************************************
!
!****************************************************
!!!!!!!!!!!!!!!!!!!!!!!!!!!!!!!!!!!!!!!!!!!!!!!!!!!!
!****************************************************
!  XILOSIO 1
!****************************************************
 Well 1
 Species
 RRHO     ! well
 Geometry[angstrom]              20
 C                -1.38809900   -0.54955600   -0.28755900
 C                -0.56453100   -1.72483200    0.22988200
 C                 1.43761800   -0.47439000    0.24809800
 C                 0.72705700    0.79121500   -0.21246600
 C                -0.73922400    0.73445100    0.18821700
 H                -0.59297400   -1.73232800    1.32855500
 H                -0.95818500   -2.67540600   -0.13228500
 H                -1.40210300   -0.56163500   -1.38286400
 H                 1.44588400   -0.51556600    1.34928600
 H                 0.80376600    0.83417500   -1.30627000
 H                -0.80066100    0.75804700    1.28587100
 O                 0.76641900   -1.62705800   -0.25011700
 O                 2.72158200   -0.47081700   -0.27369400
 H                 3.18204700   -1.24694200    0.06192100
 O                 1.36273200    1.90326300    0.38748700
 H                 0.81522200    2.67245500    0.19230800
 O                -1.36055300    1.89033900   -0.35564600
 H                -2.29057100    1.85752700   -0.10551800
 O                -2.71496800   -0.55293900    0.23716300
 H                -3.24104700   -1.19396100   -0.24958400
  Core  RigidRotor
  SymmetryFactor    1.0000000000000000
  End
     Frequencies[1/cm]           54
97.25
109.60
231.23
244.30
250.96
257.36
271.96
```

```
289.72
326.49
356.54
372.54
399.94
415.21
449.07
515.75
523.37
577.83
635.32
886.53
962.82
992.35
1009.61
1051.19
1063.43
1075.47
1094.19
1104.55
1114.74
1149.92
1163.52
1204.41
1222.85
1232.51
1270.06
1307.70
1309.25
1323.62
1334.25
1354.37
1366.52
1401.57
1409.76
1454.09
1467.04
2885.85
2914.21
2920.19
2946.91
2963.21
3021.01
3701.33
3709.12
3719.95
3733.18
  ZeroEnergy[kJ/mol]     0.00
  ElectronicLevels[1/cm]                      1
   0.0000000000000000        1.
 End
 End

!***************************************************
!  PRODUCT D5+H2O
!***************************************************
```

```
Bimolecular PROD
Fragment C5H8O4
RRHO
Geometry[angstrom]              17
C                 0.82106800    0.81466400    0.09098500
C                 1.06047300   -0.62562000    0.46243800
C                -0.36628900    1.25205300   -0.31609500
C                -0.26380800   -1.36181800    0.57971500
O                 1.96559000   -1.21854800   -0.45991400
H                 1.57062400   -0.66151000    1.43006800
O                 1.89672400    1.63969100    0.18425700
C                -1.51168700    0.30153900   -0.51106800
H                -0.52419100    2.29622400   -0.55447800
O                -1.10015600   -1.04695800   -0.52135100
H                -0.09648000   -2.43818800    0.55321000
H                -0.75845600   -1.08825000    1.51719800
H                 1.52680000   -1.24671900   -1.31778500
H                 2.69680100    1.10259800    0.17398200
O                -2.46777200    0.53605500    0.49398100
H                -1.95770800    0.45204600   -1.49895500
H                -3.25101100    0.01697300    0.28513400
 Core  RigidRotor
 SymmetryFactor    1
 End
    Frequencies[1/cm]           45
85.73
150.26
195.07
242.75
312.15
346.77
378.06
411.64
424.75
445.03
457.42
554.35
636.86
671.68
763.14
829.98
853.23
905.62
920.92
989.78
1052.20
1074.06
1109.87
1153.02
1193.06
1202.30
1219.19
1232.36
1276.02
1303.57
1350.99
```

```
1375.79
1381.23
1396.87
1414.36
1452.82
1741.14
2978.60
2983.44
2995.13
3056.00
3134.08
3742.34
3745.52
3759.16
  ZeroEnergy[kJ/mol]            0.
  ElectronicLevels[1/cm]                     1
   0.        1
    !************************************
 End
!*********
 Fragment H2O
 RRHO
 Geometry[angstrom]               3
 O                 0.00000000    0.00000000    0.11661200
 H                 0.00000000    0.76156400   -0.46644700
 H                 0.00000000   -0.76156400   -0.46644700
  Core  RigidRotor
  SymmetryFactor    2
  End
     Frequencies[1/cm]           3
1550.43
3775.37
3877.81
  ZeroEnergy[kJ/mol]            0.
  ElectronicLevels[1/cm]                     1
   0.        1
   End
    !************************************
GroundEnergy[kJ/mol]   23.1
End
!************
! TS-D5
!************
 Barrier TSD5 1 PROD
 RRHO           !
 Geometry[angstrom]            20
 C                -0.83666600    0.55052500   -0.31685300
 C                -1.14708500   -0.92565800   -0.30671600
 C                 0.50731100    1.02222800   -0.24986500
 C                 0.11546400   -1.75596600   -0.10416600
 O                -2.17109200   -1.24212400    0.63498300
 H                -1.58031500   -1.19402200   -1.27446700
 O                -1.66293000    1.33770600   -1.15252700
 C                 1.58429500    0.05412900    0.21026300
 H                 0.85183200    1.80814600   -0.91600400
 O                 1.04478900   -1.10846000    0.76590300
```

```
 H                -0.13305500   -2.70437600    0.36952300
 H                 0.59434700   -1.94389100   -1.06929500
 H                -1.88064100   -0.91788000    1.49360000
 H                -2.55734700    1.29155300   -0.80518500
 O                 2.35564700   -0.20947800   -0.93423900
 H                 2.18216200    0.50262700    1.00366000
 H                 3.08606900   -0.77924000   -0.67373800
 H                -0.67425800    1.28646200    0.95407700
 O                 0.26628500    1.98011900    1.24399800
 H                 0.10970000    2.91696700    1.05691200
   Core  RigidRotor
           SymmetryFactor   1
    End
Tunneling    Eckart
    ImaginaryFrequency[1/cm]    1921.14
    WellDepth[kJ/mol]   306.4
    WellDepth[kJ/mol]   283.3
    End
     Frequencies[1/cm]            53
59.96
77.72
171.96
212.04
234.89
283.02
313.66
319.21
328.80
357.64
387.03
409.81
453.96
468.53
520.35
570.88
596.34
663.77
762.33
771.84
859.67
903.82
922.97
973.95
1050.59
1061.87
1081.64
1105.38
1140.53
1145.86
1183.17
1208.09
1218.84
1262.80
1277.12
1320.12
1352.49
```

```
1378.06
1381.73
1394.07
1405.57
1453.60
1461.51
1617.93
2984.37
2998.22
3036.25
3064.66
3090.24
3704.55
3753.22
3767.69
3781.18
     ZeroEnergy[kJ/mol]      306.4
     ElectronicLevels[1/cm]                      1
     0.0000000000000000        1.0000000000000000
     End
  End
```

5.7 Xylopyranose $\longrightarrow$ $AXP_{3-2} + H_2O$

```
!****************************************************
!               GLOBAL SECTION
!****************************************************
!!!!!!!!!!!!!!!!!!!!!!!!!!!!!!!!!!!!!!!!!!!!!!!!!!!!!
!
!
TemperatureList[K]                      300 320 340 360 380 400 420 440 460 480 500 520 540 560 580 600
 620 640 660 680 700 720 740 760 780 800 820 840 860 880 900 920 940 960 980 1000
PressureList[atm]                       1
!
!
EnergyStepOverTemperature               .2         ! [Discretization energy step (global relax matrix)] / T
ExcessEnergyOverTemperature             30         ! [Highest barrier in the model (global relax matrix)] / T
ModelEnergyLimit[kcal/mol]              400        ! Highest reference energy used in the calculation ( or ReferenceEnergy[kcal/mol])
!
CalculationMethod                       direct     ! direct or low-eigenvalue
!
WellCutoff                              20         ! well truncation parameter : Max { dissociation limit (min barrier rel. to bottom of the well) / T }
ChemicalEigenvalueMax                   0.2        ! Max chemical eigenvalue / Lowest Collision relaxation eigenvalue
!
ReductionMethod                         diagonalization ! [low eigenvalue method only] diagonalization or projection (default)
!
!!!!!!!!!test!!!!!!!!!!!!!!!!!!!
!WellCutoff                            10
!ChemicalEigenvalueMin                 1.e-6          #only for direct diagonalization method
!!!!!!!!test!!!!!!!!!!!!!!!!!!!!!!!!!
```

```
AtomDistanceMin[bohr]                  1.3
!!
RateOutput                             d4.out                        ! output file name for rate coefficients
!
!
!!!!!!!!!!!!!!!!!!!!!!!!!!!!!!!!!!!!!!!!!!!!!!!!!!!!
!***************************************************
!              MODEL SECTION
!***************************************************
!!!!!!!!!!!!!!!!!!!!!!!!!!!!!!!!!!!!!!!!!!!!!!!!!!!!
!
!
Model
!
  EnergyRelaxation                                                     ! Default collisional energy relaxation kernel
    Exponential                                                        ! Currently the only possible energy relaxation model
       Factor[1/cm]                    260                             ! (Delta_E_down)^(0) @ standard T (300 K)
       Power                           0.875                           ! Power n in the expression (Delta_E_down) = (Delta_E_down)^(0) (T/T0)^(n)
       ExponentCutoff                  10                              ! if (Delta_E) / (Delta_E_down) > value  transition probability is zero
    End
!
  CollisionFrequency                                                   ! Collision frequency model
    LennardJones                                                       ! Currently the only possible collisional frequency model  based on LJ potential
       Epsilons[K]                      90.58  617.0                   ! Epsilon_1 and Epsilon_2 (630.4 x kB x Na = 1.25)(cm-1 to K = x 1.4) Ar and c7h7o2
       Sigmas[angstrom]                 3.54    5.62                   ! Sigma_1 and Sigma_2
       Masses[amu]                      39.948 69.0                    ! Masses of the buffer gas molecule and of the complex (check order)
   End
!
!*************************************************
!
!***************************************************
!!!!!!!!!!!!!!!!!!!!!!!!!!!!!!!!!!!!!!!!!!!!!!!!!!!!
!***************************************************
!  XILOSIO 1
!***************************************************
 Well 1
 Species
 RRHO     ! well
 Geometry[angstrom]              20
 C                -1.38809900   -0.54955600   -0.28755900
 C                -0.56453100   -1.72483200    0.22988200
 C                 1.43761800   -0.47439000    0.24809800
 C                 0.72705700    0.79121500   -0.21246600
 C                -0.73922400    0.73445100    0.18821700
 H                -0.59297400   -1.73232800    1.32855500
 H                -0.95818500   -2.67540600   -0.13228500
 H                -1.40210300   -0.56163500   -1.38286400
 H                 1.44588400   -0.51556600    1.34928600
 H                 0.80376600    0.83417500   -1.30627000
 H                -0.80066100    0.75804700    1.28587100
 O                 0.76641900   -1.62705800   -0.25011700
 O                 2.72158200   -0.47081700   -0.27369400
 H                 3.18204700   -1.24694200    0.06192100
 O                 1.36273200    1.90326300    0.38748700
 H                 0.81522200    2.67245500    0.19230800
 O                -1.36055300    1.89033900   -0.35564600
```

```
 H                 -2.29057100    1.85752700   -0.10551800
 O                 -2.71496800   -0.55293900    0.23716300
 H                 -3.24104700   -1.19396100   -0.24958400
  Core  RigidRotor
  SymmetryFactor    1.0000000000000000
  End
     Frequencies[1/cm]            54
97.25
109.60
231.23
244.30
250.96
257.36
271.96
289.72
326.49
356.54
372.54
399.94
415.21
449.07
515.75
523.37
577.83
635.32
886.53
962.82
992.35
1009.61
1051.19
1063.43
1075.47
1094.19
1104.55
1114.74
1149.92
1163.52
1204.41
1222.85
1232.51
1270.06
1307.70
1309.25
1323.62
1334.25
1354.37
1366.52
1401.57
1409.76
1454.09
1467.04
2885.85
2914.21
2920.19
2946.91
2963.21
```

```
3021.01
3701.33
3709.12
3719.95
3733.18
  ZeroEnergy[kJ/mol]     0.00
  ElectronicLevels[1/cm]                      1
   0.0000000000000000        1.
 End
 End

!***************************************************
!  PRODUCT D4+H2O
!***************************************************
 Bimolecular PROD
 Fragment C5H8O4
 RRHO
 Geometry[angstrom]               17
 C                  0.74431000    0.84586400    0.02796400
 C                  0.99994100   -0.56930700    0.48525800
 C                 -0.45285500    1.26198500   -0.37048900
 O                  1.99108400   -1.08412100   -0.38032200
 O                 -0.14577200   -1.35526900    0.53053700
 H                  1.37976400   -0.54900400    1.51342700
 O                  1.82249000    1.66796100    0.11692700
 C                 -1.59247700    0.28418000   -0.48790800
 H                 -0.60915100    2.30368700   -0.62405500
 H                  2.27089800   -1.94212500   -0.04651000
 C                 -1.04059900   -1.13151600   -0.55937100
 H                  2.61711900    1.13759600   -0.01526800
 O                 -2.54534200    0.42776500    0.55423400
 H                 -2.14566000    0.48266600   -1.40925900
 H                 -1.84204400   -1.85890800   -0.44226300
 H                 -0.52182000   -1.29568800   -1.50932600
 H                 -2.07870400    0.32385100    1.38953100
  Core  RigidRotor
  SymmetryFactor    1
  End
     Frequencies[1/cm]            45
93.67
150.74
176.78
263.20
325.87
359.51
373.25
404.08
416.68
439.15
490.20
513.17
592.35
672.75
764.38
829.04
859.42
```

```
905.60
950.53
978.22
1061.66
1073.83
1090.89
1149.59
1180.20
1215.78
1220.01
1234.15
1272.23
1313.68
1331.15
1357.43
1377.43
1389.16
1432.35
1456.15
1737.85
2970.58
2978.24
3004.95
3066.49
3124.14
3736.13
3761.34
3768.80
  ZeroEnergy[kJ/mol]            0.
  ElectronicLevels[1/cm]                      1
   0.       1
    !************************************
 End
!*********
 Fragment H2O
 RRHO
 Geometry[angstrom]               3
 O                  0.00000000    0.00000000    0.11661200
 H                  0.00000000    0.76156400   -0.46644700
 H                  0.00000000   -0.76156400   -0.46644700
  Core  RigidRotor
  SymmetryFactor    2
  End
     Frequencies[1/cm]            3
1550.43
3775.37
3877.81
  ZeroEnergy[kJ/mol]            0.
  ElectronicLevels[1/cm]                      1
   0.       1
   End
    !************************************
GroundEnergy[kJ/mol]   25.8
End
!************
! TS-D4
```

```
!************
 Barrier TSD4 1 PROD
 RRHO           !
 Geometry[angstrom]           20
 C                -0.78864900    0.58868900   -0.33239300
 C                -1.06884800   -0.89429900   -0.29434600
 C                 0.54804600    1.07101100   -0.26685400
 O                 0.06729000   -1.68957300   -0.03367500
 O                -2.06699300   -1.11018800    0.67742100
 H                -1.42948100   -1.22551300   -1.27435100
 O                -1.66089700    1.30486100   -1.17655900
 C                 1.64041300    0.08403900    0.09568700
 H                 0.84340500    1.91834500   -0.87862300
 C                 1.01253200   -1.08492100    0.84163100
 H                -2.34488100   -2.03028100    0.62166300
 H                -2.50508900    1.38927300   -0.72671500
 O                 2.25208500   -0.35502100   -1.09864400
 H                 2.40302300    0.57316000    0.69916300
 H                 1.76298400   -1.84516900    1.05445000
 H                 0.53931300   -0.75708800    1.77203800
 H                 1.67284700   -1.02189000   -1.48735400
 H                -0.63938100    1.26367400    0.94333000
 O                 0.33082200    1.88707300    1.34432300
 H                 0.25784200    2.85115500    1.31111400
   Core  RigidRotor
           SymmetryFactor   1
    End
Tunneling    Eckart
    ImaginaryFrequency[1/cm]    1927.86
    WellDepth[kJ/mol]   293.0
    WellDepth[kJ/mol]   267.2
    End
     Frequencies[1/cm]           53
79.16
101.42
167.92
205.69
231.24
284.36
341.39
351.75
384.76
392.24
415.59
423.39
463.89
496.79
504.86
535.69
585.09
658.18
760.81
767.57
853.52
889.64
950.22
```

```
987.20
1052.21
1061.06
1081.82
1091.12
1120.12
1159.83
1181.79
1214.62
1225.73
1255.62
1280.32
1323.41
1341.46
1352.86
1377.42
1383.18
1410.22
1463.18
1471.68
1626.48
2974.24
2988.30
3054.47
3060.98
3095.86
3711.88
3725.27
3758.31
3779.52
     ZeroEnergy[kJ/mol]      293.0
     ElectronicLevels[1/cm]                      1
     0.0000000000000000        1.0000000000000000
     End
  End
```

5.8 Xylopyranose $\longrightarrow$ $AXP_{3-4} + H_2O$

```
!****************************************************
!               GLOBAL SECTION
!****************************************************
!!!!!!!!!!!!!!!!!!!!!!!!!!!!!!!!!!!!!!!!!!!!!!!!!!!!
!
!
TemperatureList[K]                      300 320 340 360 380 400 420 440 460 480 500 520 540 560 580 600
620 640 660 680 700 720 740 760 780 800 820 840 860 880 900 920 940 960 980 1000
PressureList[atm]                       1
!
!
EnergyStepOverTemperature               .2         ! [Discretization energy step (global relax matrix)] / T
```

```
ExcessEnergyOverTemperature            30        ! [Highest barrier in the model (global relax matrix)] / T
ModelEnergyLimit[kcal/mol]             400       ! Highest reference energy used in the calculation ( or ReferenceEnergy[kcal/mol])
!
CalculationMethod                      direct    ! direct or low-eigenvalue
!
WellCutoff                             20        ! well truncation parameter : Max { dissociation limit (min barrier rel. to bottom of the well) / T }
ChemicalEigenvalueMax                  0.2       ! Max chemical eigenvalue / Lowest Collision relaxation eigenvalue
!
ReductionMethod                        diagonalization ! [low eigenvalue method only] diagonalization or projection (default)
!
!!!!!!!!!test!!!!!!!!!!!!!!!!!!!
!WellCutoff                           10
!ChemicalEigenvalueMin                1.e-6          #only for direct diagonalization method
!!!!!!!!test!!!!!!!!!!!!!!!!!!!!!!!!!
AtomDistanceMin[bohr]                 1.3
!!
RateOutput                            d3.out                        ! output file name for rate coefficients
!
!
!!!!!!!!!!!!!!!!!!!!!!!!!!!!!!!!!!!!!!!!!!!!!!!!!!!!!
!****************************************************
!              MODEL SECTION
!****************************************************
!!!!!!!!!!!!!!!!!!!!!!!!!!!!!!!!!!!!!!!!!!!!!!!!!!!!!
!
!
Model
!
  EnergyRelaxation                                                    ! Default collisional energy relaxation kernel
    Exponential                                                       ! Currently the only possible energy relaxation model
       Factor[1/cm]                    260                            ! (Delta_E_down)^(0) @ standard T (300 K)
       Power                           0.875                          ! Power n in the expression (Delta_E_down) = (Delta_E_down)^(0) (T/T0)^(n)
       ExponentCutoff                  10                             ! if (Delta_E) / (Delta_E_down) > value  transition probability is zero
    End
!
  CollisionFrequency                                                  ! Collision frequency model
    LennardJones                                                      ! Currently the only possible collisional frequency model  based on LJ potential
       Epsilons[K]                      90.58  617.0                  ! Epsilon_1 and Epsilon_2 (630.4 x kB x Na = 1.25)(cm-1 to K = x 1.4) Ar and c7h7o2
       Sigmas[angstrom]                 3.54    5.62                  ! Sigma_1 and Sigma_2
       Masses[amu]                      39.948 69.0                   ! Masses of the buffer gas molecule and of the complex (check order)
   End
!
!**************************************************
!
!****************************************************
!!!!!!!!!!!!!!!!!!!!!!!!!!!!!!!!!!!!!!!!!!!!!!!!!!!!!
!****************************************************
!  XILOSIO 1
!****************************************************
 Well 1
 Species
 RRHO    ! well
 Geometry[angstrom]              20
 C                 -1.38809900   -0.54955600   -0.28755900
 C                 -0.56453100   -1.72483200    0.22988200
 C                  1.43761800   -0.47439000    0.24809800
```

```
C                 0.72705700    0.79121500   -0.21246600
C                -0.73922400    0.73445100    0.18821700
H                -0.59297400   -1.73232800    1.32855500
H                -0.95818500   -2.67540600   -0.13228500
H                -1.40210300   -0.56163500   -1.38286400
H                 1.44588400   -0.51556600    1.34928600
H                 0.80376600    0.83417500   -1.30627000
H                -0.80066100    0.75804700    1.28587100
O                 0.76641900   -1.62705800   -0.25011700
O                 2.72158200   -0.47081700   -0.27369400
H                 3.18204700   -1.24694200    0.06192100
O                 1.36273200    1.90326300    0.38748700
H                 0.81522200    2.67245500    0.19230800
O                -1.36055300    1.89033900   -0.35564600
H                -2.29057100    1.85752700   -0.10551800
O                -2.71496800   -0.55293900    0.23716300
H                -3.24104700   -1.19396100   -0.24958400
 Core  RigidRotor
 SymmetryFactor    1.0000000000000000
 End
    Frequencies[1/cm]            54
97.25
109.60
231.23
244.30
250.96
257.36
271.96
289.72
326.49
356.54
372.54
399.94
415.21
449.07
515.75
523.37
577.83
635.32
886.53
962.82
992.35
1009.61
1051.19
1063.43
1075.47
1094.19
1104.55
1114.74
1149.92
1163.52
1204.41
1222.85
1232.51
1270.06
1307.70
```

```
1309.25
1323.62
1334.25
1354.37
1366.52
1401.57
1409.76
1454.09
1467.04
2885.85
2914.21
2920.19
2946.91
2963.21
3021.01
3701.33
3709.12
3719.95
3733.18
  ZeroEnergy[kJ/mol]     0.00
  ElectronicLevels[1/cm]                      1
   0.0000000000000000        1.
 End
 End

!***************************************************
!  PRODUCT D3+H2O
!***************************************************
 Bimolecular PROD
 Fragment C5H8O4
 RRHO
 Geometry[angstrom]               17
  C                  0.97667800    0.87959300   -0.38295000
 C                  1.28919200   -0.56413500    0.00727000
 C                 -0.50192100    1.07375200   -0.55894000
 O                  0.43803300   -0.99670800    1.04600600
 O                  1.16589500   -1.36547600   -1.13360300
 H                  2.29358900   -0.60964900    0.43408200
 O                  1.51921800    1.76542400    0.58454500
 H                  1.50838800    1.09684500   -1.31073100
 C                 -1.36023200    0.18173500   -0.06972900
 H                 -0.84749700    1.96733700   -1.06958100
 C                 -0.92572700   -1.04714200    0.67146000
 H                  1.49354100   -2.24532600   -0.92619900
 H                  1.00123100    1.66265100    1.38961700
 O                 -2.71253600    0.23016200   -0.17696900
 H                 -1.50263500   -1.13587200    1.59310100
 H                 -1.13132900   -1.92624600    0.05016900
 H                 -2.96810100    0.98024000   -0.72295100
  Core  RigidRotor
  SymmetryFactor    1
  End
     Frequencies[1/cm]            45
96.45
143.89
234.28
```

```
251.51
331.80
347.18
367.38
388.97
398.79
470.33
492.35
501.66
533.72
637.13
758.78
821.52
883.56
900.31
960.24
1013.53
1065.07
1070.94
1090.82
1137.67
1166.62
1190.77
1230.39
1238.71
1244.90
1319.31
1325.95
1368.88
1383.07
1415.37
1422.89
1449.52
1715.41
2967.74
3014.07
3025.06
3038.52
3092.51
3754.43
3765.98
3774.72
  ZeroEnergy[kJ/mol]            0.
  ElectronicLevels[1/cm]                     1
   0.       1
    !************************************
 End
!*********
 Fragment H2O
 RRHO
 Geometry[angstrom]              3
 O                 0.00000000    0.00000000    0.11661200
 H                 0.00000000    0.76156400   -0.46644700
 H                 0.00000000   -0.76156400   -0.46644700
  Core  RigidRotor
  SymmetryFactor    2
```

```
  End
     Frequencies[1/cm]            3
1550.43
3775.37
3877.81
  ZeroEnergy[kJ/mol]            0.
  ElectronicLevels[1/cm]                     1
   0.       1
   End
    !************************************
GroundEnergy[kJ/mol]   21.1
End
!************
! TS-D3
!************
 Barrier TSD3 1 PROD
 RRHO           !
 Geometry[angstrom]           20
 C                -1.28323900   -0.24229000   -0.07611200
 C                -0.56929800   -1.10097800   -1.12364100
 C                -0.52614100    0.35399100    0.94970500
 O                 0.81166700   -1.28080100   -0.85122000
 H                -1.01839700   -2.09457300   -1.09773300
 H                -0.68938400   -0.68232400   -2.12700700
 O                -2.58026900   -0.64789000    0.26655600
 C                 0.98136100    0.24292700    0.94881200
 H                -1.01586900    0.62486800    1.87795500
 C                 1.48546600   -0.08931500   -0.45948700
 H                -3.19555900   -0.35460500   -0.40982400
 O                 1.28510600   -0.81664600    1.83090200
 H                 1.44191100    1.14898300    1.33631600
 O                 1.33376400    0.93969500   -1.36653600
 H                 2.53483000   -0.38422200   -0.40930700
 H                 1.14418100   -1.63984100    1.34480100
 H                 0.70640500    1.60410100   -1.01912800
 H                -1.28063300    1.01001100   -0.40938400
 O                -0.63583000    2.14791100    0.01856700
 H                -0.87187900    3.04342500    0.29148500
   Core  RigidRotor
          SymmetryFactor   1
    End
Tunneling    Eckart
    ImaginaryFrequency[1/cm]    1680.06
    WellDepth[kJ/mol]   292.9
    WellDepth[kJ/mol]   271.8
    End
     Frequencies[1/cm]            53
107.56
148.23
199.58
201.57
218.66
271.67
296.23
337.37
358.69
```

```
382.87
426.77
449.53
471.04
486.49
513.31
577.26
611.01
701.99
735.80
781.38
857.79
901.36
945.66
971.30
1004.11
1060.23
1076.54
1111.90
1151.37
1159.91
1204.09
1211.49
1242.58
1267.69
1291.52
1304.96
1328.88
1347.39
1377.04
1388.49
1450.31
1464.31
1518.31
1591.82
2977.88
3027.64
3041.37
3066.14
3129.40
3439.56
3708.83
3730.50
3781.50
     ZeroEnergy[kJ/mol]      292.9
     ElectronicLevels[1/cm]                      1
     0.0000000000000000        1.0000000000000000
     End
  End
```

5.9 Xylopyranose $\longrightarrow AXP_{4-3} + H_2O$

```
!**************************************************
!              GLOBAL SECTION
!**************************************************
!!!!!!!!!!!!!!!!!!!!!!!!!!!!!!!!!!!!!!!!!!!!!!!!!!!!
!
!
TemperatureList[K]                        300 320 340 360 380 400 420 440 460 480 500 520 540 560 580 600
 620 640 660 680 700 720 740 760 780 800 820 840 860 880 900 920 940 960 980 1000
PressureList[atm]                         1
!
!
EnergyStepOverTemperature                 .2          ! [Discretization energy step (global relax matrix)] / T
ExcessEnergyOverTemperature               30          ! [Highest barrier in the model (global relax matrix)] / T
ModelEnergyLimit[kcal/mol]                400         ! Highest reference energy used in the calculation ( or ReferenceEnergy[kcal/mol])
!
CalculationMethod                         direct      ! direct or low-eigenvalue
!
WellCutoff                                20          ! well truncation parameter : Max { dissociation limit (min barrier rel. to bottom of the well) / T }
ChemicalEigenvalueMax                     0.2         ! Max chemical eigenvalue / Lowest Collision relaxation eigenvalue
!
ReductionMethod                           diagonalization ! [low eigenvalue method only] diagonalization or projection (default)
!
!!!!!!!!!test!!!!!!!!!!!!!!!!!!!
!WellCutoff                              10
!ChemicalEigenvalueMin                   1.e-6          #only for direct diagonalization method
!!!!!!!!test!!!!!!!!!!!!!!!!!!!!!!!!
AtomDistanceMin[bohr]                    1.3
!!
RateOutput                               d2.out                        ! output file name for rate coefficients
!
!
!!!!!!!!!!!!!!!!!!!!!!!!!!!!!!!!!!!!!!!!!!!!!!!!!!!!
!**************************************************
!              MODEL SECTION
!**************************************************
!!!!!!!!!!!!!!!!!!!!!!!!!!!!!!!!!!!!!!!!!!!!!!!!!!!!
!
!
Model
!
  EnergyRelaxation                                                       ! Default collisional energy relaxation kernel
    Exponential                                                          ! Currently the only possible energy relaxation model
       Factor[1/cm]                       260                            ! (Delta_E_down)^(0) @ standard T (300 K)
       Power                              0.875                          ! Power n in the expression (Delta_E_down) = (Delta_E_down)^(0) (T/T0)^(n)
       ExponentCutoff                     10                             ! if (Delta_E) / (Delta_E_down) > value  transition probability is zero
    End
!
  CollisionFrequency                                                     ! Collision frequency model
    LennardJones                                                         ! Currently the only possible collisional frequency model  based on LJ potential
       Epsilons[K]                         90.58  617.0                  ! Epsilon_1 and Epsilon_2 (630.4 x kB x Na = 1.25)(cm-1 to K = x 1.4) Ar and c7h7o2
       Sigmas[angstrom]                    3.54    5.62                  ! Sigma_1 and Sigma_2
       Masses[amu]                         39.948 69.0                   ! Masses of the buffer gas molecule and of the complex (check order)
   End
!
```

```
!*************************************************
!
!***************************************************
!!!!!!!!!!!!!!!!!!!!!!!!!!!!!!!!!!!!!!!!!!!!!!!!!!!
!***************************************************
!  XILOSIO 1
!***************************************************
 Well 1
 Species
 RRHO     ! well
 Geometry[angstrom]              20
 C                 -1.38809900   -0.54955600   -0.28755900
 C                 -0.56453100   -1.72483200    0.22988200
 C                  1.43761800   -0.47439000    0.24809800
 C                  0.72705700    0.79121500   -0.21246600
 C                 -0.73922400    0.73445100    0.18821700
 H                 -0.59297400   -1.73232800    1.32855500
 H                 -0.95818500   -2.67540600   -0.13228500
 H                 -1.40210300   -0.56163500   -1.38286400
 H                  1.44588400   -0.51556600    1.34928600
 H                  0.80376600    0.83417500   -1.30627000
 H                 -0.80066100    0.75804700    1.28587100
 O                  0.76641900   -1.62705800   -0.25011700
 O                  2.72158200   -0.47081700   -0.27369400
 H                  3.18204700   -1.24694200    0.06192100
 O                  1.36273200    1.90326300    0.38748700
 H                  0.81522200    2.67245500    0.19230800
 O                 -1.36055300    1.89033900   -0.35564600
 H                 -2.29057100    1.85752700   -0.10551800
 O                 -2.71496800   -0.55293900    0.23716300
 H                 -3.24104700   -1.19396100   -0.24958400
  Core  RigidRotor
  SymmetryFactor    1.0000000000000000
  End
     Frequencies[1/cm]           54
97.25
109.60
231.23
244.30
250.96
257.36
271.96
289.72
326.49
356.54
372.54
399.94
415.21
449.07
515.75
523.37
577.83
635.32
886.53
962.82
992.35
```

```
1009.61
1051.19
1063.43
1075.47
1094.19
1104.55
1114.74
1149.92
1163.52
1204.41
1222.85
1232.51
1270.06
1307.70
1309.25
1323.62
1334.25
1354.37
1366.52
1401.57
1409.76
1454.09
1467.04
2885.85
2914.21
2920.19
2946.91
2963.21
3021.01
3701.33
3709.12
3719.95
3733.18
  ZeroEnergy[kJ/mol]     0.00
  ElectronicLevels[1/cm]                    1
   0.0000000000000000        1.
 End
 End

!***************************************************
!  PRODUCT D2+H2O
!***************************************************
 Bimolecular PROD
 Fragment C5H8O4
 RRHO
 Geometry[angstrom]              17
 C                 0.15470200   -0.77477700   -0.31725100
 C                -1.13666600   -0.22645400    0.27745100
 C                 1.25868100    0.22433900   -0.09144400
 O                -1.38015100    1.03903500   -0.26928200
 O                -2.21815100   -1.05569600   -0.01117900
 H                -1.04546800   -0.18575600    1.37058900
 O                 0.54278000   -1.98531400    0.30475700
 H                 0.00420700   -0.93122700   -1.39556800
 C                 0.98520700    1.50862400    0.11331400
 O                 2.52181300   -0.26564400   -0.13719400
```

```
 C                 -0.42967000    2.01670800    0.13857900
 H                 -2.52020900   -0.83508400   -0.89988500
 H                 -0.17692400   -2.61753800    0.21075800
 H                  1.78887200    2.21516600    0.28326700
 H                  2.47304600   -1.21879500    0.00963700
 H                 -0.55863000    2.85581700   -0.54692900
 H                 -0.68874500    2.36772600    1.14743300
  Core  RigidRotor
  SymmetryFactor    1
  End
     Frequencies[1/cm]            45
93.86
188.53
240.70
259.21
287.18
309.80
326.48
377.57
429.38
454.57
476.60
504.49
532.30
627.68
742.39
806.44
872.49
935.48
983.66
1022.64
1062.61
1119.59
1154.37
1158.82
1176.17
1207.40
1212.90
1241.23
1270.47
1303.42
1344.25
1350.44
1384.97
1393.66
1415.06
1467.69
1746.22
2924.55
2926.41
2964.61
3020.00
3123.34
3719.01
3743.97
3764.75
```

```
  ZeroEnergy[kJ/mol]            0.
  ElectronicLevels[1/cm]                      1
   0.        1
    !************************************
 End
!*********
 Fragment H2O
 RRHO
 Geometry[angstrom]              3
 O                 0.00000000    0.00000000    0.11661200
 H                 0.00000000    0.76156400   -0.46644700
 H                 0.00000000   -0.76156400   -0.46644700
  Core  RigidRotor
  SymmetryFactor    2
  End
     Frequencies[1/cm]           3
1550.43
3775.37
3877.81
  ZeroEnergy[kJ/mol]            0.
  ElectronicLevels[1/cm]                      1
   0.        1
   End
    !************************************
GroundEnergy[kJ/mol]   24.2
End
!************
! TS-D2
!************
 Barrier TSD2 1 PROD
 RRHO           !
 Geometry[angstrom]           20
 C                 0.94218400   -0.56312100   -0.62225800
 C                -0.33092800   -1.40187700   -0.44015400
 C                 0.70713600    0.84647300   -0.12556400
 O                -0.64320300   -1.36595300    0.95030200
 O                -1.38690000   -1.03071300   -1.24994500
 H                -0.10802700   -2.44771600   -0.64835500
 O                 2.02155100   -1.15665400    0.08767800
 H                 1.23749300   -0.55257000   -1.67243300
 C                -0.29600400    1.04099600    0.85944000
 O                 1.83042000    1.67463600   -0.11757100
 C                -1.12494600   -0.10929200    1.37950800
 H                -1.57837200   -0.07740400   -1.18237000
 H                 1.67287200   -1.45270400    0.93778100
 H                -0.26952900    1.95167800    1.44713400
 H                 2.56979100    1.13350700    0.18585100
 H                -2.17761400    0.01678600    1.12283100
 H                -1.03380000   -0.10527300    2.46956400
 H                -0.21763000    1.43131000   -0.85125100
 O                -1.49354700    1.72413900   -0.59359900
 H                -2.10639000    2.45967500   -0.72950600
  Core  RigidRotor
          SymmetryFactor   1
    End
Tunneling    Eckart
```

```
    ImaginaryFrequency[1/cm]    1770.34
    WellDepth[kJ/mol]   294.5
    WellDepth[kJ/mol]   270.3
    End
     Frequencies[1/cm]            53
115.93
138.97
200.72
224.65
250.67
273.21
312.49
346.97
387.56
394.98
417.90
457.29
472.24
495.84
582.61
608.43
648.09
665.28
739.53
806.30
817.65
904.07
915.86
949.91
1018.98
1052.22
1066.16
1118.49
1147.91
1166.97
1203.32
1210.84
1238.31
1249.39
1285.58
1299.76
1335.72
1358.95
1375.44
1385.05
1435.05
1442.35
1479.42
1597.14
2988.29
3031.90
3036.60
3051.95
3124.14
3528.84
3707.80
```

```
3715.17
3720.06
     ZeroEnergy[kJ/mol]      294.5
     ElectronicLevels[1/cm]                    1
     0.0000000000000000        1.0000000000000000
     End
  End
```

5.10 Xylopyranose $\longrightarrow$ $AXP_{4-5} + H_2O$

```
!***************************************************
!               GLOBAL SECTION
!***************************************************
!!!!!!!!!!!!!!!!!!!!!!!!!!!!!!!!!!!!!!!!!!!!!!!!!!!!
!
!
TemperatureList[K]                      300 320 340 360 380 400 420 440 460 480 500 520 540 560 580 600
620 640 660 680 700 720 740 760 780 800 820 840 860 880 900 920 940 960 980 1000
PressureList[atm]                       1
!
!
EnergyStepOverTemperature               .2         ! [Discretization energy step (global relax matrix)] / T
ExcessEnergyOverTemperature             30         ! [Highest barrier in the model (global relax matrix)] / T
ModelEnergyLimit[kcal/mol]              400        ! Highest reference energy used in the calculation ( or ReferenceEnergy[kcal/mol])
!
CalculationMethod                       direct     ! direct or low-eigenvalue
!
WellCutoff                              20         ! well truncation parameter : Max { dissociation limit (min barrier rel. to bottom of the well) / T }
ChemicalEigenvalueMax                   0.2        ! Max chemical eigenvalue / Lowest Collision relaxation eigenvalue
!
ReductionMethod                         diagonalization ! [low eigenvalue method only] diagonalization or projection (default)
!
!!!!!!!!!test!!!!!!!!!!!!!!!!!!!
!WellCutoff                            10
!ChemicalEigenvalueMin                 1.e-6          #only for direct diagonalization method
!!!!!!!!test!!!!!!!!!!!!!!!!!!!!!!!!!
AtomDistanceMin[bohr]                  1.3
!!
RateOutput                             d1.out                       ! output file name for rate coefficients
!
!
!!!!!!!!!!!!!!!!!!!!!!!!!!!!!!!!!!!!!!!!!!!!!!!!!!!!
!***************************************************
!               MODEL SECTION
!***************************************************
!!!!!!!!!!!!!!!!!!!!!!!!!!!!!!!!!!!!!!!!!!!!!!!!!!!!
!
!
Model
!
```

```
  EnergyRelaxation                                                              ! Default collisional energy relaxation kernel
    Exponential                                                                 ! Currently the only possible energy relaxation model
       Factor[1/cm]                     260                                     ! (Delta_E_down)^(0) @ standard T (300 K)
       Power                            0.875                                   ! Power n in the expression (Delta_E_down) = (Delta_E_down)^(0) (T/T0)^(n)
       ExponentCutoff                   10                                      ! if (Delta_E) / (Delta_E_down) > value  transition probability is zero
    End
!
  CollisionFrequency                                                            ! Collision frequency model
    LennardJones                                                                ! Currently the only possible collisional frequency model  based on LJ potential
       Epsilons[K]                       90.58  617.0                           ! Epsilon_1 and Epsilon_2 (630.4 x kB x Na = 1.25)(cm-1 to K = x 1.4) Ar and c7h7o2
       Sigmas[angstrom]                  3.54    5.62                           ! Sigma_1 and Sigma_2
       Masses[amu]                       39.948 69.0                            ! Masses of the buffer gas molecule and of the complex (check order)
   End
!
!**************************************************
!
!****************************************************
!!!!!!!!!!!!!!!!!!!!!!!!!!!!!!!!!!!!!!!!!!!!!!!!!!!!
!****************************************************
!  XILOSIO 1
!****************************************************
 Well 1
 Species
 RRHO     ! well
 Geometry[angstrom]              20
 C                -1.38809900   -0.54955600   -0.28755900
 C                -0.56453100   -1.72483200    0.22988200
 C                 1.43761800   -0.47439000    0.24809800
 C                 0.72705700    0.79121500   -0.21246600
 C                -0.73922400    0.73445100    0.18821700
 H                -0.59297400   -1.73232800    1.32855500
 H                -0.95818500   -2.67540600   -0.13228500
 H                -1.40210300   -0.56163500   -1.38286400
 H                 1.44588400   -0.51556600    1.34928600
 H                 0.80376600    0.83417500   -1.30627000
 H                -0.80066100    0.75804700    1.28587100
 O                 0.76641900   -1.62705800   -0.25011700
 O                 2.72158200   -0.47081700   -0.27369400
 H                 3.18204700   -1.24694200    0.06192100
 O                 1.36273200    1.90326300    0.38748700
 H                 0.81522200    2.67245500    0.19230800
 O                -1.36055300    1.89033900   -0.35564600
 H                -2.29057100    1.85752700   -0.10551800
 O                -2.71496800   -0.55293900    0.23716300
 H                -3.24104700   -1.19396100   -0.24958400
  Core  RigidRotor
  SymmetryFactor    1.0000000000000000
  End
     Frequencies[1/cm]           54
97.25
109.60
231.23
244.30
250.96
257.36
271.96
```

```
289.72
326.49
356.54
372.54
399.94
415.21
449.07
515.75
523.37
577.83
635.32
886.53
962.82
992.35
1009.61
1051.19
1063.43
1075.47
1094.19
1104.55
1114.74
1149.92
1163.52
1204.41
1222.85
1232.51
1270.06
1307.70
1309.25
1323.62
1334.25
1354.37
1366.52
1401.57
1409.76
1454.09
1467.04
2885.85
2914.21
2920.19
2946.91
2963.21
3021.01
3701.33
3709.12
3719.95
3733.18
  ZeroEnergy[kJ/mol]     0.00
  ElectronicLevels[1/cm]                      1
   0.0000000000000000        1.
 End
 End

!***************************************************
!  PRODUCT D1+H2O
!***************************************************
```

```
Bimolecular PROD
Fragment C5H8O4
RRHO
Geometry[angstrom]              17
C                -0.58674700   -0.04496300    1.50676100
C                 0.72310600   -0.26833200    1.58337500
C                 0.99278500   -0.21668000   -0.75847100
C                -0.19198500    0.74448200   -0.80940400
C                -1.25941800    0.31137700    0.20630600
H                 1.24334700   -0.50214500    2.50345700
H                -1.18351100   -0.13011600    2.40559900
H                 1.78958500    0.09974200   -1.43371600
H                -0.62270300    0.72803700   -1.81085500
H                -1.91878500    1.16929900    0.35580800
O                 1.59260700   -0.21203400    0.52686900
O                 0.51413900   -1.49555600   -1.07333900
H                 1.24735500   -2.11786600   -1.05240000
O                 0.26026500    2.05730300   -0.56885600
H                 0.64021800    2.09428600    0.31596300
O                -2.08541900   -0.72663200   -0.28306300
H                -1.51467800   -1.48120100   -0.46813900
 Core  RigidRotor
 SymmetryFactor    1
 End
    Frequencies[1/cm]           45
130.31
169.71
234.20
255.24
314.65
333.67
339.14
372.92
461.30
478.68
523.91
601.21
621.73
633.80
782.18
817.78
882.44
940.27
959.42
985.93
1055.07
1088.43
1094.77
1104.75
1110.97
1185.64
1218.44
1231.98
1268.33
1298.54
1318.62
```

```
1352.19
1363.53
1392.06
1409.97
1426.37
1676.56
3008.37
3024.24
3042.18
3125.17
3143.94
3740.06
3745.02
3771.31
  ZeroEnergy[kJ/mol]            0.
  ElectronicLevels[1/cm]                      1
   0.       1
    !************************************
 End
!*********
 Fragment H2O
 RRHO
 Geometry[angstrom]               3
 O                 0.00000000    0.00000000    0.11661200
 H                 0.00000000    0.76156400   -0.46644700
 H                 0.00000000   -0.76156400   -0.46644700
  Core  RigidRotor
  SymmetryFactor    2
  End
     Frequencies[1/cm]           3
1550.43
3775.37
3877.81
  ZeroEnergy[kJ/mol]            0.
  ElectronicLevels[1/cm]                      1
   0.       1
   End
    !************************************
GroundEnergy[kJ/mol]   21.7
End
!************
! TS-D1
!************
 Barrier TSD1 1 PROD
 RRHO           !
 Geometry[angstrom]            20
 C                 0.40255100    0.27980300    1.13438700
 C                -0.17784900    1.24292200    0.09149000
 C                 1.14389000   -0.86234300    0.42668100
 C                -0.83465700    0.45738400   -1.02295900
 O                 0.79459600    2.06944600   -0.51225900
 H                -0.90499100    1.89447400    0.57275000
 O                -0.58260700   -0.23603200    1.98479100
 H                 1.12831700    0.82780000    1.73997400
 O                 2.20614700   -0.27124900   -0.29831400
 O                 0.31730400   -1.60994400   -0.42165800
```

```
 H                 1.51477700   -1.58097900    1.15910200
 C                -0.51287700   -0.88438000   -1.29585600
 H                -1.31616500    1.03006900   -1.80617000
 H                 1.56219900    1.51876300   -0.71730600
 H                -1.39482600   -0.38036000    1.46755400
 H                 2.68673100   -0.96402000   -0.76146700
 H                -0.45846500   -1.22952200   -2.32252300
 H                -1.74869200   -0.99414300   -0.85423900
 O                -2.46266100   -0.16095600   -0.06186300
 H                -3.37746400    0.14748700   -0.00570500
  Core  RigidRotor
           SymmetryFactor   1
    End
Tunneling    Eckart
    ImaginaryFrequency[1/cm]    1748.18
    WellDepth[kJ/mol]   281.6
    WellDepth[kJ/mol]   259.9
    End
     Frequencies[1/cm]            53
118.17
152.91
203.93
209.78
253.13
271.66
318.42
343.81
368.07
420.24
442.36
465.83
541.33
552.10
596.86
608.97
616.18
656.19
796.41
810.41
829.30
883.61
912.65
967.63
993.73
1044.43
1081.28
1083.58
1096.42
1129.88
1181.58
1212.84
1236.43
1257.51
1291.44
1309.04
1311.31
```

```
1337.97
1377.79
1403.95
1411.75
1446.43
1474.42
1594.01
3012.23
3027.91
3052.40
3097.94
3134.74
3544.10
3690.47
3708.84
3771.71
     ZeroEnergy[kJ/mol]      281.6
     ElectronicLevels[1/cm]                      1
     0.0000000000000000        1.0000000000000000
     End
  End
```

6 Geometries of stationary points

6.1 Xylopyranose decompositions

6.1.1 Ring opening

Xylopyranose

```
0 1
 C    -1.38315700   -0.55311700   -0.29076300
 C    -0.55952400   -1.72670300    0.23383900
 C     1.44159700   -0.46690700    0.25020300
 C     0.72625100    0.79532500   -0.21250600
 C    -0.73908100    0.73151100    0.19091500
 H    -0.59924600   -1.73198400    1.33267900
 H    -0.94607800   -2.67940500   -0.13073300
```

H	-1.38574100	-0.56202900	-1.38734200
H	1.45715600	-0.49706600	1.35260400
H	0.80100300	0.83544200	-1.30756000
H	-0.79856600	0.74964500	1.28998200
O	0.76988300	-1.62118800	-0.23075300
O	2.71618000	-0.46676300	-0.28026000
H	3.16424200	-1.26710100	0.00864200
O	1.35010000	1.90569800	0.38384200
H	0.81527900	2.67796400	0.17189000
O	-1.36555400	1.87940900	-0.34383800
H	-2.29659200	1.84373300	-0.10187300
O	-2.70700100	-0.55343100	0.22025200
H	-3.23683600	-1.19965000	-0.25235700

Xylose

0 1

C	-0.84707700	-0.92723300	-0.01342800
C	-2.31335000	-0.52233800	0.12679500
C	1.82690300	0.63350200	-0.62247100
C	1.52996900	-0.31227600	0.53247600
C	0.05984000	-0.21522600	0.99974100
H	-2.93694600	-1.21619500	-0.43833800
H	-2.61093000	-0.56076800	1.18008400
H	-0.75969100	-2.00429100	0.15615300
H	2.31714800	0.16858500	-1.49751400
H	2.17747500	-0.00770100	1.36064700
H	-0.02491200	-0.74318000	1.95476500

O	-2.53128200	0.75744200	-0.43292200
O	1.58714900	1.81456700	-0.58966500
H	-2.08409900	1.38511000	0.14834600
O	1.88136100	-1.63096100	0.19937600
H	1.42708100	-1.86093100	-0.62064500
O	-0.33601000	1.12714500	1.23456100
H	0.23687400	1.72321500	0.72888300
O	-0.39612000	-0.67822300	-1.33953800
H	-0.92048900	0.05782500	-1.68555400

TS-Xylose

0 1

O	-2.55104400	0.02081100	-0.11237300
O	0.42262000	2.04978100	-0.09199000
O	-0.50633500	-1.00624400	1.62008400
O	2.36281200	0.17529300	-0.28462900
O	0.76857800	-0.96914200	-1.12418200
C	-0.39269400	1.03930800	0.44548100
C	0.42638700	-0.02835300	1.17464200
C	1.46894700	-0.64698300	0.24869300
C	-0.65807000	-0.83318100	-1.31975600
C	-1.29264900	0.39569400	-0.63305200
H	-2.38785600	-0.54789600	0.64969400
H	1.32375000	1.70346900	-0.20731300
H	-0.09474200	-1.59325900	2.25922800
H	1.55763600	-0.19746700	-1.35350800

H	-1.07699600	1.49803300	1.16526900
H	0.96779900	0.42853300	2.01015200
H	1.82440500	-1.62662900	0.59801000
H	-1.49074500	1.17743900	-1.36656400
H	-0.80617500	-0.82648200	-2.39833500
H	-1.10165400	-1.73863700	-0.90795700

6.1.2 Dehydrations

AXP_1

0 1

C	-0.42769500	-0.93194800	0.21140800
C	1.09433000	-0.82120600	0.09253000
C	-1.03695900	0.43646000	0.19650700
C	1.42761600	0.25649200	-0.92620300
O	1.68503400	-0.53657100	1.34098800
H	1.47809900	-1.78650400	-0.24096400
O	-0.88730400	-1.72205200	-0.87413400
H	-0.66542200	-1.41254900	1.16765500
C	-0.33896300	1.53700000	-0.08441200
O	-2.37689500	0.41070000	0.49218600
O	0.98218300	1.52307900	-0.44418900
H	2.50263300	0.35222200	-1.06774300
H	0.92969800	0.03175400	-1.87533200
H	1.58342700	0.40587100	1.51439300
H	-1.84801900	-1.67536800	-0.89229000
H	-0.75047000	2.53914000	-0.04032200

H	-2.76407000	1.28338800	0.37681900

AXP_{2-1}

0 1

C	-1.32171300	0.24653900	-0.07201500
C	-0.40459100	1.05997800	-0.61483100
O	-2.64099800	0.40267200	-0.25917900
C	1.05734300	0.79829400	-0.41362300
H	-0.75191600	1.88941100	-1.21536100
O	-1.06309200	-0.84130900	0.67545700
H	-3.09844500	-0.26453400	0.26509000
C	1.26352800	-0.66396100	-0.01659300
O	1.63668600	1.58027700	0.63254300
H	1.60429100	0.98150100	-1.34406800
C	0.29864300	-1.00117900	1.10779300
O	1.11064500	-1.53217200	-1.11646800
H	2.28374700	-0.78775600	0.34986000
H	1.12854200	2.39035400	0.71830400
H	0.38468300	-2.04380400	1.40553900
H	0.47651300	-0.33850300	1.95840000
H	0.26739600	-1.34043100	-1.54097900

AXP_{2-3}

0 1

C	0.82106800	0.81466400	0.09098500

C	1.06047300	-0.62562000	0.46243800
C	-0.36628900	1.25205300	-0.31609500
C	-0.26380800	-1.36181800	0.57971500
O	1.96559000	-1.21854800	-0.45991400
H	1.57062400	-0.66151000	1.43006800
O	1.89672400	1.63969100	0.18425700
C	-1.51168700	0.30153900	-0.51106800
H	-0.52419100	2.29622400	-0.55447800
O	-1.10015600	-1.04695800	-0.52135100
H	-0.09648000	-2.43818800	0.55321000
H	-0.75845600	-1.08825000	1.51719800
H	1.52680000	-1.24671900	-1.31778500
H	2.69680100	1.10259800	0.17398200
O	-2.46777200	0.53605500	0.49398100
H	-1.95770800	0.45204600	-1.49895500
H	-3.25101100	0.01697300	0.28513400

AXP_{3-2}

0 1

C	0.74431000	0.84586400	0.02796400
C	0.99994100	-0.56930700	0.48525800
C	-0.45285500	1.26198500	-0.37048900
O	1.99108400	-1.08412100	-0.38032200
O	-0.14577200	-1.35526900	0.53053700
H	1.37976400	-0.54900400	1.51342700
O	1.82249000	1.66796100	0.11692700

C	-1.59247700	0.28418000	-0.48790800
H	-0.60915100	2.30368700	-0.62405500
H	2.27089800	-1.94212500	-0.04651000
C	-1.04059900	-1.13151600	-0.55937100
H	2.61711900	1.13759600	-0.01526800
O	-2.54534200	0.42776500	0.55423400
H	-2.14566000	0.48266600	-1.40925900
H	-1.84204400	-1.85890800	-0.44226300
H	-0.52182000	-1.29568800	-1.50932600
H	-2.07870400	0.32385100	1.38953100

AXP_{3-4}

0 1

C	0.97667800	0.87959300	-0.38295000
C	1.28919200	-0.56413500	0.00727000
C	-0.50192100	1.07375200	-0.55894000
O	0.43803300	-0.99670800	1.04600600
O	1.16589500	-1.36547600	-1.13360300
H	2.29358900	-0.60964900	0.43408200
O	1.51921800	1.76542400	0.58454500
H	1.50838800	1.09684500	-1.31073100
C	-1.36023200	0.18173500	-0.06972900
H	-0.84749700	1.96733700	-1.06958100
C	-0.92572700	-1.04714200	0.67146000
H	1.49354100	-2.24532600	-0.92619900
H	1.00123100	1.66265100	1.38961700
O	-2.71253600	0.23016200	-0.17696900

H	-1.50263500	-1.13587200	1.59310100
H	-1.13132900	-1.92624600	0.05016900
H	-2.96810100	0.98024000	-0.72295100

$\mathbf{AXP_{4-3}}$

0 1

C	0.15470200	-0.77477700	-0.31725100
C	-1.13666600	-0.22645400	0.27745100
C	1.25868100	0.22433900	-0.09144400
O	-1.38015100	1.03903500	-0.26928200
O	-2.21815100	-1.05569600	-0.01117900
H	-1.04546800	-0.18575600	1.37058900
O	0.54278000	-1.98531400	0.30475700
H	0.00420700	-0.93122700	-1.39556800
C	0.98520700	1.50862400	0.11331400
O	2.52181300	-0.26564400	-0.13719400
C	-0.42967000	2.01670800	0.13857900
H	-2.52020900	-0.83508400	-0.89988500
H	-0.17692400	-2.61753800	0.21075800
H	1.78887200	2.21516600	0.28326700
H	2.47304600	-1.21879500	0.00963700
H	-0.55863000	2.85581700	-0.54692900
H	-0.68874500	2.36772600	1.14743300

$\mathbf{AXP_{4-5}}$

0 1

C	-0.58674700	-0.04496300	1.50676100
C	0.72310600	-0.26833200	1.58337500
C	0.99278500	-0.21668000	-0.75847100
C	-0.19198500	0.74448200	-0.80940400
C	-1.25941800	0.31137700	0.20630600
H	1.24334700	-0.50214500	2.50345700
H	-1.18351100	-0.13011600	2.40559900
H	1.78958500	0.09974200	-1.43371600
H	-0.62270300	0.72803700	-1.81085500
H	-1.91878500	1.16929900	0.35580800
O	1.59260700	-0.21203400	0.52686900
O	0.51413900	-1.49555600	-1.07333900
H	1.24735500	-2.11786600	-1.05240000
O	0.26026500	2.05730300	-0.56885600
H	0.64021800	2.09428600	0.31596300
O	-2.08541900	-0.72663200	-0.28306300
H	-1.51467800	-1.48120100	-0.46813900

H_2O

0 1

O	0.00000000	0.00000000	0.11661200
H	0.00000000	0.76156400	-0.46644700
H	0.00000000	-0.76156400	-0.46644700

TS−AXP_1

0 1

C	0.60082500	0.85490700	-0.31073700
C	1.30722300	-0.31457800	0.35029200
C	-0.87736200	0.57298200	-0.22960800
C	0.96573800	-1.60610200	-0.37560500
O	2.70803300	-0.19768700	0.27595900
H	0.96905200	-0.36421900	1.39247800
O	0.96152300	2.01372600	0.41050600
H	0.94000900	0.94913500	-1.35322100
C	-1.25822800	-0.76453000	-0.60006300
O	-1.67146100	1.59613200	-0.78349900
O	-0.44661000	-1.76879900	-0.65339600
H	1.25079900	-2.47634800	0.21075400
H	1.48038600	-1.63260100	-1.33775400
H	2.94019000	0.66582700	0.63483800
H	0.42148100	2.74278500	0.08926300
H	-2.25610600	-0.98653200	-0.95628100
H	-2.38401900	1.78236800	-0.16475700
H	-1.24998100	0.31234200	0.93448500
O	-2.06542100	-0.68555100	1.41827500
H	-2.42950500	-1.11140000	2.20176100

TS−AXP$_{2-1}$

0 1

C	-0.81665700	0.81866400	-0.00303700
C	-1.38136600	-0.58508800	-0.18705000
C	0.68312800	0.82234600	-0.24380500

C	-0.49799800	-1.58077600	0.54986500
O	-2.68911100	-0.66859300	0.32505600
H	-1.35901500	-0.84630700	-1.25717500
O	-1.49724500	1.75675500	-0.81695000
H	-1.01565500	1.12635400	1.02469600
C	1.43045600	-0.39222900	-0.08227900
H	1.05372000	1.59297800	-0.91551400
O	0.76487600	-1.63066400	-0.07140400
H	-0.92819800	-2.57929500	0.48962100
H	-0.41066200	-1.29144900	1.60688000
H	-3.18513200	0.09096700	0.00223000
H	-1.35719300	1.52636600	-1.74126800
O	2.55478700	-0.47351000	-0.89569700
H	3.19403400	-1.04464000	-0.46263600
H	1.70140100	0.42677300	1.15350000
O	1.33947700	1.54957900	1.29766400
H	2.01904900	2.23221400	1.18815000

TS−AXP$_{2-3}$

0 1

C	-0.83666600	0.55052500	-0.31685300
C	-1.14708500	-0.92565800	-0.30671600
C	0.50731100	1.02222800	-0.24986500
C	0.11546400	-1.75596600	-0.10416600
O	-2.17109200	-1.24212400	0.63498300
H	-1.58031500	-1.19402200	-1.27446700

O	-1.66293000	1.33770600	-1.15252700
C	1.58429500	0.05412900	0.21026300
H	0.85183200	1.80814600	-0.91600400
O	1.04478900	-1.10846000	0.76590300
H	-0.13305500	-2.70437600	0.36952300
H	0.59434700	-1.94389100	-1.06929500
H	-1.88064100	-0.91788000	1.49360000
H	-2.55734700	1.29155300	-0.80518500
O	2.35564700	-0.20947800	-0.93423900
H	2.18216200	0.50262700	1.00366000
H	3.08606900	-0.77924000	-0.67373800
H	-0.67425800	1.28646200	0.95407700
O	0.26628500	1.98011900	1.24399800
H	0.10970000	2.91696700	1.05691200

TS−AXP$_{3-2}$

0 1

C	-0.78864900	0.58868900	-0.33239300
C	-1.06884800	-0.89429900	-0.29434600
C	0.54804600	1.07101100	-0.26685400
O	0.06729000	-1.68957300	-0.03367500
O	-2.06699300	-1.11018800	0.67742100
H	-1.42948100	-1.22551300	-1.27435100
O	-1.66089700	1.30486100	-1.17655900
C	1.64041300	0.08403900	0.09568700
H	0.84340500	1.91834500	-0.87862300

C	1.01253200	-1.08492100	0.84163100
H	-2.34488100	-2.03028100	0.62166300
H	-2.50508900	1.38927300	-0.72671500
O	2.25208500	-0.35502100	-1.09864400
H	2.40302300	0.57316000	0.69916300
H	1.76298400	-1.84516900	1.05445000
H	0.53931300	-0.75708800	1.77203800
H	1.67284700	-1.02189000	-1.48735400
H	-0.63938100	1.26367400	0.94333000
O	0.33082200	1.88707300	1.34432300
H	0.25784200	2.85115500	1.31111400

TS−AXP$_{3-4}$

0 1

C	-1.28323900	-0.24229000	-0.07611200
C	-0.56929800	-1.10097800	-1.12364100
C	-0.52614100	0.35399100	0.94970500
O	0.81166700	-1.28080100	-0.85122000
H	-1.01839700	-2.09457300	-1.09773300
H	-0.68938400	-0.68232400	-2.12700700
O	-2.58026900	-0.64789000	0.26655600
C	0.98136100	0.24292700	0.94881200
H	-1.01586900	0.62486800	1.87795500
C	1.48546600	-0.08931500	-0.45948700
H	-3.19555900	-0.35460500	-0.40982400
O	1.28510600	-0.81664600	1.83090200

H	1.44191100	1.14898300	1.33631600
O	1.33376400	0.93969500	-1.36653600
H	2.53483000	-0.38422200	-0.40930700
H	1.14418100	-1.63984100	1.34480100
H	0.70640500	1.60410100	-1.01912800
H	-1.28063300	1.01001100	-0.40938400
O	-0.63583000	2.14791100	0.01856700
H	-0.87187900	3.04342500	0.29148500

TS−AXP$_{4-3}$

0 1

C	0.94218400	-0.56312100	-0.62225800
C	-0.33092800	-1.40187700	-0.44015400
C	0.70713600	0.84647300	-0.12556400
O	-0.64320300	-1.36595300	0.95030200
O	-1.38690000	-1.03071300	-1.24994500
H	-0.10802700	-2.44771600	-0.64835500
O	2.02155100	-1.15665400	0.08767800
H	1.23749300	-0.55257000	-1.67243300
C	-0.29600400	1.04099600	0.85944000
O	1.83042000	1.67463600	-0.11757100
C	-1.12494600	-0.10929200	1.37950800
H	-1.57837200	-0.07740400	-1.18237000
H	1.67287200	-1.45270400	0.93778100
H	-0.26952900	1.95167800	1.44713400
H	2.56979100	1.13350700	0.18585100

H	-2.17761400	0.01678600	1.12283100
H	-1.03380000	-0.10527300	2.46956400
H	-0.21763000	1.43131000	-0.85125100
O	-1.49354700	1.72413900	-0.59359900
H	-2.10639000	2.45967500	-0.72950600

TS−AXP$_{4-5}$

0 1

C	0.40255100	0.27980300	1.13438700
C	-0.17784900	1.24292200	0.09149000
C	1.14389000	-0.86234300	0.42668100
C	-0.83465700	0.45738400	-1.02295900
O	0.79459600	2.06944600	-0.51225900
H	-0.90499100	1.89447400	0.57275000
O	-0.58260700	-0.23603200	1.98479100
H	1.12831700	0.82780000	1.73997400
O	2.20614700	-0.27124900	-0.29831400
O	0.31730400	-1.60994400	-0.42165800
H	1.51477700	-1.58097900	1.15910200
C	-0.51287700	-0.88438000	-1.29585600
H	-1.31616500	1.03006900	-1.80617000
H	1.56219900	1.51876300	-0.71730600
H	-1.39482600	-0.38036000	1.46755400
H	2.68673100	-0.96402000	-0.76146700
H	-0.45846500	-1.22952200	-2.32252300
H	-1.74869200	-0.99414300	-0.85423900

```
 O                   -2.46266100   -0.16095600   -0.06186300
 H                   -3.37746400    0.14748700   -0.00570500
```

6.1.3 Ring contractions

TC-C-OH2-c

```
0 1
 C                    0.07653500    0.84888000   -0.20689300
 C                    1.51995000    0.47578100    0.13927800
 C                   -0.50161500   -0.54454200   -0.49716800
 C                    1.32245600   -0.81377000    0.92016500
 O                    2.25302500    0.16422800   -1.03059600
 H                    1.99851000    1.25776200    0.73441200
 O                   -0.47958500    1.44990000    0.93777100
 H                    0.01550300    1.50494100   -1.08124600
 C                   -1.98446400   -0.66025600   -0.23084400
 O                    0.19033900   -1.46089000    0.33277900
 H                   -0.32617100   -0.79731700   -1.55320100
 H                    2.18906100   -1.46864600    0.82991700
 H                    1.11282200   -0.59768000    1.97040000
 H                    2.40916800    0.97129400   -1.52789600
 H                   -1.41202500    1.62125600    0.76584200
 O                   -2.74550300    0.26294800   -0.36250800
 H                   -2.33024400   -1.65765300    0.09496800
```

TC-C-OH2-t

```
0 1
 C                    0.43024700   -0.71961100    0.56407400
```

C	-0.38000300	-0.65130200	-0.72998500
C	1.77039200	-0.00408000	0.43859100
C	-0.97182800	0.75894300	-0.58937900
O	-1.38863700	-1.63320500	-0.78288300
H	0.24327900	-0.76976600	-1.61678700
O	-0.39830200	-0.10760600	1.53989900
H	0.62201100	-1.75482100	0.85972600
O	2.59514000	-0.32526600	-0.37086800
H	1.93089200	0.81623200	1.16287300
C	-1.29967600	0.80844100	0.89870200
O	0.02537100	1.74057700	-0.81521800
H	-1.84697800	0.90155800	-1.22784600
H	-1.77857600	-1.73065300	0.09187100
H	-1.14545600	1.81435300	1.29097700
H	-2.32505100	0.48998900	1.10347000
H	0.33651100	1.68276800	-1.72373300

TS-TC-C-OH2-c

0 1

C	1.60322100	-0.32679000	0.30022400
C	1.01533400	-1.69104600	-0.06039900
C	-0.98924500	-0.66332200	-0.25394600
C	-0.85900300	0.64892000	0.32974400
C	0.69765600	0.76131800	-0.25366600
H	1.09654900	-1.86307400	-1.13896000
H	1.47609100	-2.51019000	0.48342300

H	1.65023600	-0.21995200	1.39078200
H	-1.19314400	-0.75883300	-1.32239400
H	-0.68552700	0.56910600	1.42227300
H	0.67606400	0.71809200	-1.35098500
O	-0.38635800	-1.68432500	0.33802700
O	-3.13752500	-0.57004200	-0.15419800
O	-1.61464600	1.61530300	-0.11832400
H	-2.95868800	0.40791700	-0.20038700
O	1.12827500	2.01426300	0.17410600
H	0.37722100	2.61387800	0.02775100
O	2.89147800	-0.27725800	-0.26945100
H	3.22652500	0.61866900	-0.15620300
H	-3.52290600	-0.73361500	0.71167700

TS-TC-C-OH2-t

0 1

C	1.45883800	-0.81299500	0.35515500
C	0.74184700	0.30812000	-0.30030700
C	-0.58762900	0.78035000	0.18631700
C	-1.61272600	-0.28135100	-0.22669300
C	-0.97953100	-1.64425500	0.05296200
O	2.66129400	-0.96117900	0.06365600
O	1.88230000	1.64522100	0.27882000
O	-0.85598600	2.04087600	-0.38624600
O	-2.79606500	0.01767200	0.48778700
O	0.27518700	-1.63884800	-0.56815900
H	1.08541300	-1.08556500	1.35888500

H	0.97859100	0.43871100	-1.34975500
H	-0.57958200	0.84353600	1.28209000
H	-1.77060100	-0.17854900	-1.30765300
H	-0.91150400	-1.79935800	1.14100200
H	2.68562700	1.08811500	0.17813200
H	1.92127500	2.39603700	-0.32527400
H	-1.75050400	2.28947200	-0.12990300
H	-3.53255500	-0.46817900	0.10880800
H	-1.58479100	-2.45337200	-0.36780300

6.2 Xylose decompositions

6.2.1 Dehydration

A1

0 1

C	1.07374400	-0.23077000	-0.38595400
C	1.74260800	0.52215700	0.76711700
C	-1.76563400	0.93129300	-0.40731800
C	-1.42411800	-0.42702100	0.09693900
C	-0.17798100	-0.91750900	0.07879000
H	1.10271800	1.34320500	1.10691500
H	1.90154400	-0.16622800	1.59966700
H	0.86775200	0.48090600	-1.19626400
H	-0.93045400	1.48629300	-0.86888500
H	0.00456500	-1.91602700	0.47200600
O	3.02728500	0.97807700	0.37508600

O	-2.86152600	1.41182900	-0.32740700
H	2.93829500	1.79292300	-0.12584000
O	-2.50583800	-1.08454800	0.57176000
H	-2.24917600	-1.96195600	0.87083100
O	1.94501900	-1.23851600	-0.85676400
H	2.83351700	-0.86275000	-0.85728500

A2

0 1

C	-1.39063900	0.08367200	0.71633200
C	-1.09929000	1.43157500	0.06403800
C	1.95332300	0.10304100	-0.25257700
C	0.79177800	-0.86619500	-0.00789000
C	-0.09948700	-0.61495500	1.17993800
H	-2.04294600	1.94925100	-0.13413700
H	-0.48534700	2.05271000	0.71936200
H	-2.02821700	0.24043900	1.59189500
H	2.42921500	-0.00561400	-1.24253900
H	-0.35660300	-1.57848300	1.62507800
O	-0.36528900	1.25151800	-1.13449500
O	2.31662400	0.88376400	0.57887900
H	-0.92716800	0.75260400	-1.73847400
O	0.61183700	-1.77400400	-0.78432200
H	0.41160500	0.00772300	1.91478600
O	-2.10927700	-0.71005100	-0.21374700
H	-1.56580700	-1.45127900	-0.50553000

A2-a1

0 1

C	1.00249400	-0.59809700	0.80577900
C	2.00128200	-0.04694900	-0.20531000
C	-0.93108000	0.70034200	0.63293800
C	-1.18388800	-0.61237200	-0.14137600
C	-0.02130300	-1.53685500	0.16839100
H	2.53650700	0.79360300	0.25145900
H	2.72784000	-0.83050300	-0.43480700
H	1.54388900	-1.05259400	1.63598300
H	-1.75874400	0.93724300	1.30518000
H	-0.36616500	-2.29023700	0.88136500
O	1.40385000	0.31245000	-1.42934800
O	-0.75202600	1.72997300	-0.31039500
H	0.81591000	1.06579000	-1.28626000
O	-2.14058100	-0.82509100	-0.82214700
H	0.32033100	-2.02577600	-0.74225900
O	0.20738100	0.45630400	1.39715200
H	-0.77358500	2.57698000	0.14470200

A2-a2

0 1

C	-0.92153400	0.94382500	0.19736200
C	-1.52800300	-0.05724300	-0.79430300

C	0.09391200	-0.88082400	0.55766600
C	1.20626800	-0.00328500	-0.04310000
C	0.48622800	1.33193600	-0.27625100
H	-2.57555600	-0.26920900	-0.57578500
H	-1.41071200	0.24431800	-1.83631000
H	-1.57103600	1.74977200	0.52406500
H	0.38372100	-1.75243100	1.13425600
H	0.92442700	2.09809900	0.36561800
O	-0.74321800	-1.23089700	-0.53512100
O	2.34015800	-0.29274200	-0.27946700
H	0.55971000	1.64557900	-1.31748600
O	-0.63841300	0.05831600	1.29676300

A2-b1

0 1

C	0.00000000	0.50947200	0.00000000
C	-0.71407300	1.63550800	0.00000000
C	-0.67145700	-0.81193000	0.00000000
H	-1.79415100	1.57738800	0.00000000
H	-0.24084700	2.60801400	0.00000000
H	-1.77357800	-0.80643900	0.00000000
O	-0.02963400	-1.83275900	0.00000000
O	1.34425800	0.46866100	0.00000000
H	1.60476500	-0.46447600	0.00000000

HAA

0 1

C	0.66684700	0.64812100	0.00006400
H	0.93666900	1.24427600	0.88256300
C	-0.83057400	0.48311700	0.00001300
H	-1.43161800	1.41039000	0.00008800
O	1.33375100	-0.57601400	0.00003700
H	0.93669200	1.24435300	-0.88238100
O	-1.34870200	-0.60116900	-0.00010800
H	0.66022900	-1.26898500	-0.00016000

MGO

0 1

C	0.00000000	0.55500000	0.00000000
O	-0.58488900	1.60619900	0.00000000
C	1.48981000	0.38817400	0.00000000
H	1.96932200	1.36407800	0.00000000
H	1.79273600	-0.19073800	0.87548200
H	1.79273600	-0.19073800	-0.87548200
C	-0.84342900	-0.72906700	0.00000000
H	-1.93468200	-0.55101900	0.00000000
O	-0.35241000	-1.82072800	0.00000000

A2-c1

0 1

C	-1.74821400	0.11154200	0.49433600
C	-1.35272000	1.20800600	-0.50235300
C	1.98173400	0.39374600	0.40046900

C	0.55731100	-0.01384100	0.03634500
C	-0.41278100	-0.14198700	1.20707200
H	-1.88075400	1.08630200	-1.44856000
H	-1.53219600	2.20970600	-0.10461600
H	-2.52718000	0.45067200	1.17633500
H	2.06846800	1.29793300	1.02844000
H	-0.21574300	0.64946100	1.93535500
O	0.06205200	1.06573200	-0.72076600
O	2.93592100	-0.20516400	-0.00518300
H	1.40009300	-1.29144500	-1.14709600
O	0.55696900	-1.21001900	-0.68139200
H	-0.36669200	-1.11746300	1.68700200
O	-2.25018600	-1.04743800	-0.13079300
H	-1.53601700	-1.45484700	-0.63700200

A2-c2

0 1

C	-2.07089000	0.40597000	-0.30239400
C	-1.80532100	-0.84687900	0.03818900
C	1.43159600	-0.17233400	-0.76899900
C	0.23792900	0.12730100	0.13779700
C	-0.76506100	1.14942300	-0.43181300
H	-2.46458600	-1.68408000	0.21233200
H	-3.05106700	0.82511100	-0.46273400
H	1.18271200	-0.42004600	-1.81618800
H	-0.54278200	1.40137700	-1.47401800

O	-0.47055700	-1.12548500	0.18799100
O	2.55501600	-0.17487100	-0.35524600
H	1.52290400	0.12684000	1.55538200
O	0.65233800	0.51376800	1.39063800
H	-0.71107500	2.06261500	0.16147800

A2-c3

0 1

C	-1.33911400	1.14126200	0.23268800
C	-1.51662800	-0.37482400	0.43563300
C	-0.07490100	-0.81535900	0.49971000
C	0.67682300	0.18938000	0.04350000
C	2.14076700	0.22488900	-0.20667400
H	-2.08656100	-0.58679100	1.33970900
H	-2.03703300	1.54898900	-0.49679500
H	2.52142500	1.18209300	-0.60496600
H	0.28279400	-1.79917300	0.76084900
O	0.00361200	1.33454100	-0.24946100
O	2.85899500	-0.71316100	0.00662500
O	-2.22717900	-1.01629200	-0.61005700
H	-1.65967700	-1.05383800	-1.38639600
H	-1.42605000	1.67592600	1.18160500

FF

0 1

C	0.00000000	0.28292200	0.00000000
C	-0.95583200	-0.68892200	0.00000000
C	-0.06686500	1.74305500	0.00000000
C	-0.25635000	-1.92961800	0.00000000
H	-2.01994400	-0.51674900	0.00000000
O	1.23922900	-0.27898200	0.00000000
O	-1.10390200	2.35642600	0.00000000
H	0.91526200	2.25023000	0.00000000
C	1.06737700	-1.61144900	0.00000000
H	-0.67366700	-2.92295100	0.00000000
H	1.96575500	-2.20600900	0.00000000

TS-A

0 1

C	-1.17826900	0.20567100	-0.26499100
C	-1.50313500	-1.07585400	0.50378100
C	1.66491400	-0.87563300	-0.73284500
C	1.39660300	0.22971100	0.13283700
C	0.07021300	0.84584700	0.28430400
H	-0.74326000	-1.84157900	0.32966700
H	-1.54299900	-0.85724000	1.57331900
H	-1.04109400	-0.02764600	-1.32962700
H	0.97286900	-1.02301700	-1.58366200
H	1.32641800	1.60948700	-0.70063000
H	-0.09455400	1.22996300	1.29107100
O	-2.80002200	-1.52107100	0.13687200
O	2.64517200	-1.59708200	-0.59189800

H	-2.73767800	-2.07701600	-0.64377700
O	2.20024500	0.32543400	1.27100000
H	2.86980200	-0.36617900	1.16722100
O	0.31435600	2.12222000	-0.62513300
H	0.24674900	2.97707500	-0.16847600
O	-2.22308000	1.13925000	-0.10461500
H	-3.05158300	0.64769500	-0.16342900

TS-A1A2

0 1

C	-1.08810100	0.31477100	-0.29293400
C	-1.75421300	-0.73846000	0.59079800
C	1.93022500	-0.97472500	-0.34326600
C	1.49633900	0.41547600	0.05075500
C	0.17695900	0.85355500	0.32001300
H	-1.10019200	-1.60935200	0.71417600
H	-1.94613100	-0.30478000	1.57474000
H	-0.86085900	-0.14087300	-1.27082700
H	1.15739200	-1.57173300	-0.86405300
H	0.07583600	1.20798900	1.34979300
O	-3.02081400	-1.10458500	0.06569500
O	3.00807400	-1.41725000	-0.07525600
H	-2.90047100	-1.73218200	-0.65149300
O	2.32557800	1.36505100	0.17737000
H	1.14102700	1.94338700	0.20865600
O	-1.97272300	1.40127900	-0.47098700
H	-2.85477500	1.02787600	-0.58776500

TS-A2a1

0 1

C	-0.76752100	-0.51230300	-0.29628600
C	-2.07192700	-0.45272700	0.47607600
C	1.29866600	0.94834500	-0.31228300
C	1.61795000	-0.52907200	-0.06374000
C	0.36670900	-1.20494700	0.46560900
H	-2.35179600	-1.47874500	0.74843900
H	-1.90912300	0.10643800	1.40142300
H	-0.92205900	-0.99021800	-1.26791700
H	1.73601600	1.37454200	-1.22218300
H	0.40467100	-2.28177000	0.31481000
O	-3.10144600	0.21105500	-0.21253200
O	1.16246500	1.68751700	0.75984900
H	-3.33502000	-0.28619100	-1.00059800
O	2.68460200	-1.03209700	-0.26491400
H	0.29736300	-0.98871100	1.53724900
O	-0.30414700	0.83101500	-0.59065600
H	-0.11510800	1.46896400	0.33855300

TS-A2a12

0 1

C	-1.47280400	-0.42141100	0.40608200
C	-0.97765300	-1.38936500	-0.69387000
C	0.56678000	0.31178500	1.02503800
C	0.11685600	1.22922700	-0.10316900

C	-1.38638700	0.98925200	-0.14965100
H	-0.80015800	-2.37698300	-0.23872100
H	-1.80612700	-1.49370800	-1.41374700
H	-2.36988500	-0.70460000	0.94882900
H	1.42244400	0.42420800	1.67406000
H	-1.88207600	1.71317200	0.50400200
O	0.12872700	-0.79755900	-1.21812500
O	2.18216100	-1.00999700	0.12226800
H	1.43043900	-1.08498400	-0.57408200
O	0.79198000	2.07561400	-0.61011700
H	-1.75558400	1.07637300	-1.16772500
O	-0.38421500	-0.42591000	1.41687700
H	2.93097500	-0.60758900	-0.32642200

TS-A2b1

0 1

C	-1.29032900	-0.58478400	0.32534800
C	-1.42065700	0.92810200	0.23718600
C	1.96903000	0.80325200	0.35442000
C	1.21373000	-0.50679900	0.23363300
C	0.44781200	-0.94637000	1.30367900
H	-1.29219400	1.39402600	1.21530700
H	-0.67089100	1.32041900	-0.46122400
H	-1.86749300	-1.04763200	1.13444100
H	1.94455700	1.26390400	1.36104100
H	0.20259200	-1.99852400	1.36561600
O	-2.72910500	1.23068800	-0.19162900

O	2.56130000	1.29541200	-0.55866500
H	-2.85727100	0.83007400	-1.05786700
O	1.16484000	-1.06906800	-0.91310200
H	0.49168800	-0.39946700	2.23868000
O	-1.18917100	-1.23518800	-0.77015300
H	0.06859000	-1.29796500	-1.05319300

TS-MGO

0 1

C	0.40194300	0.00908800	-0.05509700
C	1.59557700	-0.73791500	0.08589000
C	-1.00438800	-0.52930100	-0.16431800
H	1.70766500	-1.74351000	-0.30246900
H	2.07621400	-0.56512100	1.04883000
H	-1.07472100	-1.55711400	-0.56446100
O	-1.96057800	0.09039400	0.19761200
O	0.64954100	1.25066200	-0.09637100
H	1.82034400	0.68606600	-0.19067300

TS-A2c1

0 1

C	-1.35353100	0.45375900	-0.14862900
C	-1.22218400	-0.92010200	-0.80534200
C	1.90154000	0.59504400	-0.32468100
C	0.89576700	-0.29312200	0.41115000
C	-0.31935300	0.41366800	0.98338600

H	-1.70445300	-1.67162000	-0.17478600
H	-1.63064300	-0.95625900	-1.81371600
H	-1.07426500	1.22830500	-0.87284700
H	2.78177500	0.04513300	-0.70370600
H	-0.05107800	1.41336800	1.32702400
O	0.19231100	-1.13974600	-0.85594500
O	1.75225100	1.77549400	-0.46307100
H	0.68370000	-1.84568700	-0.07148300
O	1.38189600	-1.37597400	0.95639800
H	-0.70655600	-0.18161800	1.81196800
O	-2.68453400	0.60825200	0.27601800
H	-2.84730600	1.52869100	0.49505000

TS-A2c12

0 1

C	-1.39462200	-0.91641700	-0.01805200
C	-1.22824900	-0.22781000	1.23070800
C	2.12967000	0.10094400	0.11481400
C	0.62041900	0.30706500	-0.00946900
C	-0.11490100	-0.77530600	-0.79679300
H	-2.23244300	0.58551100	0.37986900
H	-1.52695300	-0.67207400	2.17133100
H	-1.99962100	-1.80938400	-0.13298700
H	2.64846900	0.91144600	0.66436700
H	0.44225200	-1.71399600	-0.73262700
O	0.12059600	0.27088500	1.29307900
O	2.72007900	-0.83807200	-0.33506700

H	0.46750300	2.22978500	0.06797200
O	0.38328400	1.55639500	-0.61620800
H	-0.24801000	-0.49982700	-1.84027900
O	-2.50084900	0.27114600	-0.70306400
H	-3.40998500	-0.04515500	-0.81482000

TS-A2c13

0 1

C	1.61011500	-0.15943400	0.66366900
C	1.45018600	-1.27873900	-0.37651600
C	-2.06967900	-0.26752300	-0.25842000
C	-0.60081800	-0.46157400	0.00728900
C	0.15262600	0.13036700	1.06143500
H	2.07975700	-1.12593200	-1.24937800
H	1.56598600	-2.28612900	0.02461100
H	2.22728100	-0.49298100	1.49700100
H	-2.36164900	-0.34544200	-1.31594600
H	-0.22003500	0.05506500	2.07742600
O	0.05817200	-1.18149600	-0.82881000
O	-2.82940800	-0.07115600	0.64393200
H	-0.66694700	2.46798500	-1.22953000
O	-0.37233600	1.69083600	-0.74582200
H	-0.10241700	1.23932600	0.61406700
O	2.22802000	0.97023400	0.10699300
H	1.54786600	1.44217700	-0.41333000

TS-A2c2FF

0 1

C	1.94519600	-0.42879600	-0.50360700
C	1.88112300	-0.14623300	0.79956800
C	0.46729300	0.07617500	1.13984900
C	-0.19594800	-0.17580600	-0.11394400
H	2.72413200	-0.04125100	1.46309800
H	0.19203900	1.30684400	0.84188000
O	0.66886200	-0.50548000	-1.05933000
H	2.75031200	-0.61957500	-1.19152200
H	0.00482700	-0.19947800	2.07876100
C	-1.64046300	-0.45383700	-0.38157300
H	-1.92262700	-0.42366600	-1.44706800
O	-0.51588100	1.74668300	-0.24738100
H	-0.43688600	2.41566700	-0.94120000
O	-2.40985600	-0.69964800	0.50099800

TS-A2c3FF

0 1

C	-1.32501000	1.01024400	0.24657200
C	-1.30946200	-0.31663900	0.82914900
C	0.01579400	-0.80004600	0.70714800
C	0.71633200	0.19408700	0.08830200
C	2.13545000	0.22131900	-0.34901800
H	-2.09094300	-0.73456200	1.44140200
H	-1.97430000	0.56355900	-0.72331500
H	2.44499600	1.14064000	-0.87515600
H	0.41038000	-1.75986600	0.99573200

O	0.00470800	1.28884000	-0.15127100
O	2.88571700	-0.69021200	-0.13621600
O	-2.34374900	-0.78200500	-0.87139000
H	-2.76347300	-1.45978400	-1.41866500
H	-1.79870200	1.86324300	0.71810300

6.2.2 Cyclization

B1

0 1

O	-0.30263900	0.91312200	-0.65684300
O	0.16756800	-0.86079400	1.74325800
O	1.88667900	-0.89703000	-1.42542700
O	1.13543300	1.76002700	0.96576600
O	-3.03666200	0.29300000	-0.58613100
C	0.36370400	-1.11440700	0.35570100
C	-0.75166900	-0.43476100	-0.43581900
C	1.59020500	-0.35841500	-0.15659600
C	1.03583100	1.07466000	-0.24312900
C	-2.10803300	-0.37690700	0.23961600
H	0.42291300	-2.18427400	0.14425200
H	-0.84698300	-0.91958100	-1.41143800
H	2.44300300	-0.41455000	0.52804600
H	1.55466500	1.68573300	-0.98504700
H	-2.01205900	0.11564300	1.21162100
H	-2.48919700	-1.38788500	0.39846600
H	0.81019700	-1.36669000	2.24787200

H	2.52423500	-0.33411400	-1.87222700
H	0.67576800	1.23827000	1.63639100
H	-2.66579400	1.15983700	-0.78155300

ADX

0 1

O	-0.30733500	-1.07952800	1.05145200
O	-1.17243900	-0.85908900	-1.01389500
O	0.03153300	2.16595300	-0.29433200
O	2.33038100	-0.52902700	-0.06962900
C	-0.79638400	0.26364400	0.94784500
C	0.43494300	0.99786000	0.38003900
C	1.05717800	-0.11893600	-0.51191300
C	0.00559900	-1.21702400	-0.32504900
C	-1.82724800	0.10209900	-0.16948100
H	-1.16951900	0.61754300	1.90412300
H	1.13625100	1.22960200	1.18921200
H	1.15027200	0.19411200	-1.55118000
H	0.30831500	-2.23285400	-0.56613900
H	-2.76055400	-0.32292900	0.20353000
H	-2.00819200	1.02284800	-0.72044000
H	0.81101600	2.64315500	-0.58882400
H	2.23077400	-0.90381200	0.81228600

TS-B

0 1

C	-0.74496000	-0.50759400	-0.23070600
C	-2.11328800	-0.24869900	0.36483500
C	1.30247800	0.92262600	-0.56140000
C	1.64379700	-0.43904900	0.02354300
C	0.36502100	-0.84261200	0.77854500
H	-2.40536000	-1.11420300	0.96165400
H	-2.06283300	0.61545800	1.03604500
H	-0.83065700	-1.29453500	-0.98475500
H	1.77077700	1.15088100	-1.52633700
H	2.48521400	-0.34736200	0.71103400
H	0.37225400	-1.91134100	0.99603000
O	-3.09515100	-0.08487200	-0.63406400
O	1.04126500	1.91445800	0.26796300
H	-2.87262400	0.68781600	-1.16101000
O	1.99981500	-1.36981800	-0.97151100
H	1.38595600	-1.31190000	-1.71028300
O	0.23147000	-0.15869900	2.00021200
H	0.51165500	0.75885100	1.86226600
O	-0.29244000	0.68366500	-0.93918900
H	-0.15234900	1.58043500	-0.22083000

TS-B1ADX

0 1

O	0.32720200	0.36647100	1.48556800
O	-1.31007200	-1.56689300	-1.00615800
O	-1.99000700	1.73288100	-0.31079400

O	2.35529400	1.39017300	-0.55442200
O	1.23199000	-0.86297000	-0.81075200
C	-1.26397700	-0.65352500	0.03765200
C	-0.14655900	-0.96989400	1.07231100
C	-0.93775300	0.79521700	-0.41093500
C	0.12225100	1.20077500	0.57013800
C	1.06379800	-1.58102500	0.33376000
H	-2.24570800	-0.66527400	0.52329100
H	-0.45415600	-1.46816200	1.98943000
H	-0.55982300	0.81490400	-1.42993600
H	0.56204500	2.18517600	0.67287200
H	0.80441900	-2.64061500	0.16632900
H	1.92547300	-1.56344900	1.02161600
H	-0.41397700	-1.51484600	-1.40337200
H	-2.56101000	1.50716100	0.42943800
H	2.92680600	1.68147100	-1.26623200
H	2.07411800	0.43706500	-0.74852700

6.2.3 C−C scission

C1

0 1

C	-0.63120100	0.64195700	-0.00194600
H	-1.19606300	1.56357800	-0.02903300
C	0.69665600	0.60791400	0.00855200
H	1.29973000	1.50392900	-0.00045300
O	-1.31607100	-0.55664700	-0.07250000

H	-2.10877100	-0.51593800	0.46650800
O	1.41814000	-0.53999000	0.01224200
H	0.79582600	-1.27769200	0.00540300

C2

0 1

C	1.03427500	-0.50220400	0.38868000
C	-1.29923500	0.33643600	0.16064200
C	0.13964300	0.50291600	-0.31920700
H	1.12283500	-0.34596600	1.48311800
H	-1.93364600	1.05477800	-0.36825500
H	-1.34105500	0.57120000	1.22621400
H	0.21115300	0.32165400	-1.39898000
O	1.61986700	-1.38358300	-0.17395100
O	0.51856700	1.82101600	0.02661500
H	1.35437600	2.03707400	-0.39466800
H	-1.79011100	-1.22086300	-0.91195700
O	-1.74739000	-0.99252900	0.02031700

FD

0 1

O	0.00000000	0.00000000	0.67105800
C	0.00000000	0.00000000	-0.52560500
H	0.00000000	0.93942400	-1.10741700
H	0.00000000	-0.93942400	-1.10741700

TS-C

0 1

C	-0.93096800	-0.16031800	0.53180400
C	-2.40913700	-0.36268500	0.17136900
C	2.06872500	0.00449700	0.44746700
C	1.49887600	1.04032900	-0.26926000
C	-0.12389800	-0.07721500	-0.75472400
H	-2.51004000	-1.22132300	-0.49645900
H	-2.96248200	-0.57547700	1.09138000
H	-0.82710900	0.76802800	1.10106100
H	2.08174400	0.03759400	1.53871900
H	1.76266600	1.16808100	-1.31372900
H	-0.44775900	0.71198100	-1.44136400
O	-2.93388800	0.75474200	-0.52054700
O	2.40134000	-1.09079700	-0.14659600
H	-3.02898700	1.48397200	0.09721900
O	1.01748700	2.13968200	0.41218100
H	1.39777800	2.94277000	0.04812600
O	0.30384000	-1.18031700	-1.24446600
H	1.55215400	-1.31089700	-0.81179700
O	-0.46262700	-1.21400700	1.33508800
H	-0.24877100	-1.94680400	0.74163200

TS-C2C1

0 1

C	-0.15180000	1.10458800	0.25753800

C	0.51196100	-1.26149300	0.24919600
C	-0.86384800	0.12013700	-0.39564400
H	-0.54742700	1.50487400	1.19571600
H	-0.01496700	-2.00395300	-0.35907900
H	0.23783400	-1.27919600	1.31189400
H	-0.66049200	-0.09291700	-1.43957100
O	1.04992600	1.40849100	-0.09760400
O	-2.06971900	-0.28544500	0.13004100
H	-2.72926100	-0.34154200	-0.56510500
H	1.57242300	0.42134900	-0.18469400
O	1.66529500	-0.87154800	-0.11065000

TS-C1HAA

0 1

C	-0.45761200	-0.34900700	0.13044600
H	-0.41470100	-1.07774000	0.94219000
C	0.66846700	0.50838600	0.14162400
H	0.73059800	1.58627500	0.29952400
O	-1.71219100	0.11158500	-0.23591300
H	-2.33268000	-0.00204700	0.48767200
O	1.71073700	-0.16908900	-0.13277100
H	0.76327900	-1.00272900	-0.41233400

6.2.4 Isomerisation

D1

0 1

C	-0.20990500	0.55666300	0.43363200
C	1.03738600	-0.17058300	-0.03573600
C	2.37277100	0.48758100	0.21228100
C	-2.24469400	-0.94309500	0.11431500
C	-1.43596100	0.22433200	-0.45415100
H	2.43906400	0.72589500	1.28148500
H	-0.40611700	0.20026500	1.45656600
H	-2.95241900	-1.29009300	-0.64632100
H	-2.82234900	-0.55602100	0.95751300
H	-1.08681700	-0.00902500	-1.46578300
H	2.37603400	1.44519600	-0.32059700
O	0.08955800	1.93410200	0.43188500
H	-0.71864900	2.39976400	0.67170100
O	-2.31936200	1.33458400	-0.48289100
H	-2.12402700	1.87711300	-1.25008300
O	0.98158000	-1.25016500	-0.58267800
H	3.05673600	-1.13365300	-0.55849400
O	3.43435600	-0.32365900	-0.19241500
H	-0.77397400	-2.21481800	-0.03861800
O	-1.44926500	-1.99161300	0.61242200

D1-a1

0 1

C	1.79573300	0.09251100	0.08582800
C	1.10878400	-0.40573800	1.38360200

C	-1.94708500	0.49647500	-0.34815500
C	-0.54411000	-0.05681300	-0.17997000
C	0.63509100	0.79735100	-0.64284400
H	1.31312000	-1.46939100	1.52211900
H	1.41397300	0.13934100	2.27728800
H	2.59393300	0.80608400	0.28750100
H	-2.04702200	1.44458300	0.18341100
H	-2.14785300	0.65665100	-1.40757000
H	0.76964400	0.79562100	-1.72275100
O	-0.30147600	-0.18524600	1.21000700
O	-2.87891200	-0.46804000	0.11000300
H	-1.24062800	-1.79548600	-0.63901300
O	-0.42830900	-1.30383500	-0.81973600
H	-2.76924200	-0.54727900	1.06341200
O	0.47684800	2.13570000	-0.22612900
H	0.29123300	2.13968300	0.71933700
O	2.36675600	-0.94945300	-0.66765700
H	1.65311800	-1.54552600	-0.92641100

D1-a2

0 1

C	-1.49361900	-0.17881200	0.27470600
C	-1.43827700	1.31869200	-0.03923800
C	2.03777100	0.67456700	-0.03649600
C	0.71027200	0.60436000	-0.02265000
C	-0.12275000	-0.63591300	-0.23561000
H	-1.67341000	1.48804900	-1.09986900

H	-2.10389500	1.92070100	0.57588100
H	-1.53094700	-0.32021000	1.35951400
H	2.56618500	1.60394800	0.12905200
H	-0.19847000	-0.88415800	-1.30168500
O	-0.09694300	1.69786800	0.23762200
O	2.85863700	-0.39059100	-0.25761800
H	2.33716900	-1.20364900	-0.22688200
O	0.41443200	-1.80027300	0.36487600
H	0.43710300	-1.66857500	1.31911600
O	-2.60529000	-0.85492100	-0.24417600
H	-2.56081700	-0.85013500	-1.20502700

D1-a3

0 1

C	-1.75759500	0.67212300	0.21455600
C	-2.00374500	-0.60083700	-0.09293600
C	1.44443000	-0.98165100	-0.04596100
C	0.18748100	-0.57366800	0.08682500
C	-0.27377800	0.82934100	0.41249500
H	-2.93710700	-1.10728500	-0.28915200
H	-2.48974500	1.45271800	0.34484000
H	1.70894300	-2.00015100	-0.29777800
H	-0.01196500	1.12589700	1.43316300
O	-0.90234400	-1.40172300	-0.16073800
O	2.50966200	-0.15917400	0.13200500
H	2.20952400	0.75879700	0.07907700
O	0.37110500	1.81551500	-0.39719200

H	0.11220700	1.66123800	-1.31262300

D1-b1

0 1

C	1.52991900	0.46031800	0.00000000
C	0.00000000	0.43916200	0.00000000
C	-0.68710500	-0.90103000	0.00000000
H	-0.34064600	-1.45884400	0.87909100
H	1.97278000	1.47242200	0.00000000
H	-0.34064600	-1.45884400	-0.87909100
O	2.17435700	-0.54775300	0.00000000
O	-0.60562000	1.47964600	0.00000000
H	-2.28846200	0.17043600	0.00000000
O	-2.07622600	-0.77137600	0.00000000

AD

0 1

H	-1.57722800	-0.66927300	0.87917200
C	-0.92900900	-0.71783200	0.00000000
H	-0.36309200	-1.64767000	0.00000000
H	-1.57722800	-0.66927300	-0.87917200
C	0.00000000	0.46274700	0.00000000
H	-0.48972300	1.45770700	0.00000000
O	1.19766500	0.38237700	0.00000000

ETH

0 1

C	0.00000000	0.44293100	0.00000000
C	1.20823100	-0.10820300	0.00000000
H	1.34970900	-1.18350000	0.00000000
H	2.08589400	0.52113700	0.00000000
H	-0.15406200	1.51529600	0.00000000
O	-1.18751300	-0.21242700	0.00000000
H	-1.03082100	-1.16188700	0.00000000

D1-c1

0 1

C	-1.17303900	-0.72296000	-0.01323000
C	-0.06414900	-0.01439900	0.19088500
C	1.29166000	-0.57593000	0.44694800
H	1.26623000	-1.66788800	0.38933200
H	-1.18272000	-1.80352000	0.00331800
H	1.64519800	-0.28743100	1.43975200
O	-2.38943300	-0.16463500	-0.22838800
H	-2.27911300	0.79437700	-0.22642500
H	0.68620700	1.72170800	-0.08123900
O	-0.16412500	1.35956000	0.19354000
H	2.03255300	-0.32405600	-1.34276600
O	2.24165900	-0.01410800	-0.45635200

DHA

0 1

C	1.29858400	-0.72188300	0.08319600
C	0.00000000	0.04864300	-0.00000200
C	-1.29858400	-0.72189400	-0.08310000
H	-1.30747800	-1.49735000	0.69074300
H	1.30747600	-1.49744200	-0.69054400
H	-1.30573100	-1.23438800	-1.05619300
O	2.40981000	0.10876000	-0.06142000
H	2.10115900	1.01981500	0.02843300
H	1.30573200	-1.23424800	1.05635600
O	0.00000000	1.25679800	-0.00008300
H	-2.10115800	1.01981100	-0.02856800
O	-2.40981000	0.10876800	0.06140400

D1-d1

0 1

C	-0.24480100	1.00424200	-0.01893300
C	0.69017400	-0.15535400	-0.01271100
C	2.17130600	0.13986300	0.05108600
C	-2.29924100	-0.37505100	0.54143800
C	-1.55238400	0.90613600	0.23946400
H	2.35842700	0.77836700	0.92280100
H	-3.29730300	-0.11779600	0.89513000
H	-1.78817800	-0.91905600	1.34307700
H	-2.13251700	1.82681800	0.22417700
H	2.43098000	0.73410800	-0.83386000

O	0.42062800	2.16753900	-0.28013300
H	-0.18521000	2.91306100	-0.23309700
O	0.30094900	-1.30464200	-0.05065000
H	2.31197700	-1.77510000	0.05791200
O	2.92571500	-1.03138600	0.11670800
H	-1.62311800	-1.60128100	-0.77009500
O	-2.48046700	-1.20127800	-0.58693700

D1-d2

0 1

C	0.42345200	0.93860400	0.21775300
C	-0.78443100	-0.01446600	0.31646400
C	-2.02554200	0.37446800	-0.44174800
C	2.45072800	-0.37765900	-0.30912600
C	1.73057000	0.44047800	0.77405200
H	-1.75095900	0.41405100	-1.50526000
H	3.36820000	-0.80635200	0.10655300
H	2.72374600	0.27759500	-1.13738600
H	2.33875400	1.30110400	1.05522600
H	-2.30995300	1.39157900	-0.15400100
O	0.27496100	2.00296000	-0.32272800
H	1.54357700	-0.18430200	1.65012600
O	-0.72310400	-1.03057900	0.96376500
H	-2.71912700	-1.27877200	0.25979500
O	-3.07609600	-0.51510200	-0.21015300
H	1.30027400	-1.94904300	-0.15884200

O	1.61634200	-1.37408100	-0.86345700

D1-d3

0 1

C	-0.97506900	0.85241000	-0.04248100
C	0.31580200	0.03493700	0.13941400
C	1.53532700	0.58773800	-0.57094200
C	-1.37257300	-1.49627400	-0.08380000
C	-2.10043700	-0.15588100	-0.21742700
H	1.73261400	1.59346800	-0.20091500
H	-1.72677700	-2.26175700	-0.77005200
H	-1.41357200	-1.87353800	0.94070000
H	-2.87982200	0.01523600	0.52483100
H	1.35149200	0.62584600	-1.64811400
O	-1.03845700	2.04564200	-0.07665900
H	-2.53172800	-0.01779800	-1.21148200
O	0.52508900	-0.07016300	1.51626400
H	2.52745500	-1.07581200	-0.65604400
O	2.66003100	-0.21115400	-0.25370300
H	1.43279000	-0.37846300	1.64071200
O	-0.01050700	-1.20991900	-0.43193000

D1-d4

0 1

C	0.86687600	0.83994900	-0.01662400

C	-0.40304600	0.09958700	-0.03585700
C	-1.63891600	0.60195500	0.02682500
C	1.18539300	-1.51085500	0.20841500
C	1.94749200	-0.23309500	-0.13738700
H	1.23004000	-1.71902300	1.28145600
H	1.50515300	-2.39003000	-0.34588200
H	2.29847100	-0.23891400	-1.17300500
H	-1.80850200	1.66781600	0.08808300
O	1.02771000	2.02998600	0.06867300
H	2.78929500	-0.01837700	0.51844100
H	-2.49070000	-1.08075900	-0.04390600
O	-2.75029900	-0.15177200	0.00718000
O	-0.18623000	-1.25395800	-0.15053000

D1-d5

0 1

C	-0.51788100	-1.58967100	-0.28998000
C	0.34907300	0.23942100	0.77200600
C	-0.91996800	0.72268600	0.04450100
C	-1.60705900	-0.53223900	-0.46620900
H	-0.89685100	-2.58224000	-0.05878000
H	0.39832500	0.67595900	1.77350600
H	-1.96216300	-0.39042300	-1.48584100
O	-1.24791200	1.86464100	-0.09528300
H	-2.46449700	-0.73059000	0.18303400
O	0.23955600	-1.14940300	0.84289000
H	0.13466800	-1.64324000	-1.16641700

C	1.55134200	0.71593800	-0.03992100
H	1.70555300	1.81195600	-0.03304500
O	2.25234600	-0.02501700	-0.66446100

D1-d6

0 1

C	0.98019300	-0.71637700	0.00000000
C	0.00000000	0.41794800	0.00000000
C	-1.47298500	0.07030200	0.00000000
C	2.29148800	-0.49896000	0.00000000
H	-1.67724400	-0.55229800	0.87952400
H	2.64640300	0.52192800	0.00000000
H	-1.67724400	-0.55229800	-0.87952400
O	0.35383600	-1.92531600	0.00000000
H	3.01070800	-1.30930600	0.00000000
O	0.35870600	1.57077800	0.00000000
H	-1.66869100	1.97816100	0.00000000
O	-2.26606800	1.21852700	0.00000000
H	1.00210100	-2.63557800	0.00000000

D1-d7

0 1

C	0.68631800	-1.01290200	0.00000000
C	0.00000000	0.36795700	0.00000000
C	-1.50782400	0.38918300	0.00000000

C	2.18504200	-1.01235500	0.00000000
H	-1.85562600	-0.16770000	0.87849800
H	2.55195600	-0.47175200	0.87534100
H	-1.85562600	-0.16770000	-0.87849800
O	-0.00689100	-1.99594800	0.00000000
H	2.55195600	-0.47175200	-0.87534100
O	0.65262900	1.38046600	0.00000000
H	-1.25107700	2.29735000	0.00000000
O	-2.00472700	1.69388300	0.00000000
H	2.54911000	-2.03695800	0.00000000

DFO

0 1

C	1.39058800	0.59473200	0.21828800
C	-0.00748800	-1.20394900	0.06119500
C	-0.89534200	0.03618700	-0.01013700
C	0.04635300	1.22574200	-0.13883600
H	2.24690100	1.04916400	-0.27519100
H	-0.31311700	-1.96367500	-0.65770600
H	-0.27285500	2.04827300	0.49931500
O	-2.09171400	0.05326000	0.04402700
H	0.03098200	1.55791300	-1.18067800
O	1.29439600	-0.75281500	-0.23763700
H	1.55260300	0.61046400	1.30384100
H	-0.07063900	-1.62197400	1.07624600

TS-D

0 1

C	-0.01974300	-0.78730300	0.35736200
C	-1.33238400	-0.13512400	-0.10282100
C	-1.73245800	1.22828300	0.15930000
C	1.61401100	1.18309500	0.18772600
C	1.26482900	-0.23133200	-0.24855400
H	-1.07745900	0.55936900	-1.11992100
H	0.03268900	-0.70332000	1.45173300
H	1.55564000	1.24623800	1.28389200
H	0.91710700	1.90261800	-0.24828200
H	1.17992200	-0.27126200	-1.34370300
H	-1.10204900	2.09365500	0.32339900
O	-0.07339900	-2.14299700	-0.01271300
H	-1.01267600	-2.36110100	-0.12446700
O	2.34842700	-1.03329200	0.17902500
H	2.12059700	-1.95328400	0.00216800
O	-2.36040200	-0.90791500	-0.24900500
H	-3.27672500	0.38266300	-0.04209800
O	-3.00207700	1.37959000	0.07846000
H	3.47918300	0.81177500	-0.07393900
O	2.88973100	1.54798100	-0.27412400

TS-D1a1

0 1

C	-1.56372000	-0.13596900	-0.42340600
C	-1.09673400	-1.57092900	-0.20767300

C	1.69898200	0.72820800	-0.61439300
C	0.74773400	0.05655200	0.35478700
C	-0.64940400	0.68051500	0.48197000
H	-1.40891400	-1.92110600	0.78109800
H	-1.43999500	-2.26730200	-0.97302900
H	-1.36594500	0.16659700	-1.45964600
H	1.26736600	0.78052200	-1.61490300
H	1.82781800	1.75456900	-0.24655600
H	-0.97376200	0.55518100	1.52171200
O	0.32759400	-1.45351400	-0.27911900
O	2.91271000	0.03377700	-0.67893000
H	0.87317600	-1.53976100	0.74073700
O	1.30214100	-0.49499900	1.41528800
H	3.09904600	-0.26438500	0.22112400
O	-0.60550700	2.03810300	0.11265300
H	-1.48835400	2.40177700	0.23102600
O	-2.90255900	0.11334500	-0.06444100
H	-3.48661700	-0.11005700	-0.79288100

TS-D1a12

0 1

C	-1.68185300	0.38934300	-0.16745700
C	-1.49253900	-0.99431300	-0.79072000
C	1.99965300	-0.35520900	-0.38493900
C	0.57518600	-0.26021800	-0.44698800
C	-0.26051200	0.77097200	0.27722900

H	-1.57039000	-1.76014600	-0.01522900
H	-2.12413100	-1.21003300	-1.64715400
H	-1.99178200	1.11478900	-0.92778700
H	1.85467700	-1.24579900	0.45010400
H	2.49178200	-0.70102900	-1.29206600
H	-0.13109400	0.66839500	1.35429900
O	-0.12049200	-1.00026000	-1.24742700
O	2.58321000	0.79984600	0.18663600
H	0.42109000	-2.25873800	1.92376100
O	0.74783200	-1.61778600	1.28538400
H	2.96656800	0.55072500	1.03149600
O	0.05812100	2.05721100	-0.20815900
H	0.99064300	2.21376200	-0.00657300
O	-2.63158500	0.28146200	0.86130500
H	-2.84366000	1.16085100	1.18448400

TS-D1a23

0 1

C	-1.43407900	0.52494500	-0.31327800
C	-1.55578100	-0.82622500	-0.71890600
C	1.85399300	-0.89855300	0.24706200
C	0.60041600	-0.59321900	-0.07331000
C	0.01595900	0.78962500	0.05280300
H	-2.22092000	-0.77399400	0.55163100
H	-2.12670900	-1.14946900	-1.57762800
H	-2.07717000	1.32631700	-0.65037400

H	2.25136400	-1.90317100	0.19548600
H	0.14071200	1.19344200	1.06218600
O	-0.29848600	-1.48134300	-0.59784000
O	2.74658500	0.05123100	0.66849900
H	2.55490500	0.87657800	0.20944200
O	0.64708800	1.65409800	-0.88825100
H	0.34209500	2.55536400	-0.74160800
O	-2.32457300	0.22658300	1.27219900
H	-1.91223700	0.29093800	2.14778800

TS-D1a3FF

0 1

C	-1.61140300	0.93127300	0.22500000
C	-2.07850400	-0.25713700	-0.22661300
C	1.25259500	-1.17257900	0.04165800
C	0.00162000	-0.66036600	0.25118800
C	-0.21719800	0.72821700	0.55510100
H	-3.06600500	-0.53807300	-0.56179000
H	-2.18651800	1.83393300	0.34477900
H	1.41119500	-2.21936900	-0.22186200
H	0.24595600	1.18701200	1.42153900
O	-1.17352600	-1.23983000	-0.22393600
O	2.26473600	-0.38092400	0.10231200
H	1.77721500	0.69420000	-0.24073900
O	0.97299400	1.52484600	-0.57568200
H	1.22187000	2.39311400	-0.24148300

TS-D1b1

0 1

C	0.22029200	-0.09392400	1.28355100
C	-1.03590100	0.21697700	0.49418000
C	-1.67460400	-0.87150300	-0.32956200
C	2.29151000	0.14690400	-0.58106300
C	1.45729800	1.05222500	0.08404300
H	-1.81072400	-1.75173700	0.30884800
H	0.30254900	0.57547200	2.14866400
H	3.09203100	-0.31279700	-0.01734700
H	2.42096200	0.24949200	-1.65057800
H	1.89475200	1.47816000	0.98622900
H	-0.96091900	-1.15087000	-1.11515900
O	0.64266100	-1.28435800	1.38910200
H	1.02127200	-1.67123100	0.10819600
O	0.80757600	1.94688000	-0.74393000
H	-0.00258200	2.25730600	-0.31863100
O	-1.52438100	1.32787100	0.54068500
H	-2.98427300	0.48679500	-0.69619700
O	-2.89503600	-0.45852900	-0.87188700
H	2.06128200	-2.28926300	-0.98917500
O	1.39594000	-1.60378900	-0.87293800

TS-D1b1HAA

0 1

C	0.72480700	-0.81366400	-0.00004800
O	2.12376300	-0.80573200	0.00001600
C	0.14497300	0.59642200	0.00000600
H	2.40611600	0.11864000	0.00004800
H	0.33678500	-1.33883700	0.88149400
H	0.33686000	-1.33872800	-0.88169000
O	0.86058700	1.55936700	0.00007200
C	-2.05812200	0.39600200	-0.00002300
H	-1.48828100	1.32866300	-0.00002700
O	-2.29202700	-0.73392200	-0.00001800

TS-ETHAD

0 1

C	-0.11626800	0.52982500	0.04738900
C	1.10288000	-0.17491100	-0.04719300
H	1.25857100	-0.81994500	0.81818700
H	2.00784200	0.27987400	-0.43204100
H	-0.30943700	1.60194300	0.12647400
O	-1.09815800	-0.27328900	-0.02176000
H	-0.09138200	-1.00504100	-0.33971900

TS-D1c1

0 1

C	0.17707900	0.69672800	-1.03021300
C	-1.06032500	0.44124300	-0.48309600

C	-1.88985300	-0.75501700	-0.87181500
C	2.16377000	-0.48253100	0.43669600
C	0.70970600	-0.42048000	0.81215500
H	-1.29796400	-1.45934500	-1.45929200
H	0.61400700	0.09386800	-1.81446200
H	2.67871000	-0.91642200	1.30432200
H	2.53935800	0.53277300	0.27694100
H	0.15043700	-1.35566300	0.69781600
H	-2.72768500	-0.40875000	-1.49068000
O	0.82465800	1.85723300	-0.79112400
H	0.30002100	2.36375100	-0.15414400
O	0.37407700	0.41269700	1.72573200
H	-0.65551300	0.88232600	1.35826100
O	-1.48172000	1.17851400	0.50569400
H	-2.73597900	-0.76860600	0.85875300
O	-2.35396100	-1.43323400	0.27376700
H	3.22443100	-1.53315200	-0.82286200
O	2.30043600	-1.30401400	-0.70619600

TS-D1c1DHA

0 1

C	1.34849900	-0.24549600	0.60783600
C	0.01298700	0.13727700	0.17382700
C	-1.17843400	-0.72614000	-0.00323500
H	-1.19225800	-1.41588000	0.85301400
H	1.41984600	-1.10689600	1.27406700

H	-0.96634500	-1.33235800	-0.89717900
O	2.19154700	-0.41501400	-0.53892400
H	2.97298900	0.12424200	-0.41517200
H	1.05892400	1.28744100	0.50899600
O	-0.00736400	1.38231200	-0.03850900
H	-3.06495100	-0.53664900	-0.41604200
O	-2.34999700	0.03098300	-0.11984900

TS-D1d1

0 1

C	0.16044200	0.84445500	0.10596600
C	-0.94281200	-0.02441200	-0.27089700
C	-2.31374100	0.29346800	0.31401400
C	1.87749800	-1.13341400	-0.04925900
C	1.53168400	0.33377800	0.14138100
H	-2.56385300	1.34037600	0.10488700
H	0.99730200	1.37922500	-1.15319000
H	2.95810300	-1.23143500	-0.19902000
H	1.35529900	-1.53214000	-0.91375300
H	2.10616500	0.81091900	0.93716000
H	-2.24640000	0.19101600	1.40396700
O	-0.10811000	1.77075000	1.14619700
H	-0.42266500	2.59197900	0.76470800
O	2.07122700	1.04545500	-1.23500200
H	2.71337300	1.75632000	-1.08473500
O	-0.84634200	-0.95490700	-1.05940700
H	-2.81279700	-1.17608500	-0.79031300

O	-3.28825500	-0.55259400	-0.22125100
H	1.95844700	-1.62084200	1.84201100
O	1.43130400	-1.85777800	1.07459300

TS-D1d2

0 1

C	0.21437600	0.96603400	-0.04058300
C	-0.80708900	-0.14232000	0.05586100
C	-2.26151000	0.23604800	-0.02563300
C	2.43076900	-0.28344500	0.43016600
C	1.61668000	0.86536700	-0.11317700
H	-2.40840300	0.86983400	-0.90759800
H	2.05841600	-0.56599000	1.42077600
H	3.46389100	0.04391600	0.54855200
H	1.98590700	1.31144200	-1.04106900
H	-2.48215800	0.86439700	0.84886900
O	-0.17057600	2.18272400	-0.01597700
H	1.12713400	2.23886500	0.14251200
O	-0.44934800	-1.28636600	0.20647900
H	-2.56547100	-1.65725600	0.16310900
O	-3.08802000	-0.88460500	-0.08481000
H	1.58702800	-1.79672000	-0.42116500
O	2.46723100	-1.40657600	-0.42991600

TS-D1d3

0 1

C	-0.88698200	0.92121200	0.05137300
C	0.39721900	0.09773500	0.21107100
C	1.58627500	0.51914500	-0.62304300
C	-1.57570800	-1.28505400	-0.54161300
C	-2.07968800	-0.01063100	0.13642100
H	1.73118000	1.58823600	-0.41568400
H	-1.68606200	-1.23269500	-1.62479000
H	-2.03910100	-2.20049600	-0.17897900
H	-2.28672500	-0.18579000	1.19597700
H	1.37565600	0.40225300	-1.68747000
O	-0.89971400	2.10798600	-0.10715000
H	-2.95761800	0.42488500	-0.33592100
O	0.64579000	-0.31207300	1.44407800
H	2.67338100	-0.38985000	0.65640500
O	2.71253600	-0.24365000	-0.29838400
H	0.13131300	-1.40593100	0.83281700
O	-0.15720200	-1.35914500	-0.26949500

TS-D1d4

0 1

C	-0.94190300	0.78858200	-0.33225600
C	0.35113100	0.00710500	-0.38181000
C	1.64852600	0.56046100	-0.19678400
C	-1.23186200	-1.55373900	-0.00151400
C	-2.02420600	-0.27664300	-0.23340300

H	1.31162500	0.70698300	0.97171200
H	-1.55775400	-2.42064900	-0.56839000
H	-1.12377800	-1.76911900	1.06168300
H	-2.70840100	-0.04411600	0.57938600
H	1.78494800	1.55018400	-0.62391000
O	-1.05042400	1.96641300	-0.49609800
H	-2.57595500	-0.28993400	-1.17637200
O	0.12733600	0.26430200	1.80431100
H	2.68313600	-1.01038100	0.21538700
O	2.75140900	-0.26294500	-0.38697100
H	-0.33103400	0.45480900	2.62874400
O	0.13506600	-1.25931700	-0.44794600

TS-D1d5

0 1

C	0.64134300	0.91038500	-0.05576100
C	-0.42879000	-0.09170500	-0.10112900
C	-1.75559500	0.18926000	0.42824100
C	1.45718500	-1.31988500	-0.15881100
C	1.94456700	0.11764300	0.02617700
H	2.01607900	-2.06332700	0.40574400
H	1.44909700	-1.59102900	-1.22114500
H	2.66243700	0.45382500	-0.72082800
H	-2.07322900	0.75494400	1.30595600
O	0.51376100	2.11330400	-0.07649900
H	2.36883800	0.28239500	1.02039300

H	-1.65890600	-0.50174300	-1.02760300
O	-2.61922700	-0.30036500	-0.33441000
O	0.11589400	-1.33409600	0.33655500

TS-DFO

0 1

C	-2.01669700	-0.25196100	-0.39562000
C	-0.14459500	-0.48759000	0.91075700
C	-0.03083200	0.82706500	0.34082700
C	-1.07054500	0.87732200	-0.78709800
H	-2.81329800	0.11669200	0.25836800
H	0.08364500	-0.70374300	1.94922400
H	-0.56422300	0.68675900	-1.74049100
O	0.81016700	1.67236800	0.58289900
H	-1.54062800	1.85786200	-0.83693200
O	-1.21828000	-1.18887600	0.35804900
H	-2.45673200	-0.79290400	-1.23120800
C	1.68540000	-1.13859500	-0.31093800
H	0.86646400	-1.73198500	0.10095200
O	2.39416100	-0.28225800	-0.57188300

TS-D1d6

0 1

C	-0.41085000	0.12687300	-0.66690700
C	0.99029600	0.38147300	-0.14362200
C	1.89241600	-0.81783500	0.00810500

C	-2.28032400	0.27925600	0.93554000
C	-1.34144300	1.14296500	-0.67766900
H	1.95915800	-1.32038900	-0.96472300
H	-3.05630300	1.04796900	0.87142600
H	-1.52792200	0.45077700	1.71521800
H	-1.01713000	2.14239400	-0.41649600
H	1.40285100	-1.52673000	0.68793700
O	-0.72563500	-1.10292400	-0.89575700
H	-2.22509700	1.02839300	-1.29153700
O	1.36432200	1.49378600	0.13493000
H	3.15792700	0.50330200	0.60212500
O	3.15421100	-0.45454200	0.47934600
H	-1.66237300	-1.25085600	-0.27981900
O	-2.55935700	-0.90522600	0.57437800

TS-D1d7

0 1

C	1.15563300	-0.10653700	-0.00227500
C	-0.28664500	0.32987200	-0.06931900
C	-1.33894300	-0.73728800	0.11675100
C	2.30437900	0.65592300	0.26774600
H	-1.11758300	-1.29386800	1.03478600
H	2.83836500	0.30046500	1.14900400
H	-1.23664700	-1.44655800	-0.71509300
O	1.45581800	-1.31694900	-0.27479800
H	2.34573700	1.72357800	0.09569700

O	-0.57054300	1.48010700	-0.28367300
H	-2.55777900	0.73636600	-0.09898600
O	-2.61923700	-0.18719000	0.17616000
H	2.59305400	-0.67954500	-0.28435800
